# Impacts of economic inequality on healthcare worker safety at the onset of the COVID-19 pandemic: cross-sectional analysis of a global survey

**DOI:** 10.1136/bmjopen-2022-064804

**Published:** 2022-10-05

**Authors:** Sean P Harrigan, Vivian W L Tsang, Annalee Yassi, Muzimkhulu Zungu, Jerry M Spiegel

**Affiliations:** 1 Global Health Research Program, School of Population and Public Health, The University of British Columbia, Vancouver, British Columbia, Canada; 2 Faculty of Medicine, The University of British Columbia, Vancouver, British Columbia, Canada; 3 National Institute for Occupational Health, Johannesburg, Gauteng, South Africa; 4 School of Health Systems and Public Health, University of Pretoria Faculty of Health Sciences, Pretoria, Gauteng, South Africa

**Keywords:** COVID-19, Public health, Health & safety, Human resource management, OCCUPATIONAL & INDUSTRIAL MEDICINE

## Abstract

**Objectives:**

To assess the extent to which protection of healthcare workers (HCWs) as COVID-19 emerged was associated with economic inequality among and within countries.

**Design:**

Cross-sectional analysis of associations of perceptions of workplace risk acceptability and mitigation measure adequacy with indicators of respondents’ respective country’s economic income level (World Bank assessment) and degree of within-country inequality (Gini index).

**Setting:**

A global self-administered online survey.

**Participants:**

4977 HCWs and healthcare delivery stakeholders from 161 countries responded to health and safety risk questions and a subset of 4076 (81.2%) answered mitigation measure questions. The majority (65%) of study participants were female.

**Results:**

While the levels of *risk* being experienced at the pandemic’s onset were consistently deemed as unacceptable across all groupings, participants from countries with less income inequality were somewhat less likely to report unacceptable levels of risk to HCWs regarding both workplace environment (OR=0.92, p=0.012) and workplace organisational factors (OR=0.93, p=0.017) compared with counterparts in more unequal national settings. In contrast, considerable variation existed in the degree to which *mitigation* measures were considered adequate. Adjusting for other influences through a logistic regression analysis, respondents from lower middle-income and low-income countries were comparatively much more likely to assess both occupational health and safety (OR=10.91, p≤0.001) and infection prevention and control (IPC) (OR=6.61, p=0.001) protection measures as inadequate, despite much higher COVID-19 rates in wealthier countries at the time of the survey. Greater within-country income inequality was also associated with perceptions of less adequate IPC measures (OR=0.94, p=0.025). These associations remained significant when accounting for country-level differences in occupational and gender composition of respondents, including specifically when only female care providers, our study’s largest and most at-risk subpopulation, were examined.

**Conclusions:**

Economic inequality threatens resilience of health systems that rely on health workers working safely to provide needed care during emerging pandemics.

**Strengths and limitations of this study**

- A major strength of the study is its novel empirical testing of the ‘income inequality’ hypothesis for a comparative cross-country analysis of a major global health challenge: protection for a workforce central to the provision of healthcare services during a pandemic.
- This study is based on a unique global self-administered online survey conducted by a network of occupational health experts coordinated by WHO through a large array of professional networks and social media.
- A major limitation of the study is its character as a convenience sample with different compositions by gender and occupation among countries and small sample sizes in some countries; however, access to gender and occupation identifiers of respondents has enabled adaptive strategies to take this into consideration.
- The study is exploratory in considering associations with economic inequality, but does not provide a way to consider pathways for this effect, so further research will be needed for this.

## Introduction

As the COVID-19 pandemic emerged, attention was quickly drawn to risks faced by front-line healthcare providers^1 2^—and the urgent need to strengthen their protection.^3 4^ By September 2020, it was estimated that 10% of global infections had been in healthcare workers (HCWs), and over 7000 had died.^5 6^ Notwithstanding inconsistent reporting, Papoutsi and colleagues, in reviewing the global burden of COVID-19 for HCWs by country,^6 7^ estimated the percentage of HCW cases among the total cases by April 2020 as ranging from less than 1% in Hong Kong and India, to 19% in Spain.

Despite 60 million people employed in the healthcare sector worldwide,^8 9^ a global shortage of HCWs persists and is especially critical in low and middle-income countries (LMICs),^10^ where the greatest global burden of disease exists.^11^ Risk mitigation is marked by considerable variation,^12^ with shortcomings in infrastructure and mitigation programmes contributing to higher burdens of disease and HCW risk in more poorly resourced settings.^13^ The danger that HCWs face of acquiring COVID-19 adds to extensive existing risks in infectious disease endemic states, for example, with tuberculosis in sub-Saharan Africa.^14^

While lack of personal protective equipment (PPE) was highlighted early in the COVID-19 pandemic, consideration of broader occupational health and safety (OHS) factors and mitigation measures attracted less initial attention.^7 8 15^ To ascertain the extent of OHS risk exposure and the adequacy of mitigation measures in place to meet the challenge of COVID-19, an ad hoc expert group of WHO, the International Labour Organization (ILO) and the International Commission on Occupational Health prepared and circulated a questionnaire survey to identify HCW perceptions of the most common threats to their health and safety as well as the adequacy of mitigation measures in the emerging pandemic.^16^

Further to a preliminary analysis of survey results^16^ regarding risk and adequacy of protection, we sought to ascertain the degree to which perceived risk exposure of HCWs and adequacy of mitigation measures is associated with a country’s economic characteristics. Considerable attention, after all, has been given to the impact of economic disparity on health,^17 18^ especially in relation to Wilkinson’s ‘economic inequality hypothesis’ suggesting that greater inequality is associated with poorer health.^19^ In recognition that ‘the traditional exposure-disease framework used in occupational health research is not equipped to address societal contexts in which work is embedded’,^20^ we sought to examine how such driving forces^21^ as a country’s economic inequality might be affecting the well-being of HCWs.

A variety of factors have been examined that might have influenced how the onset of the COVID-19 pandemic was experienced in different national settings, including consideration of cultural traits,^22^ specific government regulations and non-pharmaceutical interventions^23 24^ and political leadership characteristics.^25^ Our study sought to apply a cross-country perspective to consider the effects of economic inequality, recognising this to be a dimension of considerable relevance in global public health research.

With this focus, we set out to first consider variation in perceptions of the acceptability of work-related risks and the adequacy of mitigation measures that were being experienced by HCWs as COVID-19 emerged; and, second, to determine the extent to which variations were associated with a country’s comparative income level and degree of income inequality.

## Methods

### Survey development

Shortly after *WHO Ad Hoc Study Group on Health and Safety of Health Workers* was established when the COVID-19 pandemic emerged, it created an online survey aimed at HCWs from all WHO regions globally. In addition to the capture of demographic indicators of respondents, the survey contained 41 questions—17 on health and safety risks and 24 on mitigation measures^16^ (online supplemental appendix). Risk questions were grouped into those related to infectious disease transmission, physical work environment, psychological work environment and work organisation. For each risk question, participants were asked, ‘*Think about the working conditions of health workers in your country, jurisdiction or health facility….; rate the current level of these risks, now during the COVID-19 pandemic*’. Questions regarding mitigation measures were divided into two groups: OHS and infection prevention and control (IPC). Here, participants were similarly asked: ‘*Think about the working conditions of health workers in your country, jurisdiction or health facility…rate the level of application of these measures according to your knowledge of the real situation now during the COVID-19 pandemic*’.

10.1136/bmjopen-2022-064804.supp1 Supplementary data

### Patient and public involvement

The participation of health workers (whose well-being is the focus for this study in relation to their assessment of the adequacy of measures to protect them) was indirectly included through the participation of their representatives (unions within the ILO and other professional bodies) who were directly involved in the creation of the research instrument and in the dissemination of the online survey and its initial results.

### Study population and inclusion criteria

Participants were recruited by convenience sampling, with dissemination through a large array of professional networks and social media. The survey, self-administered online to enable rapid low-cost recruitment, was available in Arabic, Chinese, English, French, German, Italian, Portuguese, Russian, Spanish and Swahili. A range of HCWs and stakeholders involved with healthcare delivery were invited to participate. In addition to HCWs in direct patient care in both formal and informal settings and in public and private facilities, respondents also included allied health and supporting staff, including OHS and IPC professionals, administration, management, drivers, public health workers, community health workers and others as defined by the International Standard Classification of Occupations 2008. Data collection occurred between 5 May and 25 June 2020. Participant results were excluded if they failed to complete demographic questions or if they failed to provide any responses to the risk and mitigation questions. As the survey was designed to be completed and submitted anonymously, no formal request for signed consent was solicited, with participants’ submission itself indicating consent to use the information provided as anonymised aggregated data. This work was supported by the International Development Research Centre under grant M20-00559 and the Canadian Institutes of Health Research under grant vs1-175519 for the ‘Protecting healthcare workers from COVID-19: a comparative contextualized analysis’ research programme.

### Independent variables

Demographic information for individual survey respondents was collected on country, gender and occupation—the latter separated into 13 categories and then grouped into patient care/health services; specialised technical support; clerical support/administration and management; and other. Details about the study sample population composition and demographic characteristics of participants are presented in online supplemental figure S1; Table S1, respectively.

10.1136/bmjopen-2022-064804.supp2 Supplementary data

10.1136/bmjopen-2022-064804.supp3 Supplementary data

Our research group, drawn from two WHO collaborating centres participating in the survey process, conducted the analysis by consolidating respondents by their home country and then linking this to a WHO geographic region^26^; a comparative country-level economic classification by World Bank income groups based on the annual Atlas gross national income per-capita estimates^26 27^; and the country’s Gini index—a measure used for the analysis of income inequality present within a country, with a score of 0 representing perfect equality, and a score of 1 representing complete inequality.^28^ Data for Gini and economic classification were taken from the World Bank, using the most recent data available. To take account of the variation across regions present during the initial phase of the pandemic, we also considered COVID-19 incidence per million (logarithmic scale) in each country at the time when the survey was completed, as an indicator of the intensity as of a particular date, using values for June 2020 drawn from ‘Our World in Data’ database.^29^

### Dependent variables

Acceptability of workplace risks and adequacy of mitigation measures—the dependent variables in this study—were derived from a factor analysis of individual survey responses, then aggregated to enable subsequent analysis of the effect of country-level characteristics. Factor analysis^16^ was used to reduce the 41 survey questions into coherent groupings and principal component analysis with varimax rotation carried out to create factors from each set of workplace health and safety risk exposure questions (corresponding to workplace risk and workplace organisation acceptability) and mitigation measure questions (corresponding to IPC and OHS adequacy); online supplemental table S1 summarises the subject matter covered by the questions consolidated in each factor. Separate factor analyses were run on risk questions and preventive measure questions. Missing values were excluded in a listwise fashion. The rotated component matrix was used to identify factors. To measure scale reliability, Cronbach’s alpha was used for each individual factor. Scores over 0.7 are considered to be acceptable for internal consistency.^30^ The results from the factor analysis are outlined further in our preliminary analysis.^16^

The questions were administered as a 3-point Likert scale, then converted to a 10-point scale for clearer communication (ie, midpoint of 2 becoming 5). Numerical scores were assigned to each answer to establish a scale for both the risk and mitigation measure factors, with higher scores corresponding to more desirable states. For health and safety risks, a score of 0 was assigned to ‘risk is not acceptable at all’; 5 to ‘risk is acceptable for a short time’; and 10 for ‘risk is negligible’. For mitigation measures, a score of 0 was assigned to ‘does not exist at all’; 5 to ‘exists and offers some protection’; and 10 to ‘exists and offers full protection’. Responses of ‘don’t know/unsure’ were assigned blanks. Factor scores were then calculated to form an individual respondent’s factor score for each of the four groupings, that is, work environment risk acceptability, work organisation risk acceptability, OHS adequacy and IPC adequacy, and then aggregated to generate a mean value for each country’s respondents, so that intercountry comparison could be conducted. The higher the scores, the greater the perceived adequacy of mitigation measures or acceptability of risk deemed as being experienced.

### Analysis

The mean country dependent variable factor scores derived from the aggregation of individual participants’ responses served as the basis for considering associations by WHO region, economic classification, Gini coefficients and COVID-19 incidence. Comparisons of survey mean scores were carried out using analysis of variance (ANOVA), with an alpha of 0.05 used to test significance. To compare means for the continuous variable Gini coefficient and COVID-19 incidence scores, we ordinally divided groups of countries into quartiles by values.

To ensure that intercountry variation was not purely explained by possible gender and occupational compositional differences among a particular country’s respondents, we carefully examined possible sources of discrepancy (online supplemental table S3), using ANOVA to consider effects that could complicate the cross-country comparison of all respondents. To minimise any such effect, we considered different ways to stratify our analysis of the study population, notably by focusing only on those populations that had the most direct workplace experience to personally being ‘at risk’. Noting the presence of gender differences among patient care deliverers, we specifically isolated female respondents, who in fact constituted the largest demographic group of respondents in the study, representing 1998 respondents from 112 countries (n=1968 from 112 countries), the largest subpopulation.

Finally, to measure the effect that the interaction of independent variables had on the likelihood of workplace risks being considered as acceptable in a country setting as COVID-19 was emerging, and workplace protection and control measures being deemed as adequate, we created and applied a logistic regression model. Preferred outcomes for this analysis were assessed as mean factor scores ≥5, corresponding to assessments that mitigation ‘exists and offers some protection’ or better; or ‘risk is acceptable for a short time’ or better. All statistical analyses were done using R and SPSS Statistics software (version 25).^31 32^

## Results

### Overall study population and survey responses

There were 4977 participants who responded to health and safety risk questions and a subset of 4076 (81.2%) who answered mitigation measure questions. The majority of study participants were female (65%), reflecting the make-up of the health sector workforce. Most participants were from the European region (35%), followed by the Americas (31%), the Western Pacific region (15%) and Africa (10%); the South East Asian (4%) and Eastern Mediterranean regions (3%) made up the smallest proportion of participants. In total, there were 161 countries represented in the survey. Portugal (n=549, 11%), USA (n=451, 9%), Brazil (n=373, 7%), Canada (n=263, 5%) and China (n=233, 5%) had the most participants. The majority of respondents were from countries of high-economic classification (59%), followed by upper middle (27%), lower middle (10%) and low (4%). Most survey participants worked for a health services employer (61%), followed by government services (15%) and businesses and farms (10%). Those working in academia, professional associations, international organisations and non-government organisations each encompassed less than 10%. Finally, the type of occupation was predominantly patient care/health (56%) services, followed by 29% providing technical services such as IPC or OHS specialists, 7% in administration and 10% identified as working in other sectors (online supplemental table S1).

The largest percentage of countries was in Europe (30%) and over a third of all countries were high-income countries (high income countries (HICs), 35%). The average Gini index was 37.8 (SD=7.7) and the mean and median COVID-19 incidence rates per million were 1360 and 278, respectively, at the time the survey was conducted.

Table 1 illustrates that considerable variation exists in these variables across the different WHO regions, indicating the distinct characteristics and conditions present at the onset of the pandemic. It is especially noteworthy that case levels had been far greater in high-income country areas at the survey midpoint (1 June 2020). For example, cases per million were 2525 in Europe versus 119 in Africa; 5408 in the USA; and only 138 in India and 97 in Indonesia.

**Table 1:** Country characteristics of different WHO regions

Region	Countries (n)	Countries by income classification*				Mean country values		Study population characteristics	
		High	Upper middle	Lower middle	Low	Inequality	COVID-19	Gender	Occupation
						Gini coefficient*	Cases per million†*	Female* (%)	Front-line patient care*(%)
Overall	161	57	42	36	26	37.8	1360	65.5	56.4
AFRO	37	0	6	12	19	43.2	119	44.8	52.1
EMRO	20	6	3	7	4	35.2	2407	39.8	29.4
EURO	48	32	13	2	1	31.8	2525	68.2	64.4
PAHO	30	11	14	4	1	44.8	1135	73.3	46.5
SEARO	9	0	2	6	1	35.0	86	36.7	56.3
WPRO	17	8	4	5	0	37.0	512	70.7	68.8

Full listing of WHO region countries at https://en.wikipedia.org/wiki/List_of_WHO_regions

*P<0.001.

†COVID-19 rates as of June 2020.

AFRO, Africa; EMRO, Eastern Mediterranean; EURO, Europe; PAHO, Americas; SEARO, South-East Asian; WPRO, Western Pacific.

As summarised in table 2 (full table in online supplemental table S4), the majority of respondents designated most of the health and safety risk parameters as ‘not acceptable at all’. Circumstances most reported as such included bullying or psychological harassment in the workplace (54%), physical violence and assaults (54%), exposure to blood, bodily fluids and other infectious materials (52%), inadequate sanitation facilities (52%) and sexual harassment (50%). In contrast, areas such as time pressure and high workload (38%), skin damage from PPE (33%) and shift work with night shifts (23%) were deemed to be less of a concern. There were no risk categories in which the most common response was ‘risk is negligible’.

**Table 2:** Risk acceptability and mitigation adequacy—selected worldwide survey responses

Risk acceptability	Risk is not acceptable at all (%)	Risk is acceptable for a short time (%)	Risk is negligible (%)	Don’t know/unsure (%)
Infectious risk work environment				
Exposure to blood, body fluids, respiratory secretions and other potentially infectious materials	**52**	29	15	4
Inadequate sanitation facilities	**52**	21	23	4
Skin damage from personal protective equipment and/or frequent hand hygiene	33	**46**	16	5
Physical work environment				
Crowded workplace	**42**	36	18	4
Thermal discomfort (cold, heat, humidity)	25	**46**	24	5
Psychosocial work environment				
Bullying or psychological harassment	**54**	18	21	7
Sexual harassment	**50**	10	31	9
Work organisation				
Time pressure, high workload	38	**49**	10	3
Shift work with night shifts	23	**48**	21	8

Mitigation measure adequacy	Does not exist at all (%)	Exists and offers some protection (%)	Exists and offers full protection (%)	Don’t know/unsure (%)
Infection prevention and control				
IPC policy in the health facility	8	**60**	28	4
Personal protective equipment (eg, masks, gloves, goggles, gowns) are readily available	8	**55**	34	3
Training and education of workers about infection prevention and control	11	**54**	32	3
Facilities for hand hygiene (hand washing and disinfection) are readily available	3	40	**54**	3
Occupational health and safety				
Occupational health and safety policy and management system in the facility	14	**58**	22	6
Regular assessment of workplace health and safety risks and controls	22	**51**	21	6
Engineering controls, such as ventilation, physical barriers, safer devices	19	**54**	19	8
Prevention of workplace violence and security measures	21	**52**	21	6
Workplace policies against bullying, psychological and sexual harassment	27	**43**	21	9

Most cited response highlighted in bold.

IPC, infection prevention and control.

Mitigation measures related to the above areas of concern were seen as particularly lacking, with only the category of ‘policies for facilities for hand hygiene’ designated as ‘exists and offers full protection’ (full table in online supplemental table S5). For example, despite psychosocial-related risks, including bullying, harassment, physical violence and sexual harassment, ranked consistently high (54%, 54% and 50%, respectively), only 21% indicated that corresponding policies ‘exist and offer full protection’, with similar dissatisfaction for the adequacy of mitigation measures for other key areas such as IPC policy (28%), availability of PPE (34%), as well as training and education of workers about OHS (21%) and IPC (32%). Only in two mitigation measure areas—availability of facilities for hand hygiene, and policies for postexposure prophylaxis (such as HIV or hepatitis B)—did most participants indicate that measures existed and offered full protection (54% and 42%, respectively). These results show an overwhelming majority of participants indicating that the risks they faced were not acceptable at all and that very few of the corresponding mitigation measures offered adequate protection to HCWs.

### Associations with risk exposure acceptability and mitigation measure adequacy

Unacceptable levels of *risk* (ie, factor scores below 5) were consistently reported for both work organisation and work environment across geographic regions, economic income-level categories, equity classifications and COVID-19 incidence rates, with no statistically significant differences observed within these categories (table 3). However, we observed multiple significant differences in how the adequacy of OHS and especially IPC (overall mean of 4.67) *mitigation measures* was perceived. These apparent associations, observed to be present for all the explanatory factors we examined, drew attention to the need to consider the adjusted effect of each independent variable through the logistic regression analysis that we subsequently conducted.

**Table 3:** Unadjusted risk acceptability and mitigation adequacy associations

Explanatory variable	Risk acceptability				Mitigation adequacy			
	Work environment		Work organisation		IPC		OHS	
	Mean	P value	Mean	P value	Mean	P value	Mean	P value
Total								
By country means	4.23		4.29		4.67		6.08	
By individuals	3.88		3.87		4.79		6.28	
Region								
AFRO	4.11	0.34	4.17	0.30	**3.68**	<0.01*	**5.31**	0.03*
EMRO	4.01		4.25		**5.02**		**6.33**	
EURO	4.47		4.24		**5.28**		**6.54**	
PAHO	4.03		3.99		**4.24**		**5.92**	
SEARO	3.44		4.76		**5.11**		**6.30**	
WPRO	4.83		5.03		**5.24**		**6.35**	
Economic classification								
High	4.51	0.24	4.62	0.15	**5.61**	<0.01*	**6.99**	<0.01*
Upper middle	4.05		4.05		**4.85**		**6.17**	
Lower middle	3.78		4.05		**3.58**		**5.15**	
Low	4.51		4.27		**3.88**		**5.29**	
Gini coefficient								
Q1 (lowest)	4.80	0.11	4.51	0.34	**5.26**	0.01*	**6.64**	**0.04**
Q2	4.10		4.29		**4.31**		**5.81**	
Q3	3.90		4.04		**4.72**		**6.20**	
Q4	3.98		3.80		**3.89**		**5.55**	
COVID-19 incidence rate								
Q1 (lowest)	3.95	0.50	4.09	0.84	**4.17**	<0.01*	5.64	0.07
Q2	4.18		4.25		**4.39**		5.95	
Q3	4.50		4.39		**4.66**		6.10	
Q4	4.19		4.16		**5.44**		6.62	

Full listing of WHO region countries at https://en.wikipedia.org/wiki/List_of_WHO_regions

*Indicates statistical significance (p<0.05) of differences among the means of country mean values for category; significant values in bold.

AFRO, Africa; EMRO, Eastern Mediterranean; EURO, Europe; IPC, infection prevention and control; OHS, occupational health and safety; PAHO, Americas; Q, quartile; SEARO, South-East Asian; WPRO, Western Pacific.

To understand potential sources of difference that could be attributed to heterogeneous composition of country responses that is encountered in conducting a cross-country comparison such as the one we conducted, table 4 presents a summary of the survey’s individual-level data to indicate how gender and occupation were associated with respondent perceptions of acceptability and adequacy. Females were somewhat more likely than males to report workplace risks being unacceptable (3.76 vs 4.11; p<0.001), but the strong presence of front-line patient care providers in the gendered health workforce was largely responsible for this, as no statistically significant differences were observed within other occupation groupings (see online supplemental table S3). In fact, patient care providers themselves stood out as being the occupational grouping most critical of workplace risk acceptability as well as OHS and IPC measure adequacy. In contrast, male administrators/managers stood out as the most likely to indicate that acceptable risk exposure and adequate risk mitigation measures were present. This discrepancy is understandable as front-line workers, and women in this occupation grouping, represent those most directly experiencing the impact of the COVID-19 pandemic. However, even in these more extreme circumstances where differences were observed, the comparative differences in mean scores (that were then aggregated in calculating country mean values) were not large. Moreover, the fact that the African region, where strongest concerns about unacceptable risk and inadequate mitigation were expressed, actually had proportionately fewer female respondents, indicates that even these regional concerns that we observed may well have been under-represented in this unadjusted analysis.

**Table 4:** Risk acceptability and mitigation adequacy associations* with gender and occupation

Explanatory variable	n†	Risk acceptability				Mitigation adequacy			
		Work environment		Work organisation		IPC		OHS	
		Mean	P value	Mean	P value	Mean	P value	Mean	P value
Gender‡									
Total	4863	3.88	**<0.01**§	3.87	0.40	4.79	0.09	6.28	0.07
Female	3220	**3.76**		3.85		4.74		6.33	
Male	1643	**4.11**		3.92		4.88		6.19	
Occupation¶									
Total	4916	3.88	**0.04**	3.87	0.10	4.79	**<0.01**§	6.28	0.19
Patient care	2792	**3.91**		3.88		**4.63**		6.27	
Specialist	1404	**3.84**		3.80		**4.90**		6.30	
Admin-manager	327	**4.14**		4.22		**5.50**		6.50	
Other	393	**3.55**		3.82		**5.03**		6.08	

*This table reports on total respondents in each category, without any consideration for different mixes of gender within different occupations, and different mixes of occupation within genderss; online supplemental table S3 provides the results with full occupation and gender breakdowns.

†Total n varies by specific factor; this column refers to n for workplace environment, where response was greatest.

‡Only respondents indicating male or female were included in exploring differences.

§Indicates statistical significance (p<0.05); significant values in bold.

¶Occupation was initially coded with finer detail but then consolidated in these composites for comparative analysis.

IPC, infection prevention and control; OHS, occupational health and safety.

### Influence of between-country and within-country income disparities

Table 5 summarises the adjusted comparative effects of income level and income distribution disparity in each country setting while taking into consideration potential influences prompted by differing COVID-19 rates in the initial phase of the pandemic. While there was no difference between *higher and lower income countries* regarding the perception of unacceptable levels of *risks* in healthcare workplaces in all settings, *within-country inequality* was associated with a mildly increased likelihood of *unacceptable levels of risk* with regard to both workplace environment (OR=0.92; p=0.012) and workplace organisational (OR=0.93; p=0.017) factors.

**Table 5:** Factors associated with perceived risk acceptability and mitigation adequacy

	Unadjusted bivariate			Adjusted multivariable model*			
Explanatory variable (organised by outcome area)	OR*	95% CI	P value	B	OR*	95% CI	P value
Acceptable WP environmental risk							
Country income level†	1.56	1.08 to 2.27	0.231	0.215	1.24	0.70 to 2.20	0.708
Gini coefficient‡	**0.91**	**0.88 to 0.94**	**0.005**§	−**0.087**	**0.92**	**0.89 to 0.95**	**0.012**§
COVID log¶	1.01	0.83 to 1.23	0.965	0.083	1.09	0.78 to 1.51	0.801
Acceptable WP organisation risk							
Country income level†	0.83	0.59 to 1.17	0.587	−0.341	0.71	0.42 to 1.21	0.52
Gini coefficient‡	**0.94**	**0.91 to 0.97**	**0.028**§	−**0.076**	**0.93**	**0.90 to 0.96**	**0.017**§
COVID log¶	0.8	0.67 to 0.97	0.243	−0.113	0.89	0.66 to 1.21	0.710
Adequate IPC mitigation							
Country income level†	**6.8**	**1.36 to 34.60**	**0.006**§	**1.889**	**6.61**	**3.68 to 11.88**	**0.001**§
Gini coefficient‡	**0.93**	**0.90 to 0.95**	**0.006**§	−**0.036**	**0.94**	**0.91 to 0.96**	**0.025**§
COVID log¶	**1.85**	**1.51 to 2.26**	**0.002**§	−0.064	0.76	0.55 to 1.03	0.373
Adequate OHS mitigation							
Country income level†	**8.91**	**5.76 to 13.80**	**<0.001**§§	**2.389**	**10.91**	**5.63 to 21.12**	**<0.001**§§
Gini coefficient‡	0.97	0.94 to 0.99	0.183	−0.009	0.99	0.96 to 1.02	0.779
COVID log¶	**0.94**	**0.91 to 0.97**	**0.028**§	0.079	1.08	0.77 to 1.52	0.816

OR expressed as Exp(B) value in logistic regression analysis. *P≤0.05; **p<0.001. B is coefficient.

Variables where statistical significance is present are shown in bold.

*ORs were calculated by assessing the likelihood (OR) of the presence of a mean score ≥5 corresponding to assessments that mitigation ‘exists and offers some protection’ or better; or level of risk is assessed as ‘risk is acceptable for a short time’ or better.

†Country income was coded as comparing ‘High and Upper-Middle Income’ countries versus ‘Low and Lower-Middle Income’ countries.

‡Gini coefficient was considered in the logistic regression analysis as a continuous variable.

§Indicates statistical significance (p<0.05); also bolded.

¶COVID-19 levels with the log value of the rate of cases per million at the beginning of the survey (taken 1 June 2020); log values to smooth very high levels while taking variation into account.

CI, Confidence Interval; IPC, infection prevention and control; OHS, occupational health and safety; WP, workplace.

As was observed in unadjusted bivariate analyses, there was much stronger divergence in perceptions of acceptable *mitigation measures* by both *country income level* and *income inequality*, with an almost sevenfold greater likelihood of IPC measures (OR=6.61; p=0.001) being considered adequate in wealthier countries, and over a 10-fold difference in adequacy of OHS measures (OR=10.91; p<0.001), despite the greater intensity of COVID-19 in wealthier countries at the time of the survey. In fact, the counterintuitive positive association that seemed to be present between intensity of COVID-19 and perceptions of adequacy disappeared in our adjusted multivariable analysis. And further to the observed unadjusted effect, higher inequality decreased the likelihood (OR=0.94; p=0.025) of deeming IPC measures to be adequate.

Analysis of the more homogeneously constituted population of female patient care provider respondents (online supplemental table S6) further revealed that this group’s more critical assessment of risk that we had documented in table 4 especially influenced perceptions of risk acceptability in settings where COVID-19 exposure had intensified. In this regard, workplace organisational factors, which included consideration of the workload being encountered, were substantially more likely to be seen as unacceptable (OR=0.44; p=0.034) by female patient care providers in countries with higher COVID-19 presence; a perception reinforced by a further (although less pronounced) effect of in-country income inequality (OR=0.95; p=0.093).

As we had observed was the case for all respondents, female care providers in higher income countries were more likely to perceive mitigation measures to be adequate (OHS OR=3.94; p=0.047 and IPC OR=11.25; p=0.004) than those in more poorly resourced settings, and this was further accompanied by an effect of within-country inequality also contributing some explanatory power (OHS OR=0.92; p=0.020).

## Discussion

High levels of concern about emerging threats to HCWs were widely published in the first year of the COVID-19 pandemic, providing extensive evidence about morbidity and mortality associated with healthcare work^33 34^ as well as effects on job satisfaction.^35^ Although meta-analyses have been conducted to synthesise such findings,^36^ our article provides one of the first worldwide examinations of contextual factors affecting the well-being of HCWs during the COVID-19 pandemic, enabling a comparative cross-country analysis. In doing so, it notably complements studies calling attention to inadequate implementation of OHS and IPC measures, for example, in South Africa,^37^ as well as a need to consider the influence of structural determinants that affect how risks are experienced in specific health worker exposure contexts.^38^ The results presented here contribute a theoretical and empirically based understanding of the importance of inequality among and within countries in this regard. This has implications for preparedness for any future pandemic outbreaks.

Our findings clearly demonstrate that there is a strong need for improvements in OHS for HCWs to protect against infectious disease transmission and to control the threat of psychosocial risks, a consideration that resonates with studies highlighting the effects on mental health of HCWs as already stressed workplaces with intensifying pressures when pandemics emerge.^39–43^ Widespread concerns about health risks identified in diverse locations such as Ethiopia, Turkey, Italy and Spain in many facets of health work^44–47^ signal a strong rationale for international collaboration in seeking effective technical and policy approaches to best protect HCWs.

Despite a common assessment of unacceptable levels of risk everywhere, our study revealed important differences in the perceived adequacy of protective measures to meet this challenge. Such results point to the need to add explicit attention to OHS measures in WHO’s call for better planning healthcare human resources^10^ as well as the updating of WHO’s *Global Plan of Action for Occupational Health*, considering what this means for HCWs in light of the COVID-19 experience.

While the case prevalence in any one single country clearly influences the intensity of possible healthcare workplace exposure as a global pandemic emerges, HCWs in all countries face the same need for proper PPE, appropriate testing and vaccines as they compete in the same markets and the same supply chains.^48 49^ While there is now appropriate attention focused on the need to address global inequities in vaccine accessibility,^50^ our study highlights other inequities that also call for greater attention. Moreover, our analysis stands out by considering how variation in protecting HCWs may be associated with the presence of contextual social and economic inequities, itself an important social determinant of health that has been prominent in global health research literature. What is of particular relevance here is the vulnerability of HCWs as ‘canaries’ in a workplace made vulnerable by the emergence of a novel infectious disease,^51^ where preparedness to meet a new challenge is critical.

While the presence of unacceptable risk was clearly identified in all countries, it was striking that the strongest concern about inadequate protection of HCWs came not from the HICs hit most intensely by the initial wave of COVID-19 in early 2020, but rather less well-resourced settings that had yet to be as strongly affected. This vividly echoes pre-COVID findings that resource-poor countries have decreased capacities for protecting HCWs^13 14^ even beyond needs for testing and contact tracing, and consistent with studies noting needs for training and PPE for HCWs.^52^ This furthermore mirrors experience in previous pandemics such as Ebola in West Africa where meaningful investments in PPE were shown to be important elements in combating the spread of disease,^53^ a matter that is now being observed with regard to COVID-19.^54^ Our finding that country income level is strongly associated with greater capacity to provide prevention and mitigation within a health system is thus not surprising.

Previous literature on the effects of income inequality within a society has however been less conclusive, at times contesting the implications of the Wilkinson’s ‘economic inequality hypothesis’. In this regard, Blázquez-Fernández and colleagues concluded that income inequality does not significantly reduce health in ‘developed’ societies^55^ and Mellor and Milyo further argued that there is little support for relation between income inequality and individual or population health after fixed division effects were included.^56^ However, when attention is paid to methodological concerns,^17^ strong evidence of the effect of economic inequality has been observed in sub-Saharan African countries.^57^ Looking beyond levels of economic indicators alone, a systematic study of ‘welfare regimes’ (ie, characterisations of policy orientations dominant in a country at a particular time) has suggested that precarious workers fare better in the context of ‘Scandinavian state’ policies.^58^ Indeed, countries that recognised COVID-19 as a work-related disease and supported workers with compensation and appropriate absence policies were reported to have reduced mental health stressors, pointing to opportunities for improving HCW well-being.^59^ However, a systematic review of the impact of political economy on health observed substantial gaps in knowledge, calling for ‘higher-quality reviews and empirical studies in this area’.^60^

Our study suggests that societies with greater national income equality may well be characterised by policies that are more protective of vulnerable populations such as HCWs, a group whose comparatively high occupational health risk is aggravated by the onset of pandemics. To better understand the pathways and iterative relationships that can explain this, case study examinations would certainly be of value. Moreover, with health worker protection so strategically important to health system functioning during such crises that threaten global health equity, countries known to be highly unequal might accordingly be deemed to be in need of even further technical assistance and attention to ensure that adequate protection is provided to HCWs at risk.

Recognising that appreciation of the contribution of HCWs soared as the COVID-19 pandemic advanced, our observations that economic inequality among and within countries is associated with the degree to which HCWs face unacceptable risk and inadequate protection signal a vital need to promote social justice for those who play such an important role in the care of populations *before* a new pandemic emerges. In light of this, from an analytical perspective, we strongly endorse the call for a new paradigm^61^ to better understand how upstream and sociopolitical factors could be ‘affecting the nature of work and employment and their impact on the health of workers, the public, and the planet’.^62^ This includes consideration of international cooperation with respect to vaccine supply, and to ensure that less wealthy countries receive technical assistance in establishing protection and mitigation programmes as well as attention to pathways sensitive to the offloading of risks to more marginalised worker populations.

### Limitations and further research needs

Cross-country comparative studies such as ours rely on a convenience sample, leading to some countries being over-represented while others were under-represented or non-existent. To address possible concerns about the influence of countries with low respondent counts, we examined this concern by conducting sensitivity analyses, summarised in online supplemental table S7, to consider possible implications, but concluded that this did not warrant a questioning of our findings. Additionally, the classification of countries purely by national income levels leads to designating some countries as high income in settings where national institutions may be minimally developed despite high levels of income earned through high-value exports such as petroleum or in settings of small populations with externally controlled tourism sectors. As such, we developed grouping strategies to allow for a consideration of national contexts where resources could be considered comparatively more or less readily available to protect health workers. Stratification by WHO region was also important because these regions, while large and often heterogenous in nature, do constitute administrative units with an important governance role to play during the emergence of global outbreaks and pandemics.

It should also be acknowledged that differing perceptions of risks and mitigation measures around the world may be influenced by different HCW training and education standards, cultural nuances and institutional expectations. For example, Senthi and colleagues observed that workers in India found a high prevalence of workers unable to identify even immediate risks in an evidently hazardous environment.^63^ Studies in the Middle East also reported gaps between actual hazards and HCW recognition.^64 65^ Ndejjo and colleagues report similar findings in Uganda and across sub-Saharan Africa.^66^

## Conclusion

This study adds to the literature on how risks become unevenly distributed, focusing here on country income level but also on within-country income inequality. As noted by Gostin *et al*,^67^ WHO has an important role in supporting LMICs with technical guidance and operational assistance, while simultaneously meeting the needs of high-income countries for information sharing, research coordination and convening authorities, despite lacking both the authority and the resources to mount a more effective response to a global emergency such as this. Our study strongly suggests that international agencies with mandates related to fair trading practices and economic aid have to step up to address the disparities that threaten the healthcare workforce, and ensure that there is sufficient resilience to retain health workers needed for broader delivery of health services. It is also a matter of social justice that they do so.

## Supplementary Material

Reviewer comments

Author's manuscript

## Data Availability

Data are available upon reasonable request. Anonymised data that underlie the results reported in this article are available on justified request to the corresponding author.
