# Supplementary material for: Impacts of economic inequality on healthcare worker safety at the onset of the COVID-19 pandemic: cross-sectional analysis of a global survey
Source: BMJ Open. 2022 Oct 5;12(10):e064804. doi: 10.1136/bmjopen-2022-064804 (PMC9534779; doi:10.1136/bmjopen-2022-064804)
Supplement: Supplementary data [file bmjopen-2022-064804supp001.pdf]

Appendices

Survey instrument

Health and safety of health workers in COVID-19
Welcome to the survey on health and safety of health workers in COVID-19
Dear colleague,

This survey aims to identify the most common occupational risks for the health and safety of health workers and the measures for their prevention in the context of the ongoing pandemic of Corona Virus Infectious Disease (COVID-19).

In this survey we are interested in the health and safety of all health workers - all people engaged in the promotion, protection or improvement of the health of the population. This includes health workers involved in direct patient care, both formal and informal, in public and private facilities, including traditional medicine, as well as other assisting and supporting staff, including administration, management, ambulance drivers, public health workers, community health workers, and others.

The survey is intended for health workers, managers, and practitioners providing services for protecting the health and safety of workers in health facilities. The results will be used to inform action at all levels for improving the protection of health and safety of health workers in the ongoing response to COVID-19.

The survey has been developed by an international group of experts convened by the World Health Organization and the International Labour Organization and should take approximately 7 minutes to complete.

Your answers are completely confidential, and the data will be processed and analyzed in a way that will not link your answers to your identity.

B. About yourself and your area of work

1. In what country do you usually work?
Standard list of all countries in the world
Other (please specify)

* 2. Your gender?
Male
Female
Other
Prefer not to answer

3. What is your primary area of work? (responses below were randomized)

Administration and clerical support
Allied health professional
Community health worker
Infection prevention and control
Management and human resources
Mental health and psychosocial support
Occupational and environmental health
Patient care (medicine, nursing, midwifery, dentistry)
Pharmacy
Public health
Support staff – cleaner, driver, food worker
Other

4. You work most of the time for: (responses below were randomized)

Academia, research
Business enterprise or farm·
Employers' association /hospital federation
Healthcare facility - hospital, primary health-care centre, isolation camp
Local community
National government agency
Other
Professional association
Social care facility (e.g. nursing home, home care)·
Sub-national (provincial, district) authority
Trade union

C. Risks for health and safety of health workers

Think about the working conditions of health workers in your country, jurisdiction or health facility - those that you are most familiar with. No workplace is without risk, but some risks are negligible, or acceptable for a short time, and some are not acceptable at all. Below are some common risks for the health and safety of health workers; we are asking you to rate the current level of these risks, <u>now during the COVID-19 pandemic.</u>

5. How would you rate the level of these risks for health workers, now? (randomized)

Questions	Risk is negligible	Risk is acceptable for a short time	Risk is not acceptable at all	Don't know/Unsure
Skin damage from personal protective equipment and/or frequent hand hygiene				
Needle-sticks and sharps injuries				
Inadequate sanitation facilities				
Insufficient access to facilities for personal hygiene, such as, shower and menstrual hygiene				
Exposure to blood, body fluids, respiratory secretions, and other potentially infectious materials				

6. How would you rate the level of these risks for health workers, now? (randomized)

Questions	Risk is negligible	Risk is acceptable for a short time	Risk is not acceptable at all	Don't know/Unsure
Back injury from manual handling of patients and heavy objects				
Hazardous chemicals, drugs, cleaning and disinfection agents				
Slips, trips, and falls				
Crowded workplace				
Thermal discomfort (cold, heat, humidity)				

7. How would you rate the level of these risks for health workers, now? (randomized)

Questions	Risk is negligible	Risk is acceptable for a short time	Risk is not acceptable at all	Don't know/Unsure
Physical violence and assaults				
Bullying or psychological harassment at the workplace				
Sexual harassment				

8. How would you rate the level of these risks for health workers, now? (randomized)

Questions	Risk is negligible	Risk is acceptable for a short time	Risk is not acceptable at all	Don't know/Unsure
Regular long working hours (more than 48 hours a week)				
Time pressure, high workload				
Shift work with night shifts				
Insufficient time-off duty to rest (less than 11 hours between shifts)				

9. How would you rate the level of these risks for health workers, now? (randomized)

Questions	Risk is negligible	Risk is acceptable for a short time	Risk is not acceptable at all	Don't know/Unsure
Skin damage from personal protective equipment and/or frequent hand hygiene				
Needle-sticks and sharps injuries				
Inadequate sanitation facilities				
Insufficient access to facilities for personal hygiene, such as, shower and menstrual hygiene				
Exposure to blood, body fluids, respiratory secretions, and other potentially infectious materials				

D. Preventive measures

There are measures for the prevention of most risks for health and safety at work, but these measures may not be fully implemented and not all workers may benefit from these measures. Think again about the working conditions of health workers in your country, jurisdiction or health facility - those that you are most familiar with. The following questions are about the preventive measures for their health and safety in the real situation, now, during the COVID-19 pandemic.

10. How would you rate the level of application of these measures in the health services according to your knowledge? (randomized)

Questions	Does not exist at all	Exists and offers some protection	Exists and offers full protection	Don't know/Unsure
Policy for infection prevention and control in the health facility				
Processes for triage of patient in place at the emergency room, including early detection and isolation of infectious patients				
Routine assessment of the risk of exposure to body substances or contaminated surfaces before any health care activity and use of appropriate measures for personal protection				
Regular environmental clean-up and disinfection				
Immunization of health workers				

11. How would you rate the level of application of these measures in the health services according to your knowledge? (randomized)

Questions	Does not exist at all	Exists and offers some protection	Exists and offers full protection	Don't know/Unsure
Reporting of incidental exposures to blood, body fluids, or respiratory secretions				
Policies in place for post-exposure prophylaxis, such as, for HIV, Hepatitis B				
Facilities for hand hygiene (hand washing and disinfection) are readily available				
Personal protective equipment, such as masks, gloves, goggles, gowns are readily available				
Training and education of workers about infection prevention and control				

12. How would you rate the level of application of these measures in health services according to your knowledge? (randomized)

Questions	Does not exist at all	Exists and offers some protection	Exists and offers full protection	Don't know/Unsure
Prevention of workplace violence and security measures				
Management of working time, rest and recuperation				
Workplace policies against bullying, psychological and sexual harassment				
Human resource management of safe staffing and workload				
Psycho-social support and counselling				

13. How would you rate the level of application of these measures in the health services according to your knowledge? (randomized)

Questions	Does not exist at all	Exists and offers some protection	Exists and offers full protection	Don't know/Unsure
Occupational safety and health policy and management system in the facility				
Regular assessment of workplace health and safety risks and controls				
Engineering controls, such as ventilation, physical barriers, safer devices				
Ergonomic workplace design and furniture				
Devices for patient handling and lifting of loads				

14. How would you rate the level of application of these measures in the health services according to your knowledge? (randomized)

Questions	Does not exist at all	Exists and offers some protection	Exists and offers full protection	Don't know/Unsure
Regular medical check-ups of health workers				
Medical first aid kits				
Consultations between management and workers regarding health and safety at work				
Training and education of workers about occupational safety and health				

Other (please specify)

Table A 1: Survey options for occupation

Group	Survey responses
Patient care/health services	Patient care (medicine, nursing, midwifery, dentistry)
	Allied health professional
	Mental health and psychosocial support
	Pharmacy
	Community health worker
Specialized support	Occupational and environmental health
	Public health
	Infection prevention and control
	Support staff – cleaner, driver, food worker
Clerical support/administration and management	Administration and clerical support
	Management and human resources

Table A 2: Comparison between participants who answered risk and mitigation questions

**Variables**		**Those who responded to risk questions (n=4977)**	**Those who responded to mitigation measure questions (n=4076)**
Countries by region	AFRO	10%	11%
	EMRO	4%	4%
	EURO	35%	36%
	PAHO	31%	31%
	SEARO	3%	3%
	WPRO	15%	16%
Economic Class	High	59%	60%
	Lower-middle	10%	10%
	Upper-middle	27%	26%
	Low	4%	4%
Sex	Male	33%	34%
	Female	65%	65%
	Other / prefer not to answer	1%	1%
Occupation	Patient care/health services	56%	58%
	Specialized support	29%	29%
	Clerical support/administration and management	7%	7%
	Other	8%	7%

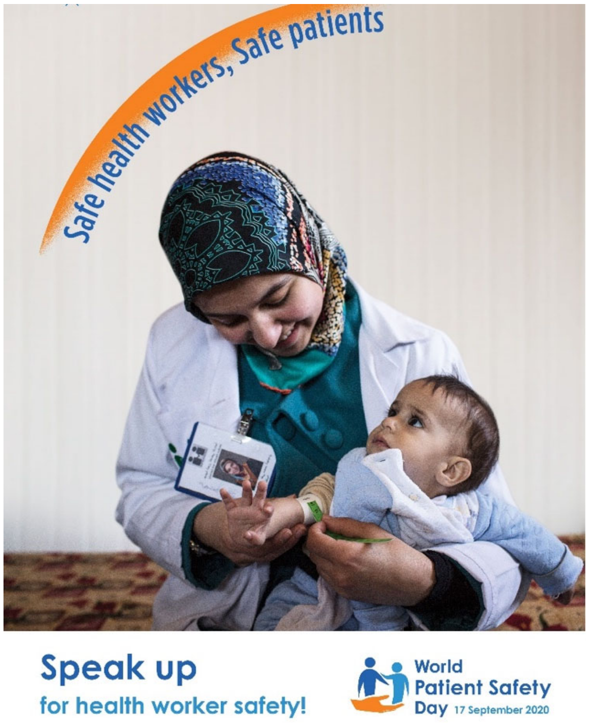

Health and safety of health workers in the context of COVID-19: A global survey

Sean P. Harrigan, Vivian W. L. Tsang, Jerry M. Spiegel, Annalee Yassi

September 15, 2020

a place of mind
THE UNIVERSITY OF BRITISH COLUMBIA
Faculty of Medicine
School of Population and Public Health
GLOBAL HEALTH RESEARCH PROGRAM
WHO Collaborating Centre for Occupational Health

© Global Health Research Program, School of Population and Public Health,

Harrigan SP, *et al. BMJ Open* 2022; 12:e064804. doi: 10.1136/bmjopen-2022-064804
