# Supplementary figures and images for: Impacts of economic inequality on healthcare worker safety at the onset of the COVID-19 pandemic: cross-sectional analysis of a global survey
Source: BMJ Open. 2022 Oct 5;12(10):e064804. doi: 10.1136/bmjopen-2022-064804 (PMC9534779; doi:10.1136/bmjopen-2022-064804)

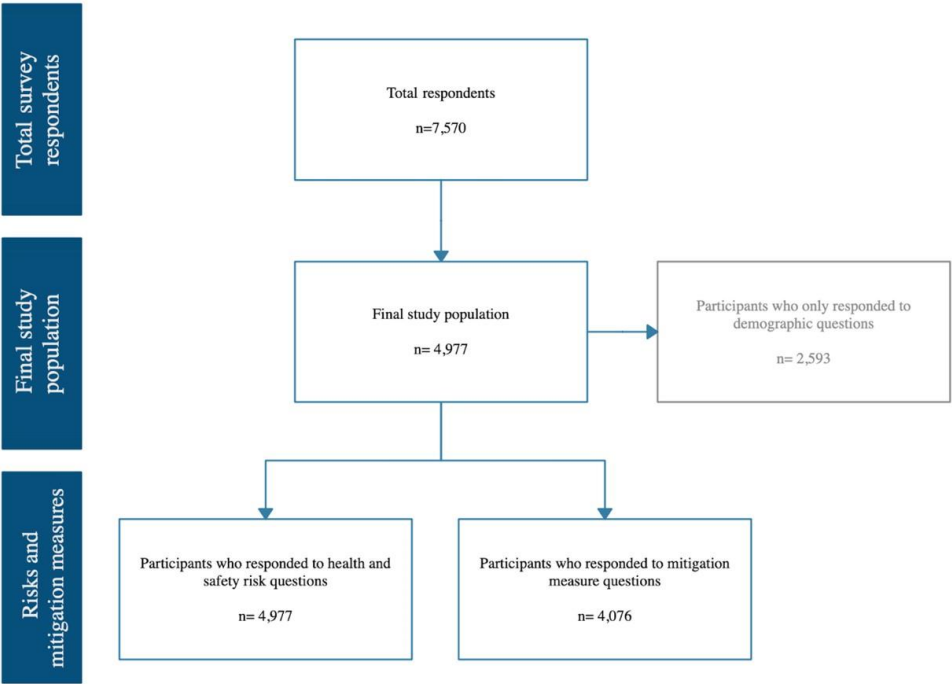

Supplement: Supplementary data [file bmjopen-2022-064804supp002.pdf]
