# Supplementary material for: Impacts of economic inequality on healthcare worker safety at the onset of the COVID-19 pandemic: cross-sectional analysis of a global survey
Source: BMJ Open. 2022 Oct 5;12(10):e064804. doi: 10.1136/bmjopen-2022-064804 (PMC9534779; doi:10.1136/bmjopen-2022-064804)
Supplement: Supplementary data [file bmjopen-2022-064804supp003.pdf]

# ONLINE SUPPLEMENTARY INFORMATION

## Table S1. Study population demographics

| Variables | | n | % |
|---|---|---|---|
| Total number of participants | | 4977 | 100% |
| Total who replied to the health and safety risks for health workers questions | | 4977 | 100% |
| Total who replied to the mitigation measures questions | | 4076 | 82% |
| Total number of countries | | 161 | 100% |
| Countries by region | African region | 516 | 10% |
| | Americas region | 1565 | 31% |
| | Eastern Mediterranean region | 221 | 4% |
| | European region | 1757 | 35% |
| | South East Asian region | 158 | 3% |
| | Western Pacific region | 760 | 15% |
| Economic Class | High | 2960 | 59% |
| | Lower-middle | 480 | 10% |
| | Upper-middle | 1324 | 27% |
| | Low | 213 | 4% |
| Sex | Male | 1654 | 33% |
| | Female | 3259 | 65% |
| | Other / prefer not to answer | 64 | 1% |
| Occupation | Patient care/health services | 2805 | 56% |
| | Specialized support | 1426 | 29% |
| | Clerical support/administration and management | 341 | 7% |
| | Other | 405 | 10% |
| Type of employer | Health services | 3038 | 61% |
| | Government services | 758 | 15% |
| | Business and farms | 473 | 10% |
| | Academia | 367 | 7% |
| | Professional associations | 177 | 4% |
| | International organization/NGO and non-for-profit | 51 | 1% |
| | Other | 113 | 2% |



Harrigan SP, *et al. BMJ Open* 2022; 12:e064804. doi: 10.1136/bmjopen-2022-064804

**Table S2. Summary of Factors and their constituent question areas**

| Survey area of focus | Factor | Description of question |
|---|---|---|
| Health and Safety Risks | Work Environment (Factor 1) | Blood and bodily fluids exposure |
| | | Skin damage from PPE and hand hygiene |
| | | Needlesticks and sharps injuries |
| | | Sanitation facilities |
| | | Personal hygiene |
| | | Thermal discomfort |
| | | Crowded workplace |
| | | Slips trips & falls |
| | | Back injury form heavy lifting |
| | | Chemicals |
| | | Bullying & harassment |
| | | Sexual harassment |
| | Work Organization (Factor 2) | Physical violence and assaults |
| | | Time pressure |
| | | Shift work |
| | | Long working hours |
| | | Insufficient rest |
| Mitigation measures | Infection protection and control (Factor 3) | IPC policy |
| | | Patient triage |
| | | Standard precautions |
| | | Cleanup & disinfection |
| | | Immunization |
| | | Reporting of blood exposure |
| | | Policies for post-exposure prophylaxis |
| | | Hand hygiene |
| | | PPE |
| | | IPC training |
| | Occupational health and safety (Factor 4) | OSH policy |
| | | Occupational health and safety risk assessment |
| | | Occupational health and safety engineering controls |
| | | Ergonomic workplace design and furniture |
| | | Safe patient handling |
| | | Violence prevention |
| | | Management of working time & rest |
| | | Policy harassment |
| | | Safe staffing & workload |
| | | Psychosocial support |
| | | Medical checkups |
| | | Medical first aid kits |
| | | Labour management consultation on OSH |
| | | Occupational health and safety training |

## Table S3: Detailed breakdowns in individual level responses by occupation and gender

### a. Detailed breakdowns in responses by occupation showing gender differences

| | | Risk acceptability | | | | Mitigation adequacy | | | |
|---|---|---|---|---|---|---|---|---|---|
| | | Work environment | | Work organization | | IPC | | OHS [f3] | |
| Population characteristic | n[c] | mean | p | mean | p | mean | p | mean | P |
| All Occupations | 4916 | **3.88** | **0.04** | 3.87 | 0.101 | **4.79** | **<0.01** | 6.28 | 0.19 |
| *m* | | ***4.11*** | ***<0.01*** | *3.92* | *0.397* | *4.88* | *0.089* | *6.19* | *0.07* |
| *f* | | ***3.76*** | | *3.85* | | *4.74* | | *6.33* | |
| - Patient Care | 2792 | **3.92** | ***0.001*** | 3.88 | *0.296* | **4.63** | *0.76* | 6.27 | *0.092* |
| *m* | | ***4.19*** | | *3.96* | | *4.65* | | *6.15* | |
| *f* | | ***3.80*** | | *3.84* | | *4.62* | | *6.32* | |
| - Specialist | 1404 | **3.84** | *0.129* | 3.80 | *0.669* | **4.90** | *0.218* | 6.30 | *0.193* |
| *m* | | *3.97* | | *3.77* | | *5.00* | | *6.20* | |
| *f* | | *3.73* | | *3.83* | | *4.81* | | *6.39* | |
| - Admin-Mgr | 327 | **4.14** | *0.06* | 4.22 | *0.632* | **5.50** | *0.512* | 6.50 | *0.872* |
| *m* | | *4.65* | | *4.33* | | *5.65* | | *6.54* | |
| *f* | | *3.92* | | *4.17* | | *5.43* | | *6.48* | |
| - Other | 393 | **3.55** | *0.205* | 3.82 | *0.189* | **5.03** | *0.20* | 6.08 | *0.89* |
| *m* | | *3.82* | | *4.10* | | *5.30* | | *6.11* | |
| *f* | | *3.43* | | *3.69* | | *4.90* | | *6.08* | |

Note: Statistical significance at p<.05 level indicated by bold; statistical significance at p<.10 level indicated by underlining.
Italic font is used for breakdown analysis (i.e. by gender) of population attribute (regular font) being analyzed (i.e. by occupation) and values are right-justified.
Regular font is used for analysis of the population attribute (regular font) being analyzed (i.e. by occupation).
Green colour indicates significant value deemed as associated with less risk (i.e. less unacceptable exposure; more adequate mitigation.
Red colour indicates statistically significant value associated with more risk (i.e. more unacceptable exposure; less adequate mitigation.



Harrigan SP, et al. BMJ Open 2022; 12:e064804. doi: 10.1136/bmjopen-2022-064804

**b. Detailed breakdowns in responses by occupation showing gender differences**

| | | Risk acceptability | | | | Mitigation adequacy | | | |
|---|---|---|---|---|---|---|---|---|---|
| | | Work environment | | Work organization | | IPC | | OHS [f3] | |
| Population characteristic | n[c] | mean | p | mean | p | mean | p | mean | P |
| Total | 4863 | **3.88** | **<0.01*** | 3.87 | 0.4 | 4.79 | 0.09 | 6.28 | 0.07 |
| *Patient Care* | | ***3.91*** | | *3.88* | | *4.63* | | *6.27* | |
| *Specialist* | | ***3.84*** | | *3.80* | | *4.90* | | *6.30* | |
| *Admin-Mgr* | | ***4.14*** | | *4.22* | | *5.50* | | *6.50* | |
| *Other* | | ***3.55*** | | *3.82* | | *5.03* | | *6.08* | |
| Female | 3220 | **3.76** | 0.189 | 3.85 | 0.273 | 4.74 | **<0.01*** | 6.33 | 0.288 |
| *Patient Care* | | *3.80* | | *3.84* | | ***4.62*** | | *6.32* | |
| *Specialist* | | *3.73* | | *3.83* | | *4.82* | | *6.39* | |
| *Admin-Mgr* | | *3.92* | | *4.17* | | ***5.43*** | | *6.48* | |
| *Other* | | *3.43* | | *3.69* | | *4.90* | | *6.07* | |
| Male | 1643 | **4.11** | 0.089 | 3.92 | 0.166 | 4.88 | **0.001** | 6.19 | 0.527 |
| *Patient Care* | | *4.19* | | *3.96* | | ***4.65*** | | *6.15* | |
| *Specialist* | | *3.97* | | *3.77* | | *5.00* | | *6.20* | |
| *Admin-Mgr* | | *4.65* | | *4.33* | | ***5.65*** | | *6.54* | |
| *Other* | | *3.82* | | *4.10* | | *5.30* | | *6.11* | |

Note: Statistical significance at p<.05 level indicated by bold; statistical significance at p<.10 level indicated by underlining.
Italic font is used for breakdown analysis (i.e. by occupation) of population attribute (regular font) being analyzed (i.e. by gender) and values are right-justified.
Regular font is used for analysis of the population attribute (regular font) being analyzed (i.e. by gender).
Green colour indicates significant value deemed as associated with less risk (i.e. less unacceptable exposure; more adequate mitigation).
Red colour indicates statistically significant value associated with more risk (i.e. more unacceptable exposure; less adequate mitigation).



Harrigan SP, *et al. BMJ Open* 2022; 12:e064804. doi: 10.1136/bmjopen-2022-064804

## Table S4. Responses to health and safety risk questions

| Question | Risk is not acceptable at all | Risk is acceptable for a short time | Risk is negligible | Don't know/unsure |
|---|---|---|---|---|
| **Infectious risks** | | | | |
| Exposure to blood, body fluids, respiratory secretions, and other potentially infectious materials | **52%** | 29% | 15% | 4% |
| Skin damage from personal protective equipment and/or frequent hand hygiene | 33% | **46%** | 16% | 5% |
| Needle-sticks and sharps injuries | **46%** | 21% | 26% | 7% |
| Inadequate sanitation facilities | **52%** | 21% | 23% | 4% |
| Insufficient access to facilities for personal hygiene, such as, shower and menstrual hygiene | **49%** | 22% | 23% | 6% |
| **Physical work environment** | | | | |
| Thermal discomfort (cold, heat, humidity) | 25% | **46%** | 24% | 5% |
| Crowded workplace | **42%** | 36% | 18% | 4% |
| Slips, trips, and falls | **34%** | 26% | 33% | 7% |
| Back injury from manual handling of patients and heavy objects | **41%** | 34% | 19% | 6% |
| Hazardous chemicals, drugs, cleaning and disinfection agents | **36%** | **36%** | 22% | 6% |
| **Psychosocial work environment** | | | | |
| Bullying or psychological harassment at the workplace | **54%** | 18% | 21% | 7% |
| Sexual harassment | **50%** | 10% | 31% | 9% |
| Physical violence and assaults | **54%** | 16% | 24% | 6% |
| **Work organization** | | | | |
| Time pressure, high workload | 38% | **49%** | 10% | 3% |
| Shift work with night shifts | 23% | **48%** | 21% | 8% |
| Regular long working hours (more than 48 hours a week) | **38%** | 42% | 15% | 5% |
| Insufficient time-off duty to rest (less than 11 hours between shifts) | **40%** | 36% | 18% | 6% |

Note: Most cited response highlighted in bold



Harrigan SP, *et al. BMJ Open* 2022; 12:e064804. doi: 10.1136/bmjopen-2022-064804

## Table S5. Responses to mitigation measure questions

| Question | Does not exist at all | Exists and offers some protection | Exists and offers full protection | Don't know/ unsure |
|---|---|---|---|---|
| **Infection prevention and control** | | | | |
| IPC policy in the health facility | 8% | **60%** | 28% | 4% |
| Patient triage | 9% | **54%** | 28% | 9% |
| Standard precautions | 15% | **53%** | 25% | 7% |
| Regular environmental clean-up and disinfection | 6% | **56%** | 34% | 4% |
| Immunization of health workers | 19% | **46%** | 26% | 9% |
| Reporting of incidental exposures to blood, body fluids, or respiratory secretions | 10% | **50%** | 32% | 8% |
| Policies in place for post-exposure prophylaxis, such as, for HIV, Hepatitis B | 8% | 41% | **42%** | 9% |
| Facilities for hand hygiene (hand washing and disinfection) are readily available | 3% | 40% | **54%** | 3% |
| Personal protective equipment, such as masks, gloves, goggles, gowns are readily available | 8% | **55%** | 34% | 3% |
| Training and education of workers about infection prevention and control | 11% | **54%** | 32% | 3% |
| **Occupational safety and health** | | | | |
| Occupational safety and health policy and management system in the facility | 14% | **58%** | 22% | 6% |
| Regular assessment of workplace health and safety risks and controls | 22% | **51%** | 21% | 6% |
| Engineering controls, such as ventilation, physical barriers, safer devices | 19% | **54%** | 19% | 8% |
| Ergonomic workplace design and furniture | 33% | **48%** | 12% | 7% |
| Devices for patient handling and lifting of loads | 27% | **48%** | 15% | 10% |
| Prevention of workplace violence and security measures | 21% | **52%** | 21% | 6% |
| Management of working time, rest and recuperation | 20% | **55%** | 19% | 6% |
| Workplace policies against bullying, psychological and sexual harassment | 27% | **43%** | 21% | 9% |
| Human resource management of safe staffing and workload | 24% | **52%** | 16% | 8% |
| Psycho-social support and counselling | 32% | **46%** | 15% | 7% |
| Regular medical check-ups of health workers | 33% | **43%** | 18% | 6% |
| Medical first aid kits | 15% | **48%** | 30% | 7% |
| Consultations between management and workers regarding health and safety at work | 25% | **50%** | 19% | 6% |
| Training and education of workers about occupational safety and health | 20% | **54%** | 21% | 5% |

Note: Most cited response highlighted in bold



Harrigan SP, *et al. BMJ Open* 2022; 12:e064804. doi: 10.1136/bmjopen-2022-064804

**Table S6: Comparing analyses of acceptability and adequacy in different stratified populations**

| | Patient Care Female | | Patient Care All | | Frontline[e] all | | All occupations | |
|---|---|---|---|---|---|---|---|---|
| **Countries included** | **112** | | **133** | | **156** | | **161** | |
| **Explanatory Variable** (organized by outcome area) | **OR[d]** | **p** | **OR[d]** | **p** | **OR[d]** | **p** | **OR[d]** | **p** |
| **Acceptable WP Enviro. Risk** | | | | | | | | |
| Country Income Level [a] | 0.81 | 0.736 | 0.63 | 0.415 | 1.01 | 0.981 | 1.24 | 0.708 |
| **Gini Coefficient** [b] | 0.98 | 0.609 | 0.96 | 0.1991 | ***0.94*** | ***0.068*** ** | **0.92** | **0.012*** |
| COVID-Log [c] | 1.12 | 0.777 | 1.33 | 0.398 | 1.22 | 0.549 | 1.09 | 0.801 |
| **Acceptable WP Org. Risk** | | | | | | | | |
| Country Income Level [a] | 1.30 | 0.657 | 1.57 | 0.446 | 1.06 | 0.926 | 0.71 | 0.52 |
| **Gini Coefficient** [b] | ***0.95*** | ***0.093*** ** | **0.93** | **0.024*** | ***0.93*** | ***0.056*** ** | **0.95** | **0.017*** |
| **COVID-Log** [c] | **0.44** | **0.034*** | 0.66 | 0.248 | 0.98 | 0.951 | 0.89 | 0.710 |
| **Adequate IPC mitigation** | | | | | | | | |
| Country Income Level [a] | **11.25** | **0.004*** | **7.48** | **0.006*** | **6.92** | **0.001*** | **6.61** | **0.001*** |
| **Gini Coefficient** [b] | 0.95 | 0.220 | 0.95 | 0.125 | **0.95** | **0.047*** | **0.94** | **0.025*** |
| COVID-Log [c] | 0.69 | 0.479 | 0.7 | 0.181 | 0.70 | 0.264 | 0.76 | 0.373 |
| **Adequate OHS mitigation** | | | | | | | | |
| Country Income Level [a] | **3.94** | **0.047*** | **5.86** | **0.004*** | **18.39** | **<0.001*** | **10.91** | **<0.001*** |
| **Gini Coefficient** [b] | **0.92** | **0.020*** | ***0.95*** | ***0.083*** ** | 0.99 | 0.755 | 0.99 | 0.779 |
| COVID-Log [c] | 0.79 | 0.556 | 0.69 | 0.281 | 0.99 | 0.988 | 1.08 | 0.816 |

[a] Country Income was coded as comparing "High and Upper-Middle Income" countries versus "Low and Lower-Middle Income" countries

[b] Gini coefficient was considered in the logistic regression analysis as a continuous variable;

[c] COVID levels where the log value of the rate of cases per million at the beginning of the survey (taken June 1, 2020); log values to smooth very high levels while taking variation into account

[d] Odds Ratios were calculated by assessing the likelihood (OR) of the presence of a mean scores ≥ 5 corresponding to assessments that mitigation "exists and offers some protection" or better; or level of risk is assessed as "risk is acceptable for a short time" or better.

[e] includes all patient care and workplace specialist support personnel such as OHS and IPC professionals

* Indicates statistical significance (p<0.05) and also bolded; ** p<.10 but >.05; indicated in italics and bold



Harrigan SP, *et al. BMJ Open* 2022; 12:e064804. doi: 10.1136/bmjopen-2022-064804

## Table S7: Comparing analyses when minimum country sample size provisions are applied

| | Patient Care Female | | Patient Care Female | | Frontline[e] | | Frontline[e] | |
|---|---|---|---|---|---|---|---|---|
| **Minimum country sample** | **2** | | **all** | | **4** | | **all** | |
| **Countries included** | **80** | | **112** | | **101** | | **161** | |
| **Explanatory Variable** (organized by outcome area) | **OR[d]** | **p** | **OR[d]** | **p** | **OR[d]** | **p** | **OR[d]** | **p** |
| **Acceptable WP Enviro. Risk** | | | | | | | | |
| Country Income Level [a] | 0.62 | 0.556 | 0.81 | 0.736 | 1.03 | 0.967 | 1.01 | 0.981 |
| **Gini Coefficient** [b] | 0.97 | 0.404 | 0.98 | 0.609 | 0.95 | 0.228 | ***0.94*** | ***0.068*** |
| COVID-Log | 1.11 | 0.852 | 1.12 | 0.777 | 0.89 | 0.797 | 1.22 | 0.549 |
| **Acceptable WP Org. Risk** | | | | | | | | |
| Country Income Level [a] | 1.2 | 0.830 | 1.30 | 0.657 | 1.33 | 0.736 | 1.06 | 0.926 |
| **Gini Coefficient** [b] | 0.97 | 0.487 | ***0.95*** | ***0.093*** | 0.94 | 0.230 | ***0.93*** | ***0.056*** |
| COVID-Log | 0.77 | 0.617 | **0.44** | **0.034*** | 0.42 | 0.457 | 0.98 | 0.951 |
| **Adequate IPC mitigation** | | | | | | | | |
| Country Income Level [a] | **9.62** | **0.041*** | **11.25** | **0.004*** | **36.89** | **0.001*** | **6.92** | **0.001*** |
| **Gini Coefficient** [b] | 0.94 | 0.223 | 0.95 | 0.220 | 0.98 | 0.647 | **0.95** | **0.047*** |
| COVID-Log | 0.54 | 0.433 | 0.69 | 0.479 | 0.48 | 0.190 | 0.70 | 0.264 |
| **Adequate OHS mitigation** | | | | | | | | |
| Country Income Level [a] | 3.00 | 0.153 | **3.94** | **0.047*** | **5.46** | **0.020*** | **18.39** | **<0.001*** |
| **Gini Coefficient** [b] | ***0.94*** | ***0.077*** | **0.92** | **0.020*** | ***0.94*** | ***0.061*** | 0.99 | 0.755 |
| COVID-Log | 0.67 | 0.392 | 0.79 | 0.556 | 0.56 | 0.152 | 0.99 | 0.988 |

[a] Country Income was coded as comparing "High and Upper-Middle Income" countries versus "Low and Lower-Middle Income" countries

[b] Gini coefficient was considered in the logistic regression analysis as a continuous variable;

[c] COVID levels where the log value of the rate of cases per million at the beginning of the survey (taken June 1, 2020); log values to smooth very high levels while taking variation into account

[d] Odds Ratios were calculated by assessing the likelihood (OR) of the presence of a mean scores ≥ 5 corresponding to assessments that mitigation "exists and offers some protection" or better; or level of risk is assessed as "risk is acceptable for a short time" or better.

[e] includes all patient care and workplace specialist support personnel such as OHS and IPC professionals

* Indicates statistical significance (p<0.05) and also bolded; ** p<.10 but >.05; indicated in italics and bold
