# Supplementary material for: Impacts of economic inequality on healthcare worker safety at the onset of the COVID-19 pandemic: cross-sectional analysis of a global survey
Source: BMJ Open. 2022 Oct 5;12(10):e064804. doi: 10.1136/bmjopen-2022-064804 (PMC9534779; doi:10.1136/bmjopen-2022-064804)
Supplement: Author's manuscript [file bmjopen-2022-064804.draft_revisions.pdf]

BMJ Open is committed to open peer review. As part of this commitment we make the peer review history of every article we publish publicly available.

When an article is published we post the peer reviewers' comments and the authors' responses online. We also post the versions of the paper that were used during peer review. These are the versions that the peer review comments apply to.

The versions of the paper that follow are the versions that were submitted during the peer review process. They are not the versions of record or the final published versions. They should not be cited or distributed as the published version of this manuscript.

BMJ Open is an open access journal and the full, final, typeset and author-corrected version of record of the manuscript is available on our site with no access controls, subscription charges or pay-per-view fees (http://bmjopen.bmj.com).

If you have any questions on BMJ Open's open peer review process please email info.bmjopen@bmj.com

BMJ Open

Impacts of economic inequality on healthcare worker safety at the onset of the COVID-19 pandemic: Cross-sectional analysis of a global survey

Journal:	*BMJ Open*
Manuscript ID	bmjopen-2022-064804
Article Type:	Original research
Date Submitted by the Author:	17-May-2022
Complete List of Authors:	Harrigan, Sean; The University of British Columbia School of Population and Public Health, Global Health Research Program<br>Tsang, Vivian; The University of British Columbia, Faculty of Medicine<br>Yassi, Annalee ; The University of British Columbia School of Population and Public Health, Global Health Research Program<br>Zungu, Muzimkhulu ; National Institute for Occupational Health; University of Pretoria Faculty of Health Sciences, School of Health Systems and Public Health<br>Spiegel, Jerry; The University of British Columbia School of Population and Public Health, Global Health Research Program
Keywords:	COVID-19, Public health < INFECTIOUS DISEASES, Health & safety < HEALTH SERVICES ADMINISTRATION & MANAGEMENT, Human resource management < HEALTH SERVICES ADMINISTRATION & MANAGEMENT, OCCUPATIONAL & INDUSTRIAL MEDICINE

SCHOLARONE™
Manuscripts

BMJ

I, the Submitting Author has the right to grant and does grant on behalf of all authors of the Work (as defined in the below author licence), an exclusive licence and/or a non-exclusive licence for contributions from authors who are: i) UK Crown employees; ii) where BMJ has agreed a CC-BY licence shall apply, and/or iii) in accordance with the terms applicable for US Federal Government officers or employees acting as part of their official duties; on a worldwide, perpetual, irrevocable, royalty-free basis to BMJ Publishing Group Ltd ("BMJ") its licensees and where the relevant Journal is co-owned by BMJ to the co-owners of the Journal, to publish the Work in this journal and any other BMJ products and to exploit all rights, as set out in our licence.

The Submitting Author accepts and understands that any supply made under these terms is made by BMJ to the Submitting Author unless you are acting as an employee on behalf of your employer or a postgraduate student of an affiliated institution which is paying any applicable article publishing charge ("APC") for Open Access articles. Where the Submitting Author wishes to make the Work available on an Open Access basis (and intends to pay the relevant APC), the terms of reuse of such Open Access shall be governed by a Creative Commons licence – details of these licences and which Creative Commons licence will apply to this Work are set out in our licence referred to above.

Other than as permitted in any relevant BMJ Author's Self Archiving Policies, I confirm this Work has not been accepted for publication elsewhere, is not being considered for publication elsewhere and does not duplicate material already published. I confirm all authors consent to publication of this Work and authorise the granting of this licence.

For peer review only

Impacts of economic inequality on healthcare worker safety at the onset of the COVID-19 pandemic: Cross-sectional analysis of a global survey

Sean P. Harrigan[1,2], Vivian W. L. Tsang[1,3], Annalee Yassi[1], Muzimkhulu Zungu[4,5], Jerry M. Spiegel[1*]

[1] Global Health Research Program, School of Population and Public Health, Faculty of Medicine, University of British Columbia, Vancouver BC, Canada

[2] Master of Science in Population and Public Health, School of Population and Public Health, Faculty of Medicine, University of British Columbia, Vancouver BC, Canada

[3] Undergraduate Medical Program, Faculty of Medicine, University of British Columbia, Vancouver BC, Canada

[4] National Institute for Occupational Health (NIOH), a division of National Health Laboratory Service (NHLS), Johannesburg, South Africa

[5] School of Health Systems and Public Health, University of Pretoria, Pretoria, South Africa

*Corresponding author

E-mail: jerry.spiegel@ubc.ca (JMS)

Jerry M. Spiegel

Professor, School of Population & Public Health, Faculty of Medicine
University of British Columbia
430-2206 East Mall, Vancouver, BC V6T 1Z3 CANADA

Word Count: 4858 words Abstract: 298 words

For peer review only

ABSTRACT

Objectives To assess the extent to which protection of healthcare workers (HCWs) as COVID-19 emerged was associated with economic inequality among and within countries.

Design Cross-sectional analysis of associations of perceptions of workplace risk acceptability and mitigation measure adequacy with indicators of respondents' respective country's economic income level (World Bank assessment) and degree of within-country inequality (Gini index).

Setting A global self-administered online survey.

Participants 4,977 HCWs and healthcare delivery stakeholders from 161 countries responded to health and safety risk questions and a subset of 4,076 (81.2%) answered mitigation measure questions. The majority (65%) of study participants were female.

Results While the levels of *risk* being experienced at the pandemic's onset were consistently deemed as unacceptable across all groupings, participants from countries with less income inequality were somewhat less likely to report unacceptable levels of risk to HCWs regarding both workplace environment (OR=0.92, p=0.012) and workplace organizational factors (OR=0.93, p=0.017) compared to counterparts in more unequal national settings. In contrast, considerable variation existed in the degree to which *mitigation* measures were considered adequate. Adjusting for other influences through a logistic regression analysis, respondents from lower-middle and low-income countries were comparatively much more likely to assess both occupational health and safety (OR=10.91, p=<0.001) and infection prevention and control [IPC] (OR=6.61, p=<0.001) protection measures as inadequate, despite much higher COVID-19 rates in wealthier countries at the time of the survey. Greater within-country income inequality was also associated with perceptions of less adequate IPC measures (OR=0.94, p=0.025). These associations remained significant when accounting for country-level differences in occupational and gender composition of respondents, including specifically when only female care providers, our study's largest and most at-risk sub-population, were examined.

Conclusions Economic inequality threatens resilience of health systems that rely on health workers working safely to provide needed care during emerging pandemics.

For peer review only - http://bmjopen.bmj.com/site/about/guidelines.xhtml

Strengths and limitations of this study

- A major strength of the study is its novel empirical testing of the "income inequality" hypothesis for a comparative cross-country analysis of a major global health challenge: protection for a workforce central to the provision of healthcare services during a pandemic.
- This study is based on a unique global self-administered online survey conducted by a network of occupational health experts coordinated by the World Health Organization (WHO) through a large array of professional networks and social media.
- A major limitation of the study is its character as a convenience sample with different compositions by gender and occupation among countries and small sample sizes in some countries; however access to gender and occupation identifiers of respondents has enabled adaptive strategies to take this into consideration.
- The study is exploratory in considering associations with economic inequality, but does not provide a way to consider pathways for this effect, so further research will be needed for this.

Keywords: COVID-19; Health Systems; Public Health; Other Study Design; Environmental Health

Occupational Health; Healthcare Workers

For peer review only

INTRODUCTION

As the COVID-19 pandemic emerged, attention was quickly drawn to risks faced by frontline healthcare providers [1,2] – and the urgent need to strengthen their protection [3,4]. By September 2020, it was estimated that 10% of global infections had been in health workers (HCWs), and over 7,000 had died [5,6]. Notwithstanding inconsistent reporting, Papoutsi and colleagues, in reviewing the global burden of COVID-19 for HCWs by country [6, 7], estimated the percentage of HCW cases among the total cases by April 2020 as ranging from less than 1% in Hong Kong and India, to 19% in Spain.

Despite 60 million people employed in the healthcare sector worldwide [8,9], a global shortage of HCWs persists and is especially critical in low-and middle-income countries (LMICs) [10], where the greatest global burden of disease exists [11]. Risk mitigation is marked by considerable variation [12], with shortcomings in infrastructure and mitigation programs contributing to higher burdens of disease and HCW risk in more poorly resourced settings [13]. The danger that HCWs face of acquiring COVID-19 adds to extensive existing risks in infectious disease endemic states, for example with tuberculosis in Sub-Saharan Africa [14].

While lack of personal protective equipment (PPE) was highlighted early in the COVID-19 pandemic, consideration of broader OHS factors and mitigation measures attracted less initial attention [7,8,15]. To ascertain the extent of OHS risk exposure and the adequacy of mitigation measures in place to meet the challenge of COVID-19, an Ad Hoc expert group of the WHO, the International Labour Organization (ILO) and the International Commission on Occupational Health (ICOH) prepared and circulated a questionnaire survey to identify HCWs perceptions of

For peer review only

the most common threats to their health and safety as well as the adequacy of mitigation measures in the emerging pandemic [16].

Further to a preliminary analysis of survey results [16] regarding risk and adequacy of protection, we sought to ascertain the degree to which perceived risk exposure of HCWs and adequacy of mitigation measures is associated with a country's economic characteristics. Considerable attention, after all, has been given to the impact of economic disparity on health [17,18], especially in relation to Wilkinson's "economic inequality hypothesis" suggesting that greater inequality is associated with poorer health [19]. In recognition that "the traditional exposure-disease framework used in occupational health research is not equipped to address societal contexts in which work is embedded" [20], we sought to examine how such driving forces [21]as a country's economic inequality might be affecting the wellbeing of HCWs .

A variety of factors have been examined that might have influenced how the onset of the COVID-19 pandemic was experienced in different national settings, including consideration of cultural traits [22], specific government regulations and non-pharmaceutical interventions [23,24], and political leadership characteristics [25]. Our study sought to apply a cross-country perspective to consider the effects of economic inequality, recognizing this to be a dimension of considerable relevance in global public health research.

With this focus, we set out to first consider variation in perceptions of the acceptability of work-related risks and the adequacy of mitigation measures that were being experienced by HCWs as COVID-19 emerged; and, second, to determine the extent to which variations were associated with a country's comparative income level and degree of income inequality.

For peer review only - http://bmjopen.bmj.com/site/about/guidelines.xhtml

METHODS

Survey development

Shortly after the *WHO Ad Hoc Study Group on Health and Safety of Health Workers* was established when the COVID-19 pandemic emerged, it created an online survey aimed at HCWs from all WHO regions globally. In addition to the capture of demographic indicators of respondents, the survey contained 41 questions – 17 on health and safety risks and 24 on mitigation measures [16, Appendix]. Risk questions were grouped into those related to infectious disease transmission, physical work environment, psychological work environment and work organization. For each risk question, participants were asked *"Think about the working conditions of health workers in your country, jurisdiction or health facility….; rate the current level of these risks, now during the COVID-19 pandemic*." Questions regarding mitigation measures were divided into two groups: occupational health and safety (OHS) and infection prevention and control (IPC). Here, participants were similarly asked: *"Think about the working conditions of health workers in your country, jurisdiction or health facility…rate the level of application of these measures according to your knowledge of the real situation now during the COVID-19 pandemic."*

Patient and Public Involvement

The participation of health workers (whose wellbeing is the focus for this study in relation to their assessment of the adequacy of measures to protect them) was indirectly included through the participation of their representatives (unions within the ILO and other professional bodies) who were directly involved in the creation of the research instrument and in the dissemination of the online survey and its initial results.

For peer review only

For peer review only - http://bmjopen.bmj.com/site/about/guidelines.xhtml

Study population and inclusion criteria

Participants were recruited by convenience sampling, with dissemination through a large array of professional networks and social media. The survey, self-administered online to enable rapid low-cost recruitment, was available in Arabic, Chinese, English, French, German, Italian, Portuguese, Russian, Spanish and Swahili. A range of HCWs and stakeholders involved with healthcare delivery were invited to participate. In addition to HCWs in direct patient care in both formal and informal settings and in public and private facilities, respondents also included allied health and supporting staff, including OHS and IPC professionals, administration, management, drivers, public health workers, community health workers, and others as defined by the International Standard Classification of Occupations (ISCO-08). Data collection occurred between May 5th and June 25th, 2020. Participant results were excluded if they failed to complete demographic questions or if they failed to provide any responses to the risk and mitigation questions. As the survey was designed to be completed and submitted anonymously, no formal request for signed consent was solicited, with participants' submission itself indicating consent to use the information provided as anonymized aggregated data. The study proposal was approved by the Behavioural Research Ethics Board of the University of British Columbia (Ref. H20-01825). This work was supported by the International Development Research Centre (IDRC) under grant M20-00559 and the Canadian Institutes of Health Research (CIHR) under grant VS1-175519 for the "Protecting healthcare workers from COVID-19: a comparative contextualized analysis" research programme.

Independent variables

Demographic information for individual survey respondents was collected on country, gender, and occupation – the latter separated into 13 categories and then grouped into patient care/health services; specialized technical support; clerical support/administration and management; and other. Details about the study sample population composition and demographic characteristics of participants are presented in Figure S1 and Table S1 respectively.

Our research group, drawn from two WHO Collaborating Centres participating in the survey process, conducted the analysis by consolidating respondents by their home country and then linking this to a WHO geographic region [26]; a comparative country-level economic classification by World Bank income groups based on the annual Atlas gross national income per capita estimates [26,27]; and the country's Gini index - a measure used for the analysis of income inequality present within a country, with a score of 0 representing perfect equality, and a score of 1 representing complete inequality [28]. Data for Gini and economic classification were taken from the World Bank, using the most recent data available. To take account of the variation across regions present during the initial phase of the pandemic, we also considered COVID-19 incidence per million (logarithmic scale) in each country at the time when the survey was completed, as an indicator of the intensity as of a particular date, using values for June 2020 drawn from the "Our World in Data" database [29].

Dependent variables

Acceptability of workplace risks and adequacy of mitigation measures – the dependent variables in this study – were derived from a factor analysis of individual survey responses, then

For peer review only

aggregated to enable subsequent analysis of the effect of country-level characteristics. Factor analysis [16] was used to reduce the 41 survey questions into coherent groupings and principal component analysis with varimax rotation carried out to create factors from each set of workplace health and safety risk exposure questions (corresponding to workplace risk and workplace organization acceptability) and mitigation measure questions (corresponding to IPC and OHS adequacy); Table S2 summarizes the subject matter covered by the questions consolidated in each factor. Separate factor analyses were run on risk questions and preventive measure questions. Missing values were excluded in a listwise fashion. The rotated component matrix was used to identify factors. To measure scale reliability, Cronbach's Alpha was used for each individual factor. Scores over 0.7 are considered to be acceptable for internal consistency [30]. The results from the factor analysis are outlined further in our preliminary analysis [16].

The questions were administered as a 3-point Likert scale, then converted to a 10-point scale for clearer communication (i.e. midpoint of 2 becoming 5). Numerical scores were assigned to each answer to establish a scale for both the risk and mitigation measure factors, with higher scores corresponding to more desirable states. For health and safety risks, a score of 0 was assigned to "risk is not acceptable at all"; 5 to "risk is acceptable for a short time"; and 10 for "risk is negligible". For mitigation measures, a score of 0 was assigned to "does not exist at all"; 5 to "exists and offers some protection"; and 10 to "exists and offers full protection". Responses of "don't know/unsure" were assigned blanks. Factor scores were then calculated to form an individual respondent's factor score for each of the four groupings, i.e., work environment risk acceptability, work organization risk acceptability, OHS adequacy and IPC adequacy and then aggregated to generate a mean value for each country's respondents, so that inter-country

For peer review only - http://bmjopen.bmj.com/site/about/guidelines.xhtml

comparison could be conducted. The higher the scores, the greater the perceived adequacy of mitigation measures or acceptability of risk deemed as being experienced.

Analysis

The mean country dependent variable factor scores derived from the aggregation of individual participants' responses served as the basis for considering associations by WHO region, economic classification, Gini coefficients and COVID-19 incidence. Comparisons of survey mean scores were carried out using ANOVA analysis, with an alpha of 0.05 used to test significance. To compare means for the continuous variable Gini coefficient and COVID incidence scores, we ordinally divided groups of countries into quartiles by values.

To ensure that intercountry variation was not purely explained by possible gender and occupational compositional differences among a particular country's respondents, we carefully examined possible sources of discrepancy (Table S3), using ANOVA analysis to consider effects that could complicate the cross-country comparison of all respondents. To minimize any such effect, we considered different ways to stratify our analysis of the study population, notably by focusing only on those populations that had the most direct workplace experience to personally being "at risk". Noting the presence of gender differences among patient care deliverers, we specifically isolated female respondents, who in fact constituted the largest demographic group of respondents in the study, representing 1,998 respondents from 112 countries (n=1,968 from 112 countries), the largest sub-population.

For peer review only - http://bmjopen.bmj.com/site/about/guidelines.xhtml

Finally, to measure the effect that the interaction of independent variables had on the likelihood of workplace risks being considered as acceptable in a country setting as COVID was emerging, and workplace protection and control measures being deemed as adequate, we created and applied a logistic regression model. Preferred outcomes for this analysis were assessed as mean factor scores ≥ 5, corresponding to assessments that mitigation "exists and offers some protection" or better; or "risk is acceptable for a short time" or better. All statistical analysis was done using R and SPSS Statistics software [31,32].

RESULTS

Overall study population and survey responses

There were 4,977 participants who responded to health and safety risk questions and a subset of 4,076 (81.2%) who answered mitigation measure questions. The majority of study participants were female (65%), reflecting the make-up of the health sector workforce. Most participants were from the European region (35%), followed by the Americas (31%), the Western Pacific region (15%) and Africa (10%); the South East Asian (4%) and Eastern Mediterranean regions (3%) made up the smallest proportion of participants. In total there were 161 countries represented in the survey. Portugal (n=549, 11%), US (n=451, 9%), Brazil (n=373, 7%), Canada (n=263, 5%), and China (n=233, 5%) had the most participants. The majority of respondents were from countries of high-economic classification (59%), followed by upper-middle (27%), lower middle (10%) and low (4%). Most survey participants worked for a health services employer (61%), followed by government services (15%) and businesses and farms (10%). Those working in academia, professional associations, international organizations and non-government organizations each encompassed less than 10%. Finally, the type of occupation was predominantly patient care/health (56%) services, followed by 29% providing technical services

For peer review only

such as IPC or OHS specialists, 7% in administration and 10% identified as working in other sectors (Table S1).

The largest percentage of countries were in Europe (30%) and over a third of all countries were high-income countries (35%). The average Gini index was 37.8 (SD=7.7) and the mean and median COVID-19 incidence rate per million was 1,360 and 278, respectively at the time the survey was conducted.

Table 1 illustrates that considerable variation exists in these variables across the different WHO regions, indicating the distinct characteristics and conditions present at the onset of the pandemic. It is especially noteworthy that case levels had been far greater in high-income country areas at the survey mid-point (June 1st, 2020). For example, the cases per million was 2,525 in Europe versus 119 in Africa; 5,408 in the United States; and only 138 in India and 97 in Indonesia.

Table 1: Country characteristics of different WHO regions

Region	Number of countries	Countries by income classification*				Mean country values		Study Population characteristics	
		High	Upper-middle	Lower-middle	Low	Inequality: Gini coefficient*	COVID: cases per million a,*	Gender: Female* (%)	Occupation: Frontline Patient care* (%)
Overall	161	57	42	36	26	37.8	1,360	65.5%	56.4%
AFRO	37	0	6	12	19	43.2	119	44.8%	52.1%
EMRO	20	6	3	7	4	35.2	2,407	39.8%	29.4%
EURO	48	32	13	2	1	31.8	2,525	68.2%	64.4%
PAHO	30	11	14	4	1	44.8	1,135	73.3%	46.5%
SEARO	9	0	2	6	1	35.0	86	36.7%	56.3%
WPRO	17	8	4	5	0	37.0	512	70.7%	68.8%

Abbreviations: AFRO: Africa; EMRO: Eastern Mediterranean; EURO: Europe; p: p-values; PAHO: Americas; SEARO: South-East Asian; WPRO: Western Pacific
Note: * p <0.001, a: COVID rates as of June 2020.

For peer review only - http://bmjopen.bmj.com/site/about/guidelines.xhtml

As summarized in Table 2 (full table in appendix Table S4), the majority of respondents designated most of the health and safety risk parameters as "not acceptable at all". Circumstances most reported as such included bullying or psychological harassment in the workplace (54%), physical violence and assaults (54%), exposure to blood, bodily fluids, and other infectious materials (52%), inadequate sanitation facilities (52%), and sexual harassment (50%). In contrast, areas such as time pressure and high workload (38%), skin damage from PPE (33%) and shift work with night shifts (23%) were deemed to be less of a concern. There were no risk categories in which the most common response was "risk is negligible".

Mitigation measures related to the above areas of concern were seen as particularly lacking, with only the category of "policies for facilities for hand hygiene" designated as "exists and offers full protection" (full tables in appendix Table S5). For example, despite psychosocial-related risks, including bullying, harassment, physical violence, and sexual harassment ranked consistently high (54%, 54%, 50% respectively), only 21% indicated that corresponding policies "exist and offer full protection", with similar dissatisfaction for the adequacy of mitigation measures for other key areas such as IPC policy (28%), availability of PPE (34%) as well as training and education of workers about OHS (21%) and IPC (32%). Only in two mitigation measures areas – availability of facilities for hand hygiene, and policies for post-exposure prophylaxis (such as HIV or hepatitis B) – did most participants indicate that measures existed and offered full protection (54% and 42%, respectively). These results show an overwhelming majority of participants indicating that the risks they faced were not acceptable at all and that very few of the corresponding mitigation measures offered adequate protection to HCWs.

For peer review only - http://bmjopen.bmj.com/site/about/guidelines.xhtml

Table 2: Risk acceptability and mitigation adequacy – selected worldwide survey responses

Risk acceptability	Risk is not acceptable at all	Risk is acceptable for a short time	Risk is negligible	Don't know/unsure
**Infectious risk work environment**				
Exposure to blood, body fluids, respiratory secretions, other potentially infectious materials	**52%**	29%	15%	4%
Inadequate sanitation facilities	**52%**	21%	23%	4%
Skin damage from personal protective equipment and/or frequent hand hygiene	33%	**46%**	16%	5%
**Physical work environment**				
Crowded workplace	**42%**	36%	18%	4%
Thermal discomfort (cold, heat, humidity)	25%	**46%**	24%	5%
**Psychosocial work environment**				
Bullying or psychological harassment	**54%**	18%	21%	7%
Sexual harassment	**50%**	10%	31%	9%
**Work organization**				
Time pressure, high workload	38%	**49%**	10%	3%
Shift work with night shifts	23%	**48%**	21%	8%

Mitigation measure adequacy	Does not exist at all	Exists and offers some protection	Exists and offers full protection	Don't know/ unsure
**Infection prevention and control**				
IPC policy in the health facility	8%	**60%**	28%	4%
Personal protective equipment, e.g. masks, gloves, goggles, gowns are readily available	8%	**55%**	34%	3%
Training and education of workers about infection prevention and control	11%	**54%**	32%	3%
Facilities for hand hygiene (hand washing and disinfection) are readily available	3%	40%	**54%**	3%
**Occupational health and safety**				
Occupational health and safety policy and management system in the facility	14%	**58%**	22%	6%
Regular assessment of workplace health and safety risks and controls	22%	**51%**	21%	6%
Engineering controls, such as ventilation, physical barriers, safer devices	19%	**54%**	19%	8%
Prevention of workplace violence and security measures	21%	**52%**	21%	6%
Workplace policies against bullying, psychological and sexual harassment	27%	**43%**	21%	9%

Note: Most cited response highlighted in bold

For peer review only

Associations with risk exposure acceptability and mitigation measure adequacy

Unacceptable levels of *risk* (i.e., factor scores below 5) were consistently reported for both Work Organization and Work Environment across geographic regions, economic income level categories, equity classifications and COVID-19 incidence rates, with no statistically significant differences observed within these categories (Table 3). However, we observed multiple significant differences in how the adequacy of OHS and especially IPC (overall mean of 4.67) *mitigation measures* were perceived. These apparent associations, observed to be present for all the explanatory factors we examined, drew attention to the need to consider the adjusted effect of each independent variable through the logistic regression analysis that we subsequently conducted.

For peer review only

For peer review only - http://bmjopen.bmj.com/site/about/guidelines.xhtml

Table 3:Unadjusted risk acceptability and Mitigation adequacy associations

		Risk acceptability				Mitigation adequacy			
		Work environment		Work organization		IPC		OHS	
**Explanatory Variable**		**mean**	**p**	**mean**	**p**	**mean**	**p**	**mean**	**P**
**TOTAL**	By country means	4.23		4.29		4.67		6.08	
	By individuals	3.88		3.87		4.79		6.28	
**Region**	AFRO	4.11	0.34	4.17	0.30	**3.68**	**<0.01***	**5.31**	**0.03***
	EMRO	4.01		4.25		**5.02**		**6.33**	
	EURO	4.47		4.24		**5.28**		**6.54**	
	PAHO	4.03		3.99		**4.24**		**5.92**	
	SEARO	3.44		4.76		**5.11**		**6.30**	
	WPRO	4.83		5.03		**5.24**		**6.35**	
**Economic Classification**	High	4.51	0.24	4.62	0.15	**5.61**	**<0.01***	**6.99**	**<0.01***
	Upper-middle	4.05		4.05		**4.85**		**6.17**	
	Lower-middle	3.78		4.05		**3.58**		**5.15**	
	Low	4.51		4.27		**3.88**		**5.29**	
**Gini coefficient**	Q1 [lowest]	4.80	0.11	4.51	0.34	**5.26**	**0.01***	**6.64**	**0.04**
	Q2	4.10		4.29		**4.31**		**5.81**	
	Q3	3.90		4.04		**4.72**		**6.20**	
	Q4	3.98		3.80		**3.89**		**5.55**	
**COVID-19 incidence rate**	Q1 [lowest]	3.95	0.50	4.09	0.84	**4.17**	**<0.01***	5.64	0.07
	Q2	4.18		4.25		**4.39**		5.95	
	Q3	4.50		4.39		**4.66**		6.10	
	Q4	4.19		4.16		**5.44**		6.62	

Abbreviations: AFRO: Africa; EMRO: Eastern Mediterranean; EURO: Europe; IPC: Infection protection and control; OHS: Occupational health and safety; p: p-values; PAHO: Americas; SEARO: South-East Asian; Q: Quartile; WPRO: Western Pacific

* Indicates statistical significance (p<0.05) of differences among the means of country mean values for category; significant values in bold

For peer review only

To understand potential sources of difference that could be attributed to heterogeneous composition of country responses that is encountered in conducting a cross-country comparison such as the one we conducted, Table 4 presents a summary of the survey's individual level data to indicate how gender and occupation were associated with respondent perceptions of acceptability and adequacy. Females were somewhat more likely than males to report workplace risks being unacceptable (3.76 versus 4.11; $p<0.001$), but the strong presence of frontline patient care providers in the gendered health workforce was largely responsible for this, as no statistically significant differences were observed within other occupation groupings (see Table S3). In fact, patient care providers themselves stood out as being the occupational grouping most critical of workplace risk acceptability as well as OHS and IPC measure adequacy. In contrast, male administrators/managers stood out as the most likely to indicate that acceptable risk exposure and adequate risk mitigation measures were present. This discrepancy is understandable as frontline workers, and women in this occupation grouping, represent those most directly experiencing the impact of the COVID pandemic. However, even in these more extreme circumstances where differences were observed, the comparative differences in mean scores (that were then aggregated in calculating country mean values) were not large. Moreover, the fact that the African region, where strongest concerns about unacceptable risk and inadequate mitigation were expressed, actually had proportionately fewer female respondents, indicates that even these regional concerns that we observed may well have been underrepresented in this unadjusted analysis.

For peer review only - http://bmjopen.bmj.com/site/about/guidelines.xhtml

Table 4: Risk acceptability and mitigation adequacy associations [a] with gender and occupation

			Risk acceptability				Mitigation adequacy			
			Work environment		Work organization		IPC		OHS	
Explanatory Variable		n[d]	mean	p	mean	P	mean	p	mean	P
**Gender[b]**	Total	4863	3.88	**<0.01***	3.87	0.40	4.79	0.09	6.28	0.07
	Female	3220	**3.76**		3.85		4.74		6.33	
	Male	1643	**4.11**		3.92		4.88		6.19	
**Occupation[c]**	Total	4916	3.88	**0.04**	3.87	0.10	4.79	**<0.01***	6.28	0.19
	Patient Care	2792	**3.91**		3.88		**4.63**		6.27	
	Specialist	1404	**3.84**		3.80		**4.90**		6.30	
	Admin-Mgr	327	**4.14**		4.22		**5.50**		6.50	
	Other	393	**3.55**		3.82		**5.03**		6.08	

* Indicates statistical significance ($p<0.05$); significant values in bold

[a] This table report on total respondents in each category, without any consideration for different mixes of gender within different occupations, and different mixes of occupation within genders; Supplementary table X provides results with full occupation and gender breakdowns

[b] Only respondents indicating Male or Female were included in exploring differences;

[c] Occupation was initially coded with finer detail but then consolidated in these composites for comparative analysis

[d] total n varies by specific factor; this column refers to n for workplace environment, where response was greatest

Abbreviations: IPC: Infection protection and control; Mgr: Manager ; OHS: Occupational health and safety

Influence of between-country and within-country income disparity

Table 5 summarizes the adjusted comparative effects of income level and income distribution disparity in each country setting while taking into consideration potential influences prompted by differing COVID-19 rates in the initial phase of the pandemic. While there was no difference between *higher and lower income countries* regarding the perception of unacceptable levels of *risks* in healthcare workplaces in all settings, *within-country inequality* was associated with a mildly increased likelihood of *unacceptable levels of risk* with regard to both workplace environment (OR=0.92; p=0.012) and workplace organizational (OR=0.93; p=0.017) factors.

For peer review only - http://bmjopen.bmj.com/site/about/guidelines.xhtml

Table 5: Factors associated with perceived risk acceptability and mitigation adequacy

Explanatory Variable (organized by outcome area)	Unadjusted bivariate			Adjusted multivariable model [d]			
	OR[d]	95% CI	p	B	OR[d]	95% CI	P
**Acceptable WP Enviro. Risk**							
Country Income Level [a]	1.56	1.08-2.27	0.231	0.215	1.24	0.70-2.20	0.708
**Gini Coefficient [b]**	**0.91**	**0.88-0.94**	**0.005***	**-0.087**	**0.92**	**0.89-0.95**	**0.012***
COVID-Log[c]	1.01	0.83-1.23	0.965	0.083	1.09	0.78-1.51	0.801
**Acceptable WP Org. Risk**							
Country Income Level [a]	0.83	0.59-1.17	0.587	-0.341	0.71	0.42-1.21	0.52
**Gini Coefficient [b]**	**0.94**	**0.91-0.97**	**0.028***	**-0.076**	**0.93**	**0.90-0.96**	**0.017***
COVID-Log[c]	0.8	0.67-0.97	0.243	-0.113	0.89	0.66-1.21	0.710
**Adequate IPC mitigation**							
**Country Income Level [a]**	**6.8**	**1.36-34.60**	**0.006***	**1.889**	**6.61**	**3.68-11.88**	**0.001***
**Gini Coefficient [b]**	**0.93**	**0.90-0.95**	**0.006***	**-0.036**	**0.94**	**0.91-0.96**	**0.025***
**COVID-Log[c]**	**1.85**	**1.51-2.26**	**0.002***	-0.064	0.76	0.55-1.03	0.373
**Adequate OHS mitigation**							
**Country Income Level [a]**	**8.91**	**5.76-13.80**	**<0.001***	**2.389**	**10.91**	**5.63-21.12**	**<0.001***
Gini Coefficient [b]	0.97	0.94-0.99	0.183	-0.009	0.99	0.96-1.02	0.779
**COVID-Log[c]**	**0.94**	**0.91-0.97**	**0.028***	0.079	1.08	0.77-1.52	0.816

Notes: OR: Odds Ratio, expressed as Exp(B) value in Logistic Regression analysis, CI: Confidence Interval, * p ≤ .05 ** <001
Abbreviations: B: Coefficient; OHS: Occupational Health and Safety mitigation; Org.: organization; IPC: Infection Prevention and control; Enviro.: environmental; WP: workplace
Variables where statistical significance is present are shown in bold
[a] Country Income was coded as comparing "High and Upper-Middle Income" countries versus "Low and Lower-Middle Income" countries
[b] Gini coefficient was considered in the logistic regression analysis as a continuous variable;
[c] COVID levels where the log value of the rate of cases per million at the beginning of the survey (taken June 1, 2020); log values to smooth very high levels while taking variation into account
[d] Odds Ratios were calculated by assessing the likelihood (OR) of the presence of a mean score ≥ 5 corresponding to assessments that mitigation "exists and offers some protection" or better; or level of risk is assessed as "risk is acceptable for a short time" or better.
* Indicates statistical significance (p<0.05); also bolded

As was observed in unadjusted bivariate analyses, there was much stronger divergence in perceptions of acceptable *mitigation measures* by both *country income level* and *income inequality*, with an almost 7-fold greater likelihood of IPC measures (OR=6.61; p=0.001) being considered adequate in wealthier countries, and over a ten-fold difference in adequacy of OHS

For peer review only - http://bmjopen.bmj.com/site/about/guidelines.xhtml

measures (OR=10.91; p<0.001), despite the greater intensity of COVID-19 in wealthier countries at the time of the survey. In fact, the counter-intuitive positive association that seemed to be present between intensity of COVID-19 and perceptions of adequacy disappeared in our adjusted multivariable analysis. And further to the observed unadjusted effect, higher inequality decreased the likelihood (OR=0.94; p=0.025) of deeming IPC measures to be adequate.

Analysis of the more homogeneously constituted population of female patient care provider respondents (Table S6) further revealed that this group's more critical assessment of risk that we had documented in Table 4 especially influenced perceptions of risk acceptability in settings where COVID-19 exposure had intensified. In this regard, workplace organizational factors, which included consideration of the workload being encountered, were substantially more likely to be seen as unacceptable (OR=0.44; p=0.034) by female patient care providers in countries with higher COVID presence; a perception reinforced by a further (albeit less pronounced) effect of in-country income inequality (OR=0.95; p=0.093).

As we had observed was the case for all respondents, female care providers in higher income countries were more likely to perceive mitigation measures to be adequate (OHS OR=3.94; p=0.047 and IPC OR=11.25; p=0.004) than those in more poorly resourced settings, and this was further accompanied by an effect of within-country inequality also contributing some explanatory power (OHS OR=0.92; p=0.020).

For peer review only

DISCUSSION

High levels of concern about emerging threats to HCWs were widely published in the first year of the COVID-19 pandemic, providing extensive evidence about morbidity and mortality associated with healthcare work [33–35] as well as effects on job satisfaction [36]. Although meta-analyses have been conducted to synthesize such findings [37], our article provides one of the first worldwide examinations of contextual factors affecting the wellbeing of HCWs during the COVID-19 pandemic, enabling a comparative cross-country analysis. In doing so, it notably complements studies calling attention to inadequate implementation of OHS and IPC measures, for example in South Africa [38] as well as a need to consider the influence of structural determinants that affect how risks are experienced in specific health worker exposure contexts [39]. The results presented here contribute a theoretical and empirically-based understanding of the importance of inequality among and within countries in this regard. This has implications for preparedness for any future pandemic outbreaks.

Our findings clearly demonstrate that there is a strong need for improvements in OHS for HCWs not only to protect against infectious disease transmission but to also control the threat of psychosocial risks, a consideration that resonates with studies highlighting effects on mental health of HCWs as already stressed workplaces with intensifying pressures when pandemics emerge [40–44]. Widespread concerns about health risks identified in diverse locations such as Ethiopia, Turkey, Italy and Spain in many facets of health work [45–48] signal a strong rationale for international collaboration in seeking effective technical and policy approaches to best protect HCWs.

For peer review only - http://bmjopen.bmj.com/site/about/guidelines.xhtml

Despite a common assessment of unacceptable levels of risk everywhere, our study revealed important differences in the perceived adequacy of protective measures to meet this challenge. Such results point to the need to add explicit attention to OHS measures in the World Health Organization's call for better planning healthcare human resources [10] as well as the updating of the WHO's *Global Plan of Action for Occupational Health*, considering what this means for HCWs in light of the COVID-19 experience.

While the case prevalence in any one single country clearly influences the intensity of possible healthcare workplace exposure as a global pandemic emerges, HCWs in all countries face the same need for proper PPE, appropriate testing and vaccines as they compete in the same markets and the same supply chains [49,50]. While there is now appropriate attention focused on the need to address global inequities in vaccine accessibility [51], our study highlights other inequities that also need greater attention. Moreover, our analysis stands out by considering how variation in protecting HCWs may be associated with the presence of contextual social and economic inequities, itself an important social determinant of health that has been prominent in global health research literature. What is of particular relevance here is the vulnerability of HCWs as "canaries" in a workplace made vulnerable by the emergence of a novel infectious disease [52], where preparedness to meet a new challenge is critical.

While the presence of unacceptable risk was clearly identified in all countries, it was striking that the strongest concern about inadequate protection of HCWs came not from the HICs hit most intensely by the initial wave of COVID in early 2020, but rather less well-resourced settings that had yet to be as strongly affected. This vividly echoes pre-COVID findings that resource-poor

For peer review only - http://bmjopen.bmj.com/site/about/guidelines.xhtml

countries have decreased capacities for protecting HCWs [13,14] even beyond needs for testing and contact tracing, and consistent with studies noting needs for training and PPE for HCWs [53]. This furthermore mirrors experience in previous pandemics such as Ebola in West Africa where meaningful investments in PPE were shown to be important elements in combatting the spread of disease [54], a matter that is now being observed with regard to COVID-19 [55]. Our finding that country income level is strongly associated with greater capacity to provide prevention and mitigation within a health system is thus not surprising.

Previous literature on the effects of income inequality within a society has however been less conclusive, at times contesting the implications of the Wilkinson's "economic inequality hypothesis". In this regard, Blázquez-Fernández and colleagues concluded that income inequality does not significantly reduce health in 'developed' societies [56] and Mellor and Milyo further argued that there is little support for relation between income inequality and individual or population health after fixed division effects were included [57]. However, when attention is paid to methodological concerns [17], strong evidence of the effect of economic inequality has been observed in Sub-Saharan African countries [58]. Looking beyond levels of economic indicators alone, a systematic study of "welfare regimes" (i.e. characterizations of policy orientations dominant in a country at a particular time) has suggested that precarious workers fare better in the context of "Scandinavian state" policies [59]. Indeed countries that recognized COVID-19 as a work-related disease and supported workers with compensation and appropriate absence policies, were reported to have reduced mental health stressors, pointing to opportunities for improving HCW well-being [60]. However, a systematic review of the impact

For peer review only - http://bmjopen.bmj.com/site/about/guidelines.xhtml

of political economy on health observed substantial gaps in knowledge, calling for “higher-quality reviews and empirical studies in this area” [61].

Our study suggests that societies marked by higher degrees of equality may be drawn to reinforcing policies that are more protective of vulnerable populations. As health worker protection is so strategically important to health system functioning in times of emerging pandemics, countries known to be highly unequal might accordingly be considered to be in need of even further technical assistance and attention to ensure that there is adequate protection. The evidence we observed that there is a positive association between levels of national inequality and the degree to which HCWs are protected from risk thus also draws attention to the need for better appreciation of the pathways that can explain this, as there is an iterative relationship between the presence of inequalities and the policy regimes that influence the reinforcement or remediation of how further effects are reproduced, for example as expressed in the conditions whereby marginalized groups of workers may encounter risks when a new threat such as a pandemic emerges.

As appreciation of the contribution of HCWs soared as the COVID pandemic advanced, our observations that economic inequality among and within countries is associated with the degree to which HCWs face unacceptable risk and inadequate protection signals a vital need to promote social justice for those who play such an important role in the care of populations *before* a new pandemic emerges. In light of this, from an analytical perspective, we strongly endorse the call for a new paradigm [62] to better understand how upstream and socio-political factors could be “affecting the nature of work and employment and their impact on the health of workers, the

For peer review only - http://bmjopen.bmj.com/site/about/guidelines.xhtml

public, and the planet" [63]. This includes consideration of international cooperation not only with respect to vaccine supply, but also to ensure that less wealthy countries receive technical assistance in establishing protection and mitigation programs as well as attention to pathways sensitive to the offloading of risks to more marginalized worker populations.

Limitations and further research needs

Cross-country comparative studies such ours rely on a convenience sample, leading to some countries being over-represented while others were under-represented or non-existent. To address possible concerns about the influence of countries with low respondent counts, we examined this concern by conducting sensitivity analyses, summarized in Table S7, to consider possible implications, but concluded that this did not warrant a questioning of our findings. Additionally, the classification of countries purely by national income levels leads to designating some countries as high income in settings where national institutions may be minimally developed despite high levels of income earned through high value exports such as petroleum or in settings of small populations with externally controlled tourism sectors. As such, we developed grouping strategies to allow for a consideration of national contexts where resources could be considered comparatively more or less readily available to protect health workers. Stratification by WHO region was also important because these regions, while large and often heterogenous in nature, do constitute administrative units with an important governance role to play during the emergence of global outbreaks and pandemics.

It should also be acknowledged that differing perceptions of risks and mitigation measures around the world may be influenced by different HCW training and education standards, cultural nuances, and institutional expectations. For example, Senthi and colleagues observed that

For peer review only - http://bmjopen.bmj.com/site/about/guidelines.xhtml

workers in India found a high prevalence of workers unable to identify even immediate risks in an evidently hazardous environment [64]. Studies in the Middle East also reported gaps between actual hazards and HCW recognition [65,66]. Ndejjo and colleagues report similar findings in Uganda and across sub-Saharan Africa [67].

CONCLUSION

This study adds to the literature on how risks become unevenly distributed, focusing here on country income level but also on within-country income inequality. As noted by Gostin et al., 2020 [68], the WHO has an important role in supporting LMICs with technical guidance and operational assistance, while simultaneously meeting the needs of high-income countries for information sharing, research coordination, and convening authorities, despite lacking both the authority and the resources to mount a more effective response to a global emergency such as this. Our study strongly suggests that international agencies with mandates related to fair trading practices and economic aid have to step up to address the disparities that threaten the healthcare workforce, and ensure that there is sufficient resilience to retain health workers needed for broader delivery of health services. It is also a matter of social justice that they do so.

For peer review only

For peer review only - http://bmjopen.bmj.com/site/about/guidelines.xhtml

Acknowledgements The authors would like to thank all members of the WHO Occupational Health Ad Hoc Committee as well as all the WHO Occupational Health Collaborating Centres around the world who contributed to the dissemination of the survey.

Contributors SH, AY and JS conceptualised the research. AY and MZ participated in preparation of the original survey and its use. SH prepared the data and conducted the initial analysis. SH and JMS conducted subsequent data analyses and data interpretation. VLT conducted literature reviews. SH wrote the initial manuscript draft. SH, AY, VT, MZ and JMS reviewed and edited the manuscript. AY and JMS accept full responsibility for the finished work and/or the conduct of the study as guarantors, had access to the data, and controlled the decision to publish. All authors read and approved the final manuscript.

Competing interests None declared.

Funding This article has received funding from the International Development Research Centre (M20-00559) and the Canadian Institutes of Health Research (VS1-175519).

Disclaimer The funders did not play a role in the decision to publish the article.

Data availability statement Data are available in the Dryad data depository and are also available from the corresponding author.

Patient consent for publication Not required.

Ethics approval This study involves human participants and was approved by the Behavioural Research Ethics Board of the University of British Columbia (Ref. H20-01825)
As the survey was designed to be completed and submitted anonymously, no formal request for signed consent was solicited, with participants' submission itself indicating consent to use the information provided as anonymized aggregated data.

For peer review only

APPENDIX

Study questionnaire. (Taken from Report on preliminary findings – ref 14.)
(Pdf)

ONLINE SUPPLEMENTAL MATERIAL

SI Dataset. Data underlying the results of the study.
(Excel Data file (XLS). *– to be provided through Dryad data depository*

SI Figure. All supplemental figures.
Figure S1 - Study Population diagram
(pdf)

SI Table. All supplemental tables.
(pdf)

Table S1 - Study population demographics

Table S2 - Summary of factors and their constituent question areas

Table S3 – Variation in factor scores by gender and occupation

Table S4 - Responses to health and safety risk questions

Table S5 - Responses to mitigation measure questions

Table S6 – Factors associated with female patient care providers perceived risk acceptability and mitigation adequacy

Table S7 – Sensitivity analysis considering minimum sample size for calculating country mean values

For peer review only

For peer review only - http://bmjopen.bmj.com/site/about/guidelines.xhtml

REFERENCES

1 International Council of Nurses. ICN calls for data on healthcare worker infection rates and deaths. ICN - International Council of Nurses. https://www.icn.ch/news/icn-calls-data-healthcare-worker-infection-rates-and-deaths (accessed 12 Sep 2022).

2 World Health Organization. World Day for Safety and Health at Work: WHO key facts & key messages to support the day. https://www.who.int/news-room/detail/28-04-2020-who-calls-for-healthy-safe-and-decent-working-conditions-for-all-health-workers-amidst-covid-19-pandemic (accessed 12 Aug 2020).

3 Talisuna A, Yoti Z, Lee C, *et al.* The urgent need for a global commitment to protect healthcare workers. *BMJ Glob Health* 2020;**5**:e004077. doi:10.1136/bmjgh-2020-004077

4 Lancet T. COVID-19: protecting health-care workers. *The Lancet* 2020;**395**:922. doi:10.1016/S0140-6736(20)30644-9

5 Amnesty International. Amnesty analysis reveals over 7,000 health workers have died from COVID-19. https://www.amnesty.org/en/latest/news/2020/09/amnesty-analysis-7000-health-workers-have-died-from-covid19/ (accessed 24 Sep 2020).

6 The World Health Organization. World Health Organization (WHO) on Twitter. Twitter. https://twitter.com/WHO/status/1284148139797209093 (accessed 13 Oct 2020).

7 Papoutsi E, Giannakoulis VG, Ntella V, *et al.* Global burden of COVID-19 pandemic on healthcare workers. *ERJ Open Research* 2020;**6**. doi:10.1183/23120541.00195-2020

8 World Health Organization. Coronavirus disease 2019 (COVID-19) Situation Report – 82.

9 World Health Organization. Global Spending on Health: A World in Transition. WHO. https://www.who.int/publications/i/item/WHO-HIS-HGF-HFWorkingPaper-19.4 (accessed 12 May 2022).

10 World Health Organization. The World Health Report 2006 - working together for health. https://apps.who.int/iris/handle/10665/43432 (accessed 12 May 2022).

11 The World Bank. Physicians (per 1,000 people). https://data.worldbank.org/indicator/SH.MED.PHYS.ZS?most_recent_value_desc=false (accessed 24 Sep 2020).

12 McDiarmid MA. Hazards of the Health Care Sector: Looking Beyond Infectious Disease. *Annals of Global Health* 2014;**80**:315–9. doi:10.1016/j.aogh.2014.08.001

13 Caeiro JP, Garzón MI. Controlling infectious disease outbreaks in low-income and middle-income countries. *Curr Treat Options Infect Dis* 2018;**10**:55–64. doi:10.1007/s40506-018-0154-z

For peer review only - http://bmjopen.bmj.com/site/about/guidelines.xhtml

14 Mossburg S, Agore A, Nkimbeng M, *et al.* Occupational Hazards among Healthcare Workers in Africa: A Systematic Review. *Ann Glob Health* 2019;**85**. doi:10.5334/aogh.2434

15 Gross JV, Mohren J, Erren TC. COVID-19 and healthcare workers: a rapid systematic review into risks and preventive measures. *BMJ Open* 2021;**11**:e042270. doi:10.1136/bmjopen-2020-042270

16 Harrigan SP, Tsang VWL, Spiegel JM, *et al.* Health and safety of health workers in the context of COVID-19: A global survey. WHO Collaborating Centre, University of British Columbia http://med-fom-ghrp-spph.sites.olt.ubc.ca/files/2020/09/WHO_HCW_Survey_COVID-19-report_MASTER.pdf

17 Nowatzki NR. Wealth inequality and health: a political economy perspective. *Int J Health Serv* 2012;**42**:403–24. doi:10.2190/HS.42.3.c

18 Subramanian SV, Kawachi I. Income Inequality and Health: What Have We Learned So Far? *Epidemiol Rev* 2004;**26**:78–91. doi:10.1093/epirev/mxh003

19 Pickett KE, Wilkinson RG. Income inequality and health: a causal review. *Soc Sci Med* 2015;**128**:316–26. doi:10.1016/j.socscimed.2014.12.031

20 Fujishiro K, Ahonen EQ, Gimeno Ruiz de Porras D, *et al.* Sociopolitical values and social institutions: Studying work and health equity through the lens of political economy. *SSM Popul Health* 2021;**14**:100787. doi:10.1016/j.ssmph.2021.100787

21 Adu PA, Spiegel JM, Yassi A. Towards TB elimination: how are macro-level factors perceived and addressed in policy initiatives in a high burden country? *Globalization and Health* 2021;**17**:11. doi:10.1186/s12992-020-00657-1

22 Gelfand MJ, Jackson JC, Pan X, *et al.* The relationship between cultural tightness–looseness and COVID-19 cases and deaths: a global analysis. *The Lancet Planetary Health* 2021;**5**:e135–44. doi:10.1016/S2542-5196(20)30301-6

23 Haug N, Geyrhofer L, Londei A, *et al.* Ranking the effectiveness of worldwide COVID-19 government interventions. *Nat Hum Behav* 2020;**4**:1303–12. doi:10.1038/s41562-020-01009-0

24 Brauner JM, Mindermann S, Sharma M, *et al.* Inferring the effectiveness of government interventions against COVID-19. *Science* 2021;**371**. doi:10.1126/science.abd9338

25 Rinaldi C, Bekker MPM. A Scoping Review of Populist Radical Right Parties' Influence on Welfare Policy and its Implications for Population Health in Europe. *International Journal of Health Policy and Management* 2021;**10**:141–51. doi:10.34172/ijhpm.2020.48

26 World Health Organization. Definition of regional groupings. WHO. http://www.who.int/healthinfo/global_burden_disease/definition_regions/en/ (accessed 12 Aug 2020).

For peer review only

27 The World Bank. World Bank Country and Lending Groups – World Bank Data Help Desk. https://datahelpdesk.worldbank.org/knowledgebase/articles/906519-world-bank-country-and-lending-groups (accessed 12 Aug 2020).

28 The World Bank. GINI index (World Bank estimate). https://data.worldbank.org/indicator/SI.POV.GINI (accessed 12 Aug 2020).

29 Ritchie H, Ortiz-Ospina E, Beltekian D, *et al.* Coronavirus (COVID-19) Testing - Statistics and Research. Our World in Data. https://ourworldindata.org/coronavirus-testing (accessed 30 Nov 2020).

30 Taber KS. The Use of Cronbach's Alpha When Developing and Reporting Research Instruments in Science Education. *Res Sci Educ* 2018;**48**:1273–96. doi:10.1007/s11165-016-9602-2

31 R Core Team. *R: A language and environment for statistical computing*. R Foundation for Statistical Computing, Vienna, Austria. 2013. http://www.R-project.org/

32 IBM Corp. *SPSS*. 2018.

33 Shreffler J, Petrey J, Huecker M. The Impact of COVID-19 on Healthcare Worker Wellness: A Scoping Review. *West J Emerg Med* 2020;**21**:1059–66. doi:10.5811/westjem.2020.7.48684

34 Mhango M, Dzobo M, Chitungo I, *et al.* COVID-19 Risk Factors Among Health Workers: A Rapid Review. *Safety and Health at Work* 2020;**11**:262–5. doi:10.1016/j.shaw.2020.06.001

35 Bandyopadhyay S, Baticulon RE, Kadhum M, *et al.* Infection and mortality of healthcare workers worldwide from COVID-19: a systematic review. *BMJ Glob Health* 2020;**5**:e003097. doi:10.1136/bmjgh-2020-003097

36 Afulani PA, Nutor JJ, Agbadi P, *et al.* Job satisfaction among healthcare workers in Ghana and Kenya during the COVID-19 pandemic: Role of perceived preparedness, stress, and burnout. *PLOS Global Public Health* 2021;**1**:e0000022. doi:10.1371/journal.pgph.0000022

37 Gómez-Ochoa SA, Franco OH, Rojas LZ, *et al.* COVID-19 in Healthcare Workers: A Living Systematic Review and Meta-analysis of Prevalence, Risk Factors, Clinical Characteristics, and Outcomes. *Am J Epidemiol* Published Online First: 1 September 2020. doi:10.1093/aje/kwaa191

38 Zungu M, Voyi K, Mlangeni N, *et al.* Organizational factors associated with health worker protection during the COVID-19 pandemic in four provinces of South Africa. *BMC Health Services Research* 2021;**21**:1080. doi:10.1186/s12913-021-07077-w

39 Jeleff M, Traugott M, Jirovsky-Platter E, *et al.* Occupational challenges of healthcare workers during the COVID-19 pandemic: a qualitative study. *BMJ Open* 2022;**12**:e054516. doi:10.1136/bmjopen-2021-054516

For peer review only

40 Pappa S, Ntella V, Giannakas T, *et al.* Prevalence of depression, anxiety, and insomnia among healthcare workers during the COVID-19 pandemic: A systematic review and meta-analysis. *Brain Behav Immun* 2020;**88**:901–7. doi:10.1016/j.bbi.2020.05.026

41 Krishnamoorthy Y, Nagarajan R, Saya GK, *et al.* Prevalence of psychological morbidities among general population, healthcare workers and COVID-19 patients amidst the COVID-19 pandemic: A systematic review and meta-analysis. *Psychiatry Res* 2020;**293**:113382. doi:10.1016/j.psychres.2020.113382

42 Salazar de Pablo G, Vaquerizo-Serrano J, Catalan A, *et al.* Impact of coronavirus syndromes on physical and mental health of health care workers: Systematic review and meta-analysis. *J Affect Disord* 2020;**275**:48–57. doi:10.1016/j.jad.2020.06.022

43 Brier ND, Stroobants S, Vandekerckhove P, *et al.* Factors affecting mental health of health care workers during coronavirus disease outbreaks (SARS, MERS & COVID-19): A rapid systematic review. *PLOS ONE* 2020;**15**:e0244052. doi:10.1371/journal.pone.0244052

44 Dye TD, Alcantara L, Siddiqi S, *et al.* Risk of COVID-19-related bullying, harassment and stigma among healthcare workers: an analytical cross-sectional global study. *BMJ Open* 2020;**10**:e046620. doi:10.1136/bmjopen-2020-046620

45 Atnafie SA, Anteneh DA, Yimenu DK, *et al.* Assessment of exposure risks to COVID-19 among frontline health care workers in Amhara Region, Ethiopia: A cross-sectional survey. *PLoS One* 2021;**16**:e0251000. doi:10.1371/journal.pone.0251000

46 Arslanca T, Fidan C, Daggez M, *et al.* Knowledge, preventive behaviors and risk perception of the COVID-19 pandemic: A cross-sectional study in Turkish health care workers. *PLoS One* 2021;**16**:e0250017. doi:10.1371/journal.pone.0250017

47 Conti C, Fontanesi L, Lanzara R, *et al.* Fragile heroes. The psychological impact of the COVID-19 pandemic on health-care workers in Italy. *PLoS One* 2020;**15**:e0242538. doi:10.1371/journal.pone.0242538

48 Garralda Fernandez J, Molero Vilches I, Bermejo Rodríguez A, *et al.* Impact of SARS-CoV-2 pandemic among health care workers in a secondary teaching hospital in Spain. *PLoS One* 2021;**16**:e0245001. doi:10.1371/journal.pone.0245001

49 Shone E. More than 850 health and social care workers have died of Covid in England and Wales since the pandemic began. The Scotsman. https://www.scotsman.com/health/coronavirus/more-than-850-health-and-social-care-workers-have-died-of-covid-in-england-and-wales-since-the-pandemic-began-3114202 (accessed 10 Jun 2021).

50 Our key findings about US healthcare worker deaths in the pandemic's first year. The Guardian. https://www.theguardian.com/us-news/ng-interactive/2020/dec/22/lost-on-the-frontline-our-findings-to-date (accessed 10 Jun 2021).

For peer review only

51 Kavanagh MM, Gostin LO, Sunder M. Sharing Technology and Vaccine Doses to Address Global Vaccine Inequity and End the COVID-19 Pandemic. *JAMA* 2021;**326**:219–20. doi:10.1001/jama.2021.10823

52 Lim DW, Htun HL, Wang Y, *et al.* Healthcare workers as "canaries" for acute respiratory infections and pathogens during the COVID-19 pandemic. *J Hosp Infect* 2021;**112**:119–20. doi:10.1016/j.jhin.2021.03.019

53 McMahon DE, Peters GA, Ivers LC, *et al.* Global resource shortages during COVID-19: Bad news for low-income countries. *PLOS Neglected Tropical Diseases* 2020;**14**:e0008412. doi:10.1371/journal.pntd.0008412

54 Shears P, O'Dempsey TJD. Ebola virus disease in Africa: epidemiology and nosocomial transmission. *Journal of Hospital Infection* 2015;**90**:1–9. doi:10.1016/j.jhin.2015.01.002

55 Kim H, Hegde S, LaFiura C, *et al.* Access to personal protective equipment in exposed healthcare workers and COVID-19 illness, severity, symptoms and duration: a population-based case-control study in six countries. *BMJ Glob Health* 2021;**6**:e004611. doi:10.1136/bmjgh-2020-004611

56 Blázquez-Fernández C, Cantarero-Prieto D, Pascual-Saez M. Does Rising Income Inequality Reduce Life Expectancy? New Evidence for 26 European Countries (1995–2014). *Global Economic Review* 2018;**47**:464–79. doi:10.1080/1226508X.2018.1526098

57 Mellor JM, Milyo J. Is Exposure to Income Inequality a Public Health Concern? Lagged Effects of Income Inequality on Individual and Population Health. *Health Serv Res* 2003;**38**:137–51. doi:10.1111/1475-6773.00109

58 Odusanya IA, Akinlo AE. Income Inequality and Population Health in Sub-Saharan Africa: A Test of Income Inequality-Health Hypothesis. *Journal of Population and Social Studies [JPSS]* 2021;**29**:235–54.

59 Kim I-H, Muntaner C, Vahid Shahidi F, *et al.* Welfare states, flexible employment, and health: A critical review. *Health Policy* 2012;**104**:99–127. doi:10.1016/j.healthpol.2011.11.002

60 Sandal A, Yildiz AN. COVID-19 as a Recognized Work-Related Disease: The Current Situation Worldwide. *Saf Health Work* 2021;**12**:136–8. doi:10.1016/j.shaw.2021.01.001

61 McCartney G, Hearty W, Arnot J, *et al.* Impact of Political Economy on Population Health: A Systematic Review of Reviews. *Am J Public Health* 2019;**109**:e1–12. doi:10.2105/AJPH.2019.305001

62 Flynn MA, Check P, Steege AL, *et al.* Health Equity and a Paradigm Shift in Occupational Safety and Health. *Int J Environ Res Public Health* 2021;**19**:349. doi:10.3390/ijerph19010349

For peer review only - http://bmjopen.bmj.com/site/about/guidelines.xhtml

63 Loewenson R. Rethinking the Paradigm and Practice of Occupational Health in a World Without Decent Work: A Perspective From East and Southern Africa. *New Solut* 2021;**31**:107–12. doi:10.1177/10482911211017106

64 Senthil A, Anandh B, Jayachandran P, *et al.* Perception and prevalence of work-related health hazards among health care workers in public health facilities in southern India. *Int J Occup Environ Health* 2015;**21**:74–81. doi:10.1179/2049396714Y.0000000096

65 Muralidhar S, Singh PK, Jain RK, *et al.* Needle stick injuries among health care workers in a tertiary care hospital of India. *Indian J Med Res* 2010;**131**:405–10.

66 Kotwal A, Taneja D. Health Care Workers and Universal Precautions: Perceptions and Determinants of Non-compliance. *Indian J Community Med* 2010;**35**:526–8. doi:10.4103/0970-0218.74373

67 Ndejjo R, Musinguzi G, Yu X, *et al.* Occupational Health Hazards among Healthcare Workers in Kampala, Uganda. *Journal of Environmental and Public Health*. 2015;**2015**:e913741. doi:https://doi.org/10.1155/2015/913741

68 Gostin LO, Moon S, Meier BM. Reimagining Global Health Governance in the Age of COVID-19. *Am J Public Health* 2020;**110**:1615–9. doi:10.2105/AJPH.2020.305933

For peer review only

Appendices

Survey instrument

Health and safety of health workers in COVID-19

Welcome to the survey on health and safety of health workers in COVID-19

Dear colleague,

This survey aims to identify the most common occupational risks for the health and safety of health workers and the measures for their prevention in the context of the ongoing pandemic of Corona Virus Infectious Disease (COVID-19).

In this survey we are interested in the health and safety of all health workers - all people engaged in the promotion, protection or improvement of the health of the population. This includes health workers involved in direct patient care, both formal and informal, in public and private facilities, including traditional medicine, as well as other assisting and supporting staff, including administration, management, ambulance drivers, public health workers, community health workers, and others.

The survey is intended for health workers, managers, and practitioners providing services for protecting the health and safety of workers in health facilities. The results will be used to inform action at all levels for improving the protection of health and safety of health workers in the ongoing response to COVID-19.

The survey has been developed by an international group of experts convened by the World Health Organization and the International Labour Organization and should take approximately 7 minutes to complete.

Your answers are completely confidential, and the data will be processed and analyzed in a way that will not link your answers to your identity.

B. About yourself and your area of work

1. In what country do you usually work?

Standard list of all countries in the world

Other (please specify)

* 2. Your gender?

Male

Female

Other

Prefer not to answer

For peer review only - http://bmjopen.bmj.com/site/about/guidelines.xhtml

3. What is your primary area of work? (responses below were randomized)

Administration and clerical support
Allied health professional
Community health worker
Infection prevention and control
Management and human resources
Mental health and psychosocial support
Occupational and environmental health
Patient care (medicine, nursing, midwifery, dentistry)
Pharmacy
Public health
Support staff – cleaner, driver, food worker
Other

4. You work most of the time for: (responses below were randomized)

Academia, research
Business enterprise or farm·
Employers' association /hospital federation
Healthcare facility - hospital, primary health-care centre, isolation camp
Local community
National government agency
Other
Professional association
Social care facility (e.g. nursing home, home care)·
Sub-national (provincial, district) authority
Trade union

C. Risks for health and safety of health workers

Think about the working conditions of health workers in your country, jurisdiction or health facility - those that you are most familiar with. No workplace is without risk, but some risks are negligible, or acceptable for a short time, and some are not acceptable at all. Below are some common risks for the health and safety of health workers; we are asking you to rate the current level of these risks, now during the COVID-19 pandemic.

For peer review only

5. How would you rate the level of these risks for health workers, now? (randomized)

Questions	Risk is negligible	Risk is acceptable for a short time	Risk is not acceptable at all	Don't know/Unsure
Skin damage from personal protective equipment and/or frequent hand hygiene				
Needle-sticks and sharps injuries				
Inadequate sanitation facilities				
Insufficient access to facilities for personal hygiene, such as, shower and menstrual hygiene				
Exposure to blood, body fluids, respiratory secretions, and other potentially infectious materials				

6. How would you rate the level of these risks for health workers, now? (randomized)

Questions	Risk is negligible	Risk is acceptable for a short time	Risk is not acceptable at all	Don't know/Unsure
Back injury from manual handling of patients and heavy objects				
Hazardous chemicals, drugs, cleaning and disinfection agents				
Slips, trips, and falls				
Crowded workplace				
Thermal discomfort (cold, heat, humidity)				

7. How would you rate the level of these risks for health workers, now? (randomized)

Questions	Risk is negligible	Risk is acceptable for a short time	Risk is not acceptable at all	Don't know/Unsure
Physical violence and assaults				
Bullying or psychological harassment at the workplace				
Sexual harassment				

For peer review only - http://bmjopen.bmj.com/site/about/guidelines.xhtml

8. How would you rate the level of these risks for health workers, now? (randomized)

Questions	Risk is negligible	Risk is acceptable for a short time	Risk is not acceptable at all	Don't know/Unsure
Regular long working hours (more than 48 hours a week)				
Time pressure, high workload				
Shift work with night shifts				
Insufficient time-off duty to rest (less than 11 hours between shifts)				

9. How would you rate the level of these risks for health workers, now? (randomized)

Questions	Risk is negligible	Risk is acceptable for a short time	Risk is not acceptable at all	Don't know/Unsure
Skin damage from personal protective equipment and/or frequent hand hygiene				
Needle-sticks and sharps injuries				
Inadequate sanitation facilities				
Insufficient access to facilities for personal hygiene, such as, shower and menstrual hygiene				
Exposure to blood, body fluids, respiratory secretions, and other potentially infectious materials				

D. Preventive measures

There are measures for the prevention of most risks for health and safety at work, but these measures may not be fully implemented and not all workers may benefit from these measures. Think again about the working conditions of health workers in your country, jurisdiction or health facility - those that you are most familiar with. The following questions are about the preventive measures for their health and safety in the real situation, now, during the COVID-19 pandemic.

For peer review only - http://bmjopen.bmj.com/site/about/guidelines.xhtml

10. How would you rate the level of application of these measures in the health services according to your knowledge? (randomized)

Questions	Does not exist at all	Exists and offers some protection	Exists and offers full protection	Don't know/Unsure
Policy for infection prevention and control in the health facility				
Processes for triage of patient in place at the emergency room, including early detection and isolation of infectious patients				
Routine assessment of the risk of exposure to body substances or contaminated surfaces before any health care activity and use of appropriate measures for personal protection				
Regular environmental clean-up and disinfection				
Immunization of health workers				

11. How would you rate the level of application of these measures in the health services according to your knowledge? (randomized)

Questions	Does not exist at all	Exists and offers some protection	Exists and offers full protection	Don't know/Unsure
Reporting of incidental exposures to blood, body fluids, or respiratory secretions				
Policies in place for post-exposure prophylaxis, such as, for HIV, Hepatitis B				
Facilities for hand hygiene (hand washing and disinfection) are readily available				
Personal protective equipment, such as masks, gloves, goggles, gowns are readily available				
Training and education of workers about infection prevention and control				

For peer review only - http://bmjopen.bmj.com/site/about/guidelines.xhtml

12. How would you rate the level of application of these measures in health services according to your knowledge? (randomized)

Questions	Does not exist at all	Exists and offers some protection	Exists and offers full protection	Don't know/Unsure
Prevention of workplace violence and security measures				
Management of working time, rest and recuperation				
Workplace policies against bullying, psychological and sexual harassment				
Human resource management of safe staffing and workload				
Psycho-social support and counselling				

13. How would you rate the level of application of these measures in the health services according to your knowledge? (randomized)

Questions	Does not exist at all	Exists and offers some protection	Exists and offers full protection	Don't know/Unsure
Occupational safety and health policy and management system in the facility				
Regular assessment of workplace health and safety risks and controls				
Engineering controls, such as ventilation, physical barriers, safer devices				
Ergonomic workplace design and furniture				
Devices for patient handling and lifting of loads				

14. How would you rate the level of application of these measures in the health services according to your knowledge? (randomized)

Questions	Does not exist at all	Exists and offers some protection	Exists and offers full protection	Don't know/Unsure
Regular medical check-ups of health workers				
Medical first aid kits				
Consultations between management and workers regarding health and safety at work				
Training and education of workers about occupational safety and health				

Other (please specify)

For peer review only - http://bmjopen.bmj.com/site/about/guidelines.xhtml

Table A 1: Survey options for occupation

Group	Survey responses
Patient care/health services	Patient care (medicine, nursing, midwifery, dentistry)
	Allied health professional
	Mental health and psychosocial support
	Pharmacy
	Community health worker
Specialized support	Occupational and environmental health
	Public health
	Infection prevention and control
	Support staff – cleaner, driver, food worker
Clerical support/administration and management	Administration and clerical support
	Management and human resources

Table A 2: Comparison between participants who answered risk and mitigation questions

**Variables**		**Those who responded to risk questions (n=4977)**	**Those who responded to mitigation measure questions (n=4076)**
Countries by region	AFRO	10%	11%
	EMRO	4%	4%
	EURO	35%	36%
	PAHO	31%	31%
	SEARO	3%	3%
	WPRO	15%	16%
Economic Class	High	59%	60%
	Lower-middle	10%	10%
	Upper-middle	27%	26%
	Low	4%	4%
Sex	Male	33%	34%
	Female	65%	65%
	Other / prefer not to answer	1%	1%
Occupation	Patient care/health services	56%	58%
	Specialized support	29%	29%
	Clerical support/administration and management	7%	7%
	Other	8%	7%

For peer review only - http://bmjopen.bmj.com/site/about/guidelines.xhtml

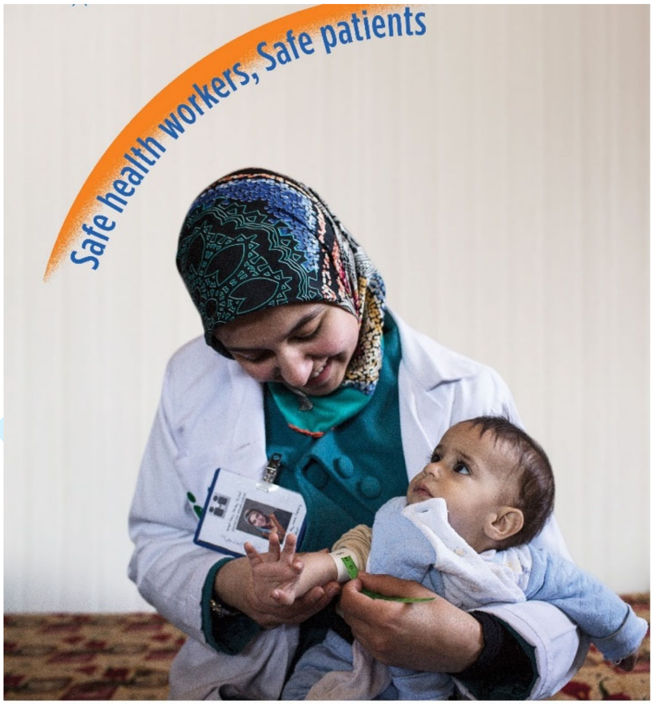

Speak up
for health worker safety!

World
Patient Safety
Day 17 September 2020

Health and safety of health workers in the context of COVID-19: A global survey

Sean P. Harrigan, Vivian W. L. Tsang, Jerry M. Spiegel, Annalee Yassi

September 15, 2020

a place of mind
THE UNIVERSITY OF BRITISH COLUMBIA
Faculty of Medicine
School of Population and Public Health

GLOBAL HEALTH
RESEARCH PROGRAM

WHO Collaborating Centre
for Occupational Health

© Global Health Research Program, School of Population and Public Health,

For peer review only - http://bmjopen.bmj.com/site/about/guidelines.xhtml

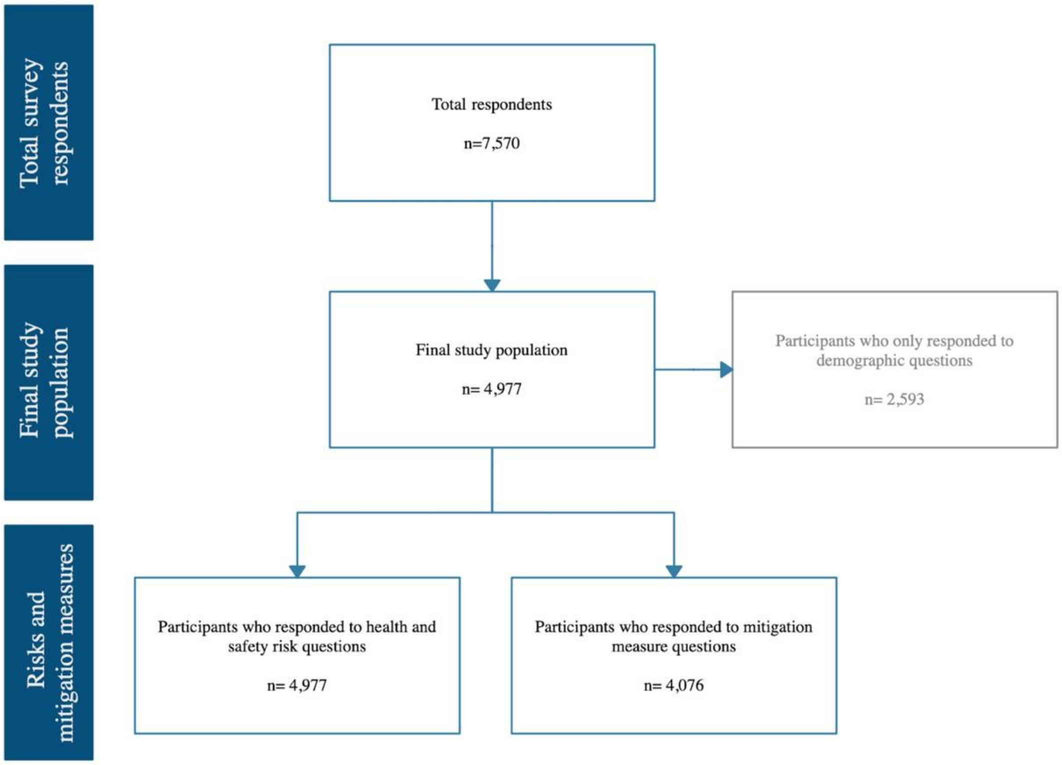
Total survey respondents
Final study population
Risks and mitigation measures
Total respondents
n=7,570
Final study population
n= 4,977
Participants who only responded to demographic questions
n= 2,593
Participants who responded to health and safety risk questions
n= 4,977
Participants who responded to mitigation measure questions
n= 4,076

ONLINE SUPPLEMENTARY INFORMATION

Table S1. Study population demographics

Variables		n	%
Total number of participants		4977	100%
Total who replied to the health and safety risks for health workers questions		4977	100%
Total who replied to the mitigation measures questions		4076	82%
Total number of countries		161	100%
Countries by region	African region	516	10%
	Americas region	1565	31%
	Eastern Mediterranean region	221	4%
	European region	1757	35%
	South East Asian region	158	3%
	Western Pacific region	760	15%
Economic Class	High	2960	59%
	Lower-middle	480	10%
	Upper-middle	1324	27%
	Low	213	4%
Sex	Male	1654	33%
	Female	3259	65%
	Other / prefer not to answer	64	1%
Occupation	Patient care/health services	2805	56%
	Specialized support	1426	29%
	Clerical support/administration and management	341	7%
	Other	405	10%
Type of employer	Health services	3038	61%
	Government services	758	15%
	Business and farms	473	10%
	Academia	367	7%
	Professional associations	177	4%
	International organization/NGO and non-for-profit	51	1%
	Other	113	2%

For peer review only

For peer review only - http://bmjopen.bmj.com/site/about/guidelines.xhtml

Table S2. Summary of Factors and their constituent question areas

Survey area of focus	Factor	Description of question
Health and Safety Risks	Work Environment (Factor 1)	Blood and bodily fluids exposure
		Skin damage from PPE and hand hygiene
		Needlesticks and sharps injuries
		Sanitation facilities
		Personal hygiene
		Thermal discomfort
		Crowded workplace
		Slips trips & falls
		Back injury form heavy lifting
		Chemicals
		Bullying & harassment
		Sexual harassment
	Work Organization (Factor 2)	Physical violence and assaults
		Time pressure
		Shift work
		Long working hours
		Insufficient rest
Mitigation measures	Infection protection and control (Factor 3)	IPC policy
		Patient triage
		Standard precautions
		Cleanup & disinfection
		Immunization
		Reporting of blood exposure
		Policies for post-exposure prophylaxis
		Hand hygiene
		PPE
		IPC training
	Occupational health and safety (Factor 4)	OSH policy
		Occupational health and safety risk assessment
		Occupational health and safety engineering controls
		Ergonomic workplace design and furniture
		Safe patient handling
		Violence prevention
		Management of working time & rest
		Policy harassment
		Safe staffing & workload
		Psychosocial support
		Medical checkups
		Medical first aid kits
		Labour management consultation on OSH
		Occupational health and safety training

For peer review only - http://bmjopen.bmj.com/site/about/guidelines.xhtml

Table S3: Detailed breakdowns in individual level responses by occupation and gender

a. Detailed breakdowns in responses by occupation showing gender differences

Population characteristic	n[c]	Risk acceptability: Work environment mean	p	Risk acceptability: Work organization mean	p	Mitigation adequacy: IPC mean	p	Mitigation adequacy: OHS [f3] mean	P
All Occupations	4916	**3.88**	**0.04**	3.87	0.101	**4.79**	**<0.01**	6.28	0.19
m		***4.11***	***<0.01***	*3.92*	*0.397*	*4.88*	*0.089*	*6.19*	*0.07*
f		***3.76***		*3.85*		*4.74*		*6.33*	
- Patient Care	2792	**3.92**	***0.001***	3.88	*0.296*	**4.63**	*0.76*	6.27	*0.092*
m		***4.19***		*3.96*		*4.65*		*6.15*	
f		***3.80***		*3.84*		*4.62*		*6.32*	
- Specialist	1404	**3.84**	*0.129*	3.80	*0.669*	**4.90**	*0.218*	6.30	*0.193*
m		*3.97*		*3.77*		*5.00*		*6.20*	
f		*3.73*		*3.83*		*4.81*		*6.39*	
- Admin-Mgr	327	**4.14**	*0.06*	4.22	*0.632*	**5.50**	*0.512*	6.50	*0.872*
m		*4.65*		*4.33*		*5.65*		*6.54*	
f		*3.92*		*4.17*		*5.43*		*6.48*	
- Other	393	**3.55**	*0.205*	3.82	*0.189*	**5.03**	*0.20*	6.08	*0.89*
m		*3.82*		*4.10*		*5.30*		*6.11*	
f		*3.43*		*3.69*		*4.90*		*6.08*	

Note: Statistical significance at p<.05 level indicated by bold; statistical significance at p<.10 level indicated by underlining.
Italic font is used for breakdown analysis (i.e. by gender) of population attribute (regular font) being analyzed (i.e. by occupation) and values are right-justified.
Regular font is used for analysis of the population attribute (regular font) being analyzed (i.e. by occupation).
Green colour indicates significant value deemed as associated with less risk (i.e. less unacceptable exposure; more adequate mitigation.
Red colour indicates statistically significant value associated with more risk (i.e. more unacceptable exposure; less adequate mitigation.

For peer review only

b. **Detailed breakdowns in responses by occupation showing gender differences**

Population characteristic	n[c]	Risk acceptability: Work environment mean	p	Risk acceptability: Work organization mean	p	Mitigation adequacy: IPC mean	p	Mitigation adequacy: OHS [f3] mean	P
Total	4863	**3.88**	**<0.01***	3.87	0.4	4.79	0.09	6.28	0.07
Patient Care		***3.91***		*3.88*		*4.63*		*6.27*	
Specialist		***3.84***		*3.80*		*4.90*		*6.30*	
Admin-Mgr		***4.14***		*4.22*		*5.50*		*6.50*	
Other		***3.55***		*3.82*		*5.03*		*6.08*	
Female	3220	**3.76**	0.189	3.85	0.273	4.74	**<0.01***	6.33	0.288
Patient Care		*3.80*		*3.84*		***4.62***		*6.32*	
Specialist		*3.73*		*3.83*		*4.82*		*6.39*	
Admin-Mgr		*3.92*		*4.17*		***5.43***		*6.48*	
Other		*3.43*		*3.69*		*4.90*		*6.07*	
Male	1643	**4.11**	0.089	3.92	0.166	4.88	**0.001**	6.19	0.527
Patient Care		*4.19*		*3.96*		***4.65***		*6.15*	
Specialist		*3.97*		*3.77*		*5.00*		*6.20*	
Admin-Mgr		*4.65*		*4.33*		***5.65***		*6.54*	
Other		*3.82*		*4.10*		*5.30*		*6.11*	

Note: Statistical significance at p<.05 level indicated by bold; statistical significance at p<.10 level indicated by underlining.
Italic font is used for breakdown analysis (i.e. by occupation) of population attribute (regular font) being analyzed (i.e. by gender) and values are right-justified.
Regular font is used for analysis of the population attribute (regular font) being analyzed (i.e. by gender).
Green colour indicates significant value deemed as associated with less risk (i.e. less unacceptable exposure; more adequate mitigation).
Red colour indicates statistically significant value associated with more risk (i.e. more unacceptable exposure; less adequate mitigation).

For peer review only - http://bmjopen.bmj.com/site/about/guidelines.xhtml

Table S4. Responses to health and safety risk questions

Question	Risk is not acceptable at all	Risk is acceptable for a short time	Risk is negligible	Don't know/unsure
**Infectious risks**				
Exposure to blood, body fluids, respiratory secretions, and other potentially infectious materials	**52%**	29%	15%	4%
Skin damage from personal protective equipment and/or frequent hand hygiene	33%	**46%**	16%	5%
Needle-sticks and sharps injuries	**46%**	21%	26%	7%
Inadequate sanitation facilities	**52%**	21%	23%	4%
Insufficient access to facilities for personal hygiene, such as, shower and menstrual hygiene	**49%**	22%	23%	6%
**Physical work environment**				
Thermal discomfort (cold, heat, humidity)	25%	**46%**	24%	5%
Crowded workplace	**42%**	36%	18%	4%
Slips, trips, and falls	**34%**	26%	33%	7%
Back injury from manual handling of patients and heavy objects	**41%**	34%	19%	6%
Hazardous chemicals, drugs, cleaning and disinfection agents	**36%**	**36%**	22%	6%
**Psychosocial work environment**				
Bullying or psychological harassment at the workplace	**54%**	18%	21%	7%
Sexual harassment	**50%**	10%	31%	9%
Physical violence and assaults	**54%**	16%	24%	6%
**Work organization**				
Time pressure, high workload	38%	**49%**	10%	3%
Shift work with night shifts	23%	**48%**	21%	8%
Regular long working hours (more than 48 hours a week)	**38%**	42%	15%	5%
Insufficient time-off duty to rest (less than 11 hours between shifts)	**40%**	36%	18%	6%

Note: Most cited response highlighted in bold

For peer review only - http://bmjopen.bmj.com/site/about/guidelines.xhtml

Table S5. Responses to mitigation measure questions

Question	Does not exist at all	Exists and offers some protection	Exists and offers full protection	Don't know/ unsure
**Infection prevention and control**				
IPC policy in the health facility	8%	**60%**	28%	4%
Patient triage	9%	**54%**	28%	9%
Standard precautions	15%	**53%**	25%	7%
Regular environmental clean-up and disinfection	6%	**56%**	34%	4%
Immunization of health workers	19%	**46%**	26%	9%
Reporting of incidental exposures to blood, body fluids, or respiratory secretions	10%	**50%**	32%	8%
Policies in place for post-exposure prophylaxis, such as, for HIV, Hepatitis B	8%	41%	**42%**	9%
Facilities for hand hygiene (hand washing and disinfection) are readily available	3%	40%	**54%**	3%
Personal protective equipment, such as masks, gloves, goggles, gowns are readily available	8%	**55%**	34%	3%
Training and education of workers about infection prevention and control	11%	**54%**	32%	3%
**Occupational safety and health**				
Occupational safety and health policy and management system in the facility	14%	**58%**	22%	6%
Regular assessment of workplace health and safety risks and controls	22%	**51%**	21%	6%
Engineering controls, such as ventilation, physical barriers, safer devices	19%	**54%**	19%	8%
Ergonomic workplace design and furniture	33%	**48%**	12%	7%
Devices for patient handling and lifting of loads	27%	**48%**	15%	10%
Prevention of workplace violence and security measures	21%	**52%**	21%	6%
Management of working time, rest and recuperation	20%	**55%**	19%	6%
Workplace policies against bullying, psychological and sexual harassment	27%	**43%**	21%	9%
Human resource management of safe staffing and workload	24%	**52%**	16%	8%
Psycho-social support and counselling	32%	**46%**	15%	7%
Regular medical check-ups of health workers	33%	**43%**	18%	6%
Medical first aid kits	15%	**48%**	30%	7%
Consultations between management and workers regarding health and safety at work	25%	**50%**	19%	6%
Training and education of workers about occupational safety and health	20%	**54%**	21%	5%

Note: Most cited response highlighted in bold

For peer review only - http://bmjopen.bmj.com/site/about/guidelines.xhtml

Table S6: Comparing analyses of acceptability and adequacy in different stratified populations

	Patient Care Female		Patient Care All		Frontline[e] all		All occupations	
**Countries included**	**112**		**133**		**156**		**161**	
**Explanatory Variable** (organized by outcome area)	**OR[d]**	**p**	**OR[d]**	**p**	**OR[d]**	**p**	**OR[d]**	**p**
**Acceptable WP Enviro. Risk**								
Country Income Level [a]	0.81	0.736	0.63	0.415	1.01	0.981	1.24	0.708
**Gini Coefficient** [b]	0.98	0.609	0.96	0.1991	***0.94***	***0.068*****	**0.92**	**0.012***
COVID-Log [c]	1.12	0.777	1.33	0.398	1.22	0.549	1.09	0.801
**Acceptable WP Org. Risk**								
Country Income Level [a]	1.30	0.657	1.57	0.446	1.06	0.926	0.71	0.52
**Gini Coefficient** [b]	***0.95***	***0.093*****	**0.93**	**0.024***	***0.93***	***0.056*****	**0.95**	**0.017***
**COVID-Log** [c]	**0.44**	**0.034***	0.66	0.248	0.98	0.951	0.89	0.710
**Adequate IPC mitigation**								
Country Income Level [a]	**11.25**	**0.004***	**7.48**	**0.006***	**6.92**	**0.001***	**6.61**	**0.001***
**Gini Coefficient** [b]	0.95	0.220	0.95	0.125	**0.95**	**0.047***	**0.94**	**0.025***
COVID-Log [c]	0.69	0.479	0.7	0.181	0.70	0.264	0.76	0.373
**Adequate OHS mitigation**								
Country Income Level [a]	**3.94**	**0.047***	**5.86**	**0.004***	**18.39**	**<0.001***	**10.91**	**<0.001***
**Gini Coefficient** [b]	**0.92**	**0.020***	***0.95***	***0.083*****	0.99	0.755	0.99	0.779
COVID-Log [c]	0.79	0.556	0.69	0.281	0.99	0.988	1.08	0.816

[a] Country Income was coded as comparing "High and Upper-Middle Income" countries versus "Low and Lower-Middle Income" countries

[b] Gini coefficient was considered in the logistic regression analysis as a continuous variable;

[c] COVID levels where the log value of the rate of cases per million at the beginning of the survey (taken June 1, 2020); log values to smooth very high levels while taking variation into account

[d] Odds Ratios were calculated by assessing the likelihood (OR) of the presence of a mean scores ≥ 5 corresponding to assessments that mitigation "exists and offers some protection" or better; or level of risk is assessed as "risk is acceptable for a short time" or better.

[e] includes all patient care and workplace specialist support personnel such as OHS and IPC professionals

* Indicates statistical significance (p<0.05) and also bolded; ** p<.10 but >.05; indicated in italics and bold

For peer review only

For peer review only - http://bmjopen.bmj.com/site/about/guidelines.xhtml

Table S7: Comparing analyses when minimum country sample size provisions are applied

	Patient Care Female		Patient Care Female		Frontline[e]		Frontline[e]	
**Minimum country sample**	**2**		**all**		**4**		**all**	
**Countries included**	**80**		**112**		**101**		**161**	
**Explanatory Variable** (organized by outcome area)	**OR[d]**	**p**	**OR[d]**	**p**	**OR[d]**	**p**	**OR[d]**	**p**
**Acceptable WP Enviro. Risk**								
Country Income Level [a]	0.62	0.556	0.81	0.736	1.03	0.967	1.01	0.981
**Gini Coefficient** [b]	0.97	0.404	0.98	0.609	0.95	0.228	***0.94***	***0.068****
COVID-Log	1.11	0.852	1.12	0.777	0.89	0.797	1.22	0.549
**Acceptable WP Org. Risk**								
Country Income Level [a]	1.2	0.830	1.30	0.657	1.33	0.736	1.06	0.926
**Gini Coefficient** [b]	0.97	0.487	***0.95***	***0.093****	0.94	0.230	***0.93***	***0.056****
COVID-Log	0.77	0.617	**0.44**	**0.034***	0.42	0.457	0.98	0.951
**Adequate IPC mitigation**								
Country Income Level [a]	**9.62**	**0.041***	**11.25**	**0.004***	**36.89**	**0.001***	**6.92**	**0.001***
**Gini Coefficient** [b]	0.94	0.223	0.95	0.220	0.98	0.647	**0.95**	**0.047***
COVID-Log	0.54	0.433	0.69	0.479	0.48	0.190	0.70	0.264
**Adequate OHS mitigation**								
Country Income Level [a]	3.00	0.153	**3.94**	**0.047***	**5.46**	**0.020***	**18.39**	**<0.001***
**Gini Coefficient** [b]	***0.94***	***0.077****	**0.92**	**0.020***	***0.94***	***0.061****	0.99	0.755
COVID-Log	0.67	0.392	0.79	0.556	0.56	0.152	0.99	0.988

[a] Country Income was coded as comparing “High and Upper-Middle Income” countries versus “Low and Lower-Middle Income” countries

[b] Gini coefficient was considered in the logistic regression analysis as a continuous variable;

[c] COVID levels where the log value of the rate of cases per million at the beginning of the survey (taken June 1, 2020); log values to smooth very high levels while taking variation into account

[d] Odds Ratios were calculated by assessing the likelihood (OR) of the presence of a mean scores ≥ 5 corresponding to assessments that mitigation “exists and offers some protection” or better; or level of risk is assessed as “risk is acceptable for a short time” or better.

[e] includes all patient care and workplace specialist support personnel such as OHS and IPC professionals

* Indicates statistical significance ($p<0.05$) and also bolded; ** $p<.10$ but $>.05$; indicated in italics and bold

For peer review only

BMJ Open

Impacts of economic inequality on healthcare worker safety at the onset of the COVID-19 pandemic: Cross-sectional analysis of a global survey

Journal:	*BMJ Open*
Manuscript ID	bmjopen-2022-064804.R1
Article Type:	Original research
Date Submitted by the Author:	30-Aug-2022
Complete List of Authors:	Harrigan, Sean; The University of British Columbia School of Population and Public Health, Global Health Research Program<br>Tsang, Vivian; The University of British Columbia, Faculty of Medicine<br>Yassi, Annalee ; The University of British Columbia School of Population and Public Health, Global Health Research Program<br>Zungu, Muzimkhulu ; National Institute for Occupational Health; University of Pretoria Faculty of Health Sciences, School of Health Systems and Public Health<br>Spiegel, Jerry; The University of British Columbia School of Population and Public Health, Global Health Research Program
**Primary Subject Heading**:	Occupational and environmental medicine
Secondary Subject Heading:	Occupational and environmental medicine, Global health, Health services research, Public health, Infectious diseases
Keywords:	COVID-19, Public health < INFECTIOUS DISEASES, Health & safety < HEALTH SERVICES ADMINISTRATION & MANAGEMENT, Human resource management < HEALTH SERVICES ADMINISTRATION & MANAGEMENT, OCCUPATIONAL & INDUSTRIAL MEDICINE

SCHOLARONE™
Manuscripts

I, the Submitting Author has the right to grant and does grant on behalf of all authors of the Work (as defined in the below author licence), an exclusive licence and/or a non-exclusive licence for contributions from authors who are: i) UK Crown employees; ii) where BMJ has agreed a CC-BY licence shall apply, and/or iii) in accordance with the terms applicable for US Federal Government officers or employees acting as part of their official duties; on a worldwide, perpetual, irrevocable, royalty-free basis to BMJ Publishing Group Ltd ("BMJ") its licensees and where the relevant Journal is co-owned by BMJ to the co-owners of the Journal, to publish the Work in this journal and any other BMJ products and to exploit all rights, as set out in our licence.

The Submitting Author accepts and understands that any supply made under these terms is made by BMJ to the Submitting Author unless you are acting as an employee on behalf of your employer or a postgraduate student of an affiliated institution which is paying any applicable article publishing charge ("APC") for Open Access articles. Where the Submitting Author wishes to make the Work available on an Open Access basis (and intends to pay the relevant APC), the terms of reuse of such Open Access shall be governed by a Creative Commons licence – details of these licences and which Creative Commons licence will apply to this Work are set out in our licence referred to above.

Other than as permitted in any relevant BMJ Author's Self Archiving Policies, I confirm this Work has not been accepted for publication elsewhere, is not being considered for publication elsewhere and does not duplicate material already published. I confirm all authors consent to publication of this Work and authorise the granting of this licence.

For peer review only

Impacts of economic inequality on healthcare worker safety at the onset of the COVID-19 pandemic: Cross-sectional analysis of a global survey

Sean P. Harrigan[1,2], Vivian W. L. Tsang[1,3], Annalee Yassi[1], Muzimkhulu Zungu[4,5], Jerry M. Spiegel[1*]

[1] Global Health Research Program, School of Population and Public Health, Faculty of Medicine, University of British Columbia, Vancouver BC, Canada

[2] Master of Science in Population and Public Health, School of Population and Public Health, Faculty of Medicine, University of British Columbia, Vancouver BC, Canada

[3] Undergraduate Medical Program, Faculty of Medicine, University of British Columbia, Vancouver BC, Canada

[4] National Institute for Occupational Health (NIOH), a division of National Health Laboratory Service (NHLS), Johannesburg, South Africa

[5] School of Health Systems and Public Health, University of Pretoria, Pretoria, South Africa

*Corresponding author

E-mail: jerry.spiegel@ubc.ca (JMS)

Jerry M. Spiegel

Professor, School of Population & Public Health, Faculty of Medicine
University of British Columbia
430-2206 East Mall, Vancouver, BC V6T 1Z3 CANADA

Word Count: 4858 words Abstract: 298 words

For peer review only

For peer review only - http://bmjopen.bmj.com/site/about/guidelines.xhtml

ABSTRACT

Objectives To assess the extent to which protection of healthcare workers (HCWs) as COVID-19 emerged was associated with economic inequality among and within countries.

Design Cross-sectional analysis of associations of perceptions of workplace risk acceptability and mitigation measure adequacy with indicators of respondents' respective country's economic income level (World Bank assessment) and degree of within-country inequality (Gini index).

Setting A global self-administered online survey.

Participants 4,977 HCWs and healthcare delivery stakeholders from 161 countries responded to health and safety risk questions and a subset of 4,076 (81.2%) answered mitigation measure questions. The majority (65%) of study participants were female.

Results While the levels of *risk* being experienced at the pandemic's onset were consistently deemed as unacceptable across all groupings, participants from countries with less income inequality were somewhat less likely to report unacceptable levels of risk to HCWs regarding both workplace environment (OR=0.92, p=0.012) and workplace organizational factors (OR=0.93, p=0.017) compared to counterparts in more unequal national settings. In contrast, considerable variation existed in the degree to which *mitigation* measures were considered adequate. Adjusting for other influences through a logistic regression analysis, respondents from lower-middle and low-income countries were comparatively much more likely to assess both occupational health and safety (OR=10.91, p=<0.001) and infection prevention and control [IPC] (OR=6.61, p=<0.001) protection measures as inadequate, despite much higher COVID-19 rates in wealthier countries at the time of the survey. Greater within-country income inequality was also associated with perceptions of less adequate IPC measures (OR=0.94, p=0.025). These associations remained significant when accounting for country-level differences in occupational and gender composition of respondents, including specifically when only female care providers, our study's largest and most at-risk sub-population, were examined.

Conclusions Economic inequality threatens resilience of health systems that rely on health workers working safely to provide needed care during emerging pandemics.

For peer review only

Strengths and limitations of this study

- A major strength of the study is its novel empirical testing of the "income inequality" hypothesis for a comparative cross-country analysis of a major global health challenge: protection for a workforce central to the provision of healthcare services during a pandemic.
- This study is based on a unique global self-administered online survey conducted by a network of occupational health experts coordinated by the World Health Organization (WHO) through a large array of professional networks and social media.
- A major limitation of the study is its character as a convenience sample with different compositions by gender and occupation among countries and small sample sizes in some countries; however access to gender and occupation identifiers of respondents has enabled adaptive strategies to take this into consideration.
- The study is exploratory in considering associations with economic inequality, but does not provide a way to consider pathways for this effect, so further research will be needed for this.

Keywords: COVID-19; Health Systems; Public Health; Other Study Design; Environmental Health Occupational Health; Healthcare Workers

For peer review only

INTRODUCTION

As the COVID-19 pandemic emerged, attention was quickly drawn to risks faced by frontline healthcare providers [1,2] – and the urgent need to strengthen their protection [3,4]. By September 2020, it was estimated that 10% of global infections had been in health workers (HCWs), and over 7,000 had died [5,6]. Notwithstanding inconsistent reporting, Papoutsi and colleagues, in reviewing the global burden of COVID-19 for HCWs by country [6, 7], estimated the percentage of HCW cases among the total cases by April 2020 as ranging from less than 1% in Hong Kong and India, to 19% in Spain.

Despite 60 million people employed in the healthcare sector worldwide [8,9], a global shortage of HCWs persists and is especially critical in low-and middle-income countries (LMICs) [10], where the greatest global burden of disease exists [11]. Risk mitigation is marked by considerable variation [12], with shortcomings in infrastructure and mitigation programs contributing to higher burdens of disease and HCW risk in more poorly resourced settings [13]. The danger that HCWs face of acquiring COVID-19 adds to extensive existing risks in infectious disease endemic states, for example with tuberculosis in Sub-Saharan Africa [14].

While lack of personal protective equipment (PPE) was highlighted early in the COVID-19 pandemic, consideration of broader OHS factors and mitigation measures attracted less initial attention [7,8,15]. To ascertain the extent of OHS risk exposure and the adequacy of mitigation measures in place to meet the challenge of COVID-19, an Ad Hoc expert group of the WHO, the International Labour Organization (ILO) and the International Commission on Occupational Health (ICOH) prepared and circulated a questionnaire survey to identify HCWs perceptions of

For peer review only - http://bmjopen.bmj.com/site/about/guidelines.xhtml

the most common threats to their health and safety as well as the adequacy of mitigation measures in the emerging pandemic [16].

Further to a preliminary analysis of survey results [16] regarding risk and adequacy of protection, we sought to ascertain the degree to which perceived risk exposure of HCWs and adequacy of mitigation measures is associated with a country's economic characteristics. Considerable attention, after all, has been given to the impact of economic disparity on health [17,18], especially in relation to Wilkinson's "economic inequality hypothesis" suggesting that greater inequality is associated with poorer health [19]. In recognition that "the traditional exposure-disease framework used in occupational health research is not equipped to address societal contexts in which work is embedded" [20], we sought to examine how such driving forces [21]as a country's economic inequality might be affecting the wellbeing of HCWs .

A variety of factors have been examined that might have influenced how the onset of the COVID-19 pandemic was experienced in different national settings, including consideration of cultural traits [22], specific government regulations and non-pharmaceutical interventions [23,24], and political leadership characteristics [25]. Our study sought to apply a cross-country perspective to consider the effects of economic inequality, recognizing this to be a dimension of considerable relevance in global public health research.

With this focus, we set out to first consider variation in perceptions of the acceptability of work-related risks and the adequacy of mitigation measures that were being experienced by HCWs as COVID-19 emerged; and, second, to determine the extent to which variations were associated with a country's comparative income level and degree of income inequality.

For peer review only

For peer review only - http://bmjopen.bmj.com/site/about/guidelines.xhtml

METHODS

Survey development

Shortly after the *WHO Ad Hoc Study Group on Health and Safety of Health Workers* was established when the COVID-19 pandemic emerged, it created an online survey aimed at HCWs from all WHO regions globally. In addition to the capture of demographic indicators of respondents, the survey contained 41 questions – 17 on health and safety risks and 24 on mitigation measures [16, Appendix]. Risk questions were grouped into those related to infectious disease transmission, physical work environment, psychological work environment and work organization. For each risk question, participants were asked *"Think about the working conditions of health workers in your country, jurisdiction or health facility….; rate the current level of these risks, now during the COVID-19 pandemic*." Questions regarding mitigation measures were divided into two groups: occupational health and safety (OHS) and infection prevention and control (IPC). Here, participants were similarly asked: *"Think about the working conditions of health workers in your country, jurisdiction or health facility…rate the level of application of these measures according to your knowledge of the real situation now during the COVID-19 pandemic."*

Patient and Public Involvement

The participation of health workers (whose wellbeing is the focus for this study in relation to their assessment of the adequacy of measures to protect them) was indirectly included through the participation of their representatives (unions within the ILO and other professional bodies) who were directly involved in the creation of the research instrument and in the dissemination of the online survey and its initial results.

For peer review only

For peer review only - http://bmjopen.bmj.com/site/about/guidelines.xhtml

Study population and inclusion criteria

Participants were recruited by convenience sampling, with dissemination through a large array of professional networks and social media. The survey, self-administered online to enable rapid low-cost recruitment, was available in Arabic, Chinese, English, French, German, Italian, Portuguese, Russian, Spanish and Swahili. A range of HCWs and stakeholders involved with healthcare delivery were invited to participate. In addition to HCWs in direct patient care in both formal and informal settings and in public and private facilities, respondents also included allied health and supporting staff, including OHS and IPC professionals, administration, management, drivers, public health workers, community health workers, and others as defined by the International Standard Classification of Occupations (ISCO-08). Data collection occurred between May 5th and June 25th, 2020. Participant results were excluded if they failed to complete demographic questions or if they failed to provide any responses to the risk and mitigation questions. As the survey was designed to be completed and submitted anonymously, no formal request for signed consent was solicited, with participants' submission itself indicating consent to use the information provided as anonymized aggregated data. The study proposal was approved by the Behavioural Research Ethics Board of the University of British Columbia (Ref. H20-01825). This work was supported by the International Development Research Centre (IDRC) under grant M20-00559 and the Canadian Institutes of Health Research (CIHR) under grant VS1-175519 for the “Protecting healthcare workers from COVID-19: a comparative contextualized analysis” research programme.

For peer review only

Independent variables

Demographic information for individual survey respondents was collected on country, gender, and occupation – the latter separated into 13 categories and then grouped into patient care/health services; specialized technical support; clerical support/administration and management; and other. Details about the study sample population composition and demographic characteristics of participants are presented in Figure S1 and Table S1 respectively.

Our research group, drawn from two WHO Collaborating Centres participating in the survey process, conducted the analysis by consolidating respondents by their home country and then linking this to a WHO geographic region [26]; a comparative country-level economic classification by World Bank income groups based on the annual Atlas gross national income per capita estimates [26,27]; and the country's Gini index - a measure used for the analysis of income inequality present within a country, with a score of 0 representing perfect equality, and a score of 1 representing complete inequality [28]. Data for Gini and economic classification were taken from the World Bank, using the most recent data available. To take account of the variation across regions present during the initial phase of the pandemic, we also considered COVID-19 incidence per million (logarithmic scale) in each country at the time when the survey was completed, as an indicator of the intensity as of a particular date, using values for June 2020 drawn from the "Our World in Data" database [29].

Dependent variables

Acceptability of workplace risks and adequacy of mitigation measures – the dependent variables in this study – were derived from a factor analysis of individual survey responses, then

For peer review only - http://bmjopen.bmj.com/site/about/guidelines.xhtml

aggregated to enable subsequent analysis of the effect of country-level characteristics. Factor analysis [16] was used to reduce the 41 survey questions into coherent groupings and principal component analysis with varimax rotation carried out to create factors from each set of workplace health and safety risk exposure questions (corresponding to workplace risk and workplace organization acceptability) and mitigation measure questions (corresponding to IPC and OHS adequacy); Table S2 summarizes the subject matter covered by the questions consolidated in each factor. Separate factor analyses were run on risk questions and preventive measure questions. Missing values were excluded in a listwise fashion. The rotated component matrix was used to identify factors. To measure scale reliability, Cronbach's Alpha was used for each individual factor. Scores over 0.7 are considered to be acceptable for internal consistency [30]. The results from the factor analysis are outlined further in our preliminary analysis [16].

The questions were administered as a 3-point Likert scale, then converted to a 10-point scale for clearer communication (i.e. midpoint of 2 becoming 5). Numerical scores were assigned to each answer to establish a scale for both the risk and mitigation measure factors, with higher scores corresponding to more desirable states. For health and safety risks, a score of 0 was assigned to "risk is not acceptable at all"; 5 to "risk is acceptable for a short time"; and 10 for "risk is negligible". For mitigation measures, a score of 0 was assigned to "does not exist at all"; 5 to "exists and offers some protection"; and 10 to "exists and offers full protection". Responses of "don't know/unsure" were assigned blanks. Factor scores were then calculated to form an individual respondent's factor score for each of the four groupings, i.e., work environment risk acceptability, work organization risk acceptability, OHS adequacy and IPC adequacy and then aggregated to generate a mean value for each country's respondents, so that inter-country

For peer review only - http://bmjopen.bmj.com/site/about/guidelines.xhtml

comparison could be conducted. The higher the scores, the greater the perceived adequacy of mitigation measures or acceptability of risk deemed as being experienced.

Analysis

The mean country dependent variable factor scores derived from the aggregation of individual participants' responses served as the basis for considering associations by WHO region, economic classification, Gini coefficients and COVID-19 incidence. Comparisons of survey mean scores were carried out using ANOVA analysis, with an alpha of 0.05 used to test significance. To compare means for the continuous variable Gini coefficient and COVID incidence scores, we ordinally divided groups of countries into quartiles by values.

To ensure that intercountry variation was not purely explained by possible gender and occupational compositional differences among a particular country's respondents, we carefully examined possible sources of discrepancy (Table S3), using ANOVA analysis to consider effects that could complicate the cross-country comparison of all respondents. To minimize any such effect, we considered different ways to stratify our analysis of the study population, notably by focusing only on those populations that had the most direct workplace experience to personally being "at risk". Noting the presence of gender differences among patient care deliverers, we specifically isolated female respondents, who in fact constituted the largest demographic group of respondents in the study, representing 1,998 respondents from 112 countries (n=1,968 from 112 countries), the largest sub-population.

For peer review only - http://bmjopen.bmj.com/site/about/guidelines.xhtml

241 Finally, to measure the effect that the interaction of independent variables had on the likelihood
242 of workplace risks being considered as acceptable in a country setting as COVID was emerging,
243 and workplace protection and control measures being deemed as adequate, we created and
244 applied a logistic regression model. Preferred outcomes for this analysis were assessed as mean
245 factor scores ≥ 5, corresponding to assessments that mitigation "exists and offers some
246 protection" or better; or "risk is acceptable for a short time" or better. All statistical analysis was
247 done using R and SPSS Statistics software [31,32].

248 RESULTS

249 Overall study population and survey responses

250 There were 4,977 participants who responded to health and safety risk questions and a subset of
251 4,076 (81.2%) who answered mitigation measure questions. The majority of study participants
252 were female (65%), reflecting the make-up of the health sector workforce. Most participants
253 were from the European region (35%), followed by the Americas (31%), the Western Pacific
254 region (15%) and Africa (10%); the South East Asian (4%) and Eastern Mediterranean regions
255 (3%) made up the smallest proportion of participants. In total there were 161 countries
256 represented in the survey. Portugal (n=549, 11%), US (n=451, 9%), Brazil (n=373, 7%), Canada
257 (n=263, 5%), and China (n=233, 5%) had the most participants. The majority of respondents
258 were from countries of high-economic classification (59%), followed by upper-middle (27%),
259 lower middle (10%) and low (4%). Most survey participants worked for a health services
260 employer (61%), followed by government services (15%) and businesses and farms (10%).
261 Those working in academia, professional associations, international organizations and non-
262 government organizations each encompassed less than 10%. Finally, the type of occupation was
263 predominantly patient care/health (56%) services, followed by 29% providing technical services

For peer review only

such as IPC or OHS specialists, 7% in administration and 10% identified as working in other sectors (Table S1).

The largest percentage of countries were in Europe (30%) and over a third of all countries were high-income countries (35%). The average Gini index was 37.8 (SD=7.7) and the mean and median COVID-19 incidence rate per million was 1,360 and 278, respectively at the time the survey was conducted.

Table 1 illustrates that considerable variation exists in these variables across the different WHO regions, indicating the distinct characteristics and conditions present at the onset of the pandemic. It is especially noteworthy that case levels had been far greater in high-income country areas at the survey mid-point (June 1st, 2020). For example, the cases per million was 2,525 in Europe versus 119 in Africa; 5,408 in the United States; and only 138 in India and 97 in Indonesia.

Table 1: Country characteristics of different WHO regions

Region	Number of countries	Countries by income classification*				Mean country values		Study Population characteristics	
		High	Upper-middle	Lower-middle	Low	Inequality: Gini coefficient*	COVID: cases per million a,*	Gender: Female* (%)	Occupation: Frontline Patient care* (%)
Overall	161	57	42	36	26	37.8	1,360	65.5%	56.4%
AFRO	37	0	6	12	19	43.2	119	44.8%	52.1%
EMRO	20	6	3	7	4	35.2	2,407	39.8%	29.4%
EURO	48	32	13	2	1	31.8	2,525	68.2%	64.4%
PAHO	30	11	14	4	1	44.8	1,135	73.3%	46.5%
SEARO	9	0	2	6	1	35.0	86	36.7%	56.3%
WPRO	17	8	4	5	0	37.0	512	70.7%	68.8%

Abbreviations: AFRO: Africa; EMRO: Eastern Mediterranean; EURO: Europe; p: p-values; PAHO: Americas; SEARO: South-East Asian; WPRO: Western Pacific
Note: * p <0.001, a: COVID rates as of June 2020.

For peer review only

For peer review only - http://bmjopen.bmj.com/site/about/guidelines.xhtml

As summarized in Table 2 (full table in appendix Table S4), the majority of respondents designated most of the health and safety risk parameters as “not acceptable at all”. Circumstances most reported as such included bullying or psychological harassment in the workplace (54%), physical violence and assaults (54%), exposure to blood, bodily fluids, and other infectious materials (52%), inadequate sanitation facilities (52%), and sexual harassment (50%). In contrast, areas such as time pressure and high workload (38%), skin damage from PPE (33%) and shift work with night shifts (23%) were deemed to be less of a concern. There were no risk categories in which the most common response was “risk is negligible”.

Mitigation measures related to the above areas of concern were seen as particularly lacking, with only the category of “policies for facilities for hand hygiene” designated as “exists and offers full protection” (full tables in appendix Table S5). For example, despite psychosocial-related risks, including bullying, harassment, physical violence, and sexual harassment ranked consistently high (54%, 54%, 50% respectively), only 21% indicated that corresponding policies “exist and offer full protection”, with similar dissatisfaction for the adequacy of mitigation measures for other key areas such as IPC policy (28%), availability of PPE (34%) as well as training and education of workers about OHS (21%) and IPC (32%). Only in two mitigation measures areas – availability of facilities for hand hygiene, and policies for post-exposure prophylaxis (such as HIV or hepatitis B) – did most participants indicate that measures existed and offered full protection (54% and 42%, respectively). These results show an overwhelming majority of participants indicating that the risks they faced were not acceptable at all and that very few of the corresponding mitigation measures offered adequate protection to HCWs.

For peer review only - http://bmjopen.bmj.com/site/about/guidelines.xhtml

Table 2: Risk acceptability and mitigation adequacy – selected worldwide survey responses

Risk acceptability	Risk is not acceptable at all	Risk is acceptable for a short time	Risk is negligible	Don't know/unsure
**Infectious risk work environment**				
Exposure to blood, body fluids, respiratory secretions, other potentially infectious materials	**52%**	29%	15%	4%
Inadequate sanitation facilities	**52%**	21%	23%	4%
Skin damage from personal protective equipment and/or frequent hand hygiene	33%	**46%**	16%	5%
**Physical work environment**				
Crowded workplace	**42%**	36%	18%	4%
Thermal discomfort (cold, heat, humidity)	25%	**46%**	24%	5%
**Psychosocial work environment**				
Bullying or psychological harassment	**54%**	18%	21%	7%
Sexual harassment	**50%**	10%	31%	9%
**Work organization**				
Time pressure, high workload	38%	**49%**	10%	3%
Shift work with night shifts	23%	**48%**	21%	8%

Mitigation measure adequacy	Does not exist at all	Exists and offers some protection	Exists and offers full protection	Don't know/ unsure
**Infection prevention and control**				
IPC policy in the health facility	8%	**60%**	28%	4%
Personal protective equipment, e.g. masks, gloves, goggles, gowns are readily available	8%	**55%**	34%	3%
Training and education of workers about infection prevention and control	11%	**54%**	32%	3%
Facilities for hand hygiene (hand washing and disinfection) are readily available	3%	40%	**54%**	3%
**Occupational health and safety**				
Occupational health and safety policy and management system in the facility	14%	**58%**	22%	6%
Regular assessment of workplace health and safety risks and controls	22%	**51%**	21%	6%
Engineering controls, such as ventilation, physical barriers, safer devices	19%	**54%**	19%	8%
Prevention of workplace violence and security measures	21%	**52%**	21%	6%
Workplace policies against bullying, psychological and sexual harassment	27%	**43%**	21%	9%

Note: Most cited response highlighted in bold

For peer review only

Associations with risk exposure acceptability and mitigation measure adequacy

Unacceptable levels of *risk* (i.e., factor scores below 5) were consistently reported for both Work Organization and Work Environment across geographic regions, economic income level categories, equity classifications and COVID-19 incidence rates, with no statistically significant differences observed within these categories (Table 3). However, we observed multiple significant differences in how the adequacy of OHS and especially IPC (overall mean of 4.67) *mitigation measures* were perceived. These apparent associations, observed to be present for all the explanatory factors we examined, drew attention to the need to consider the adjusted effect of each independent variable through the logistic regression analysis that we subsequently conducted.

For peer review only

Table 3:Unadjusted risk acceptability and Mitigation adequacy associations

Explanatory Variable		Risk acceptability: Work environment mean	p	Risk acceptability: Work organization mean	p	Mitigation adequacy: IPC mean	p	Mitigation adequacy: OHS mean	P
**TOTAL**	By country means	4.23		4.29		4.67		6.08	
	By individuals	3.88		3.87		4.79		6.28	
**Region**	AFRO	4.11	0.34	4.17	0.30	**3.68**	**<0.01***	**5.31**	**0.03***
	EMRO	4.01		4.25		**5.02**		**6.33**	
	EURO	4.47		4.24		**5.28**		**6.54**	
	PAHO	4.03		3.99		**4.24**		**5.92**	
	SEARO	3.44		4.76		**5.11**		**6.30**	
	WPRO	4.83		5.03		**5.24**		**6.35**	
**Economic Classification**	High	4.51	0.24	4.62	0.15	**5.61**	**<0.01***	**6.99**	**<0.01***
	Upper-middle	4.05		4.05		**4.85**		**6.17**	
	Lower-middle	3.78		4.05		**3.58**		**5.15**	
	Low	4.51		4.27		**3.88**		**5.29**	
**Gini coefficient**	Q1 [lowest]	4.80	0.11	4.51	0.34	**5.26**	**0.01***	**6.64**	**0.04**
	Q2	4.10		4.29		**4.31**		**5.81**	
	Q3	3.90		4.04		**4.72**		**6.20**	
	Q4	3.98		3.80		**3.89**		**5.55**	
**COVID-19 incidence rate**	Q1 [lowest]	3.95	0.50	4.09	0.84	**4.17**	**<0.01***	5.64	0.07
	Q2	4.18		4.25		**4.39**		5.95	
	Q3	4.50		4.39		**4.66**		6.10	
	Q4	4.19		4.16		**5.44**		6.62	

Abbreviations: AFRO: Africa; EMRO: Eastern Mediterranean; EURO: Europe; IPC: Infection protection and control; OHS: Occupational health and safety; p: p-values; PAHO: Americas; SEARO: South-East Asian; Q: Quartile; WPRO: Western Pacific

* Indicates statistical significance (p<0.05) of differences among the means of country mean values for category; significant values in bold

For peer review only

For peer review only - http://bmjopen.bmj.com/site/about/guidelines.xhtml

To understand potential sources of difference that could be attributed to heterogeneous composition of country responses that is encountered in conducting a cross-country comparison such as the one we conducted, Table 4 presents a summary of the survey's individual level data to indicate how gender and occupation were associated with respondent perceptions of acceptability and adequacy. Females were somewhat more likely than males to report workplace risks being unacceptable (3.76 versus 4.11; $p<0.001$), but the strong presence of frontline patient care providers in the gendered health workforce was largely responsible for this, as no statistically significant differences were observed within other occupation groupings (see Table S3). In fact, patient care providers themselves stood out as being the occupational grouping most critical of workplace risk acceptability as well as OHS and IPC measure adequacy. In contrast, male administrators/managers stood out as the most likely to indicate that acceptable risk exposure and adequate risk mitigation measures were present. This discrepancy is understandable as frontline workers, and women in this occupation grouping, represent those most directly experiencing the impact of the COVID pandemic. However, even in these more extreme circumstances where differences were observed, the comparative differences in mean scores (that were then aggregated in calculating country mean values) were not large. Moreover, the fact that the African region, where strongest concerns about unacceptable risk and inadequate mitigation were expressed, actually had proportionately fewer female respondents, indicates that even these regional concerns that we observed may well have been underrepresented in this unadjusted analysis.

For peer review only - http://bmjopen.bmj.com/site/about/guidelines.xhtml

Table 4: Risk acceptability and mitigation adequacy associations [a] with gender and occupation

Explanatory Variable		n[d]	Risk acceptability: Work environment mean	p	Risk acceptability: Work organization mean	P	Mitigation adequacy: IPC mean	p	Mitigation adequacy: OHS mean	P
**Gender[b]**	Total	4863	3.88	**<0.01***	3.87	0.40	4.79	0.09	6.28	0.07
	Female	3220	**3.76**		3.85		4.74		6.33	
	Male	1643	**4.11**		3.92		4.88		6.19	
**Occupation[c]**	Total	4916	3.88	**0.04**	3.87	0.10	4.79	**<0.01***	6.28	0.19
	Patient Care	2792	**3.91**		3.88		**4.63**		6.27	
	Specialist	1404	**3.84**		3.80		**4.90**		6.30	
	Admin-Mgr	327	**4.14**		4.22		**5.50**		6.50	
	Other	393	**3.55**		3.82		**5.03**		6.08	

* Indicates statistical significance ($p<0.05$); significant values in bold

[a] This table report on total respondents in each category, without any consideration for different mixes of gender within different occupations, and different mixes of occupation within genders; Supplementary table X provides results with full occupation and gender breakdowns

[b] Only respondents indicating Male or Female were included in exploring differences;

[c] Occupation was initially coded with finer detail but then consolidated in these composites for comparative analysis

[d] total n varies by specific factor; this column refers to n for workplace environment, where response was greatest

Abbreviations: IPC: Infection protection and control; Mgr: Manager ; OHS: Occupational health and safety

Influence of between-country and within-country income disparity

Table 5 summarizes the adjusted comparative effects of income level and income distribution disparity in each country setting while taking into consideration potential influences prompted by differing COVID-19 rates in the initial phase of the pandemic. While there was no difference between *higher and lower income countries* regarding the perception of unacceptable levels of *risks* in healthcare workplaces in all settings, *within-country inequality* was associated with a mildly increased likelihood of *unacceptable levels of risk* with regard to both workplace environment (OR=0.92; p=0.012) and workplace organizational (OR=0.93; p=0.017) factors.

For peer review only

For peer review only - http://bmjopen.bmj.com/site/about/guidelines.xhtml

Table 5: Factors associated with perceived risk acceptability and mitigation adequacy

	Unadjusted bivariate			Adjusted multivariable model [d]			
**Explanatory Variable** (organized by outcome area)	**OR[d]**	**95% CI**	**p**	**B**	**OR[d]**	**95% CI**	**P**
**Acceptable WP Enviro. Risk**							
Country Income Level [a]	1.56	1.08-2.27	0.231	0.215	1.24	0.70-2.20	0.708
**Gini Coefficient [b]**	**0.91**	**0.88-0.94**	**0.005***	**-0.087**	**0.92**	**0.89-0.95**	**0.012***
COVID-Log[c]	1.01	0.83-1.23	0.965	0.083	1.09	0.78-1.51	0.801
**Acceptable WP Org. Risk**							
Country Income Level [a]	0.83	0.59-1.17	0.587	-0.341	0.71	0.42-1.21	0.52
**Gini Coefficient [b]**	**0.94**	**0.91-0.97**	**0.028***	**-0.076**	**0.93**	**0.90-0.96**	**0.017***
COVID-Log[c]	0.8	0.67-0.97	0.243	-0.113	0.89	0.66-1.21	0.710
**Adequate IPC mitigation**							
**Country Income Level [a]**	**6.8**	**1.36-34.60**	**0.006***	**1.889**	**6.61**	**3.68-11.88**	**0.001***
**Gini Coefficient [b]**	**0.93**	**0.90-0.95**	**0.006***	**-0.036**	**0.94**	**0.91-0.96**	**0.025***
**COVID-Log[c]**	**1.85**	**1.51-2.26**	**0.002***	-0.064	0.76	0.55-1.03	0.373
**Adequate OHS mitigation**							
**Country Income Level [a]**	**8.91**	**5.76-13.80**	**<0.001***	**2.389**	**10.91**	**5.63-21.12**	**<0.001***
Gini Coefficient [b]	0.97	0.94-0.99	0.183	-0.009	0.99	0.96-1.02	0.779
**COVID-Log[c]**	**0.94**	**0.91-0.97**	**0.028***	0.079	1.08	0.77-1.52	0.816

Notes: OR: Odds Ratio, expressed as Exp(B) value in Logistic Regression analysis, CI: Confidence Interval, * p ≤ .05 ** <001
Abbreviations: B: Coefficient; OHS: Occupational Health and Safety mitigation; Org.: organization; IPC: Infection Prevention and control; Enviro.: environmental; WP: workplace
Variables where statistical significance is present are shown in bold
[a] Country Income was coded as comparing "High and Upper-Middle Income" countries versus "Low and Lower-Middle Income" countries
[b] Gini coefficient was considered in the logistic regression analysis as a continuous variable;
[c] COVID levels where the log value of the rate of cases per million at the beginning of the survey (taken June 1, 2020); log values to smooth very high levels while taking variation into account
[d] Odds Ratios were calculated by assessing the likelihood (OR) of the presence of a mean score ≥ 5 corresponding to assessments that mitigation "exists and offers some protection" or better; or level of risk is assessed as "risk is acceptable for a short time" or better.
* Indicates statistical significance (p<0.05); also bolded

As was observed in unadjusted bivariate analyses, there was much stronger divergence in perceptions of acceptable *mitigation measures* by both *country income level* and *income inequality*, with an almost 7-fold greater likelihood of IPC measures (OR=6.61; p=0.001) being considered adequate in wealthier countries, and over a ten-fold difference in adequacy of OHS

For peer review only

For peer review only - http://bmjopen.bmj.com/site/about/guidelines.xhtml

measures (OR=10.91; p<0.001), despite the greater intensity of COVID-19 in wealthier countries at the time of the survey. In fact, the counter-intuitive positive association that seemed to be present between intensity of COVID-19 and perceptions of adequacy disappeared in our adjusted multivariable analysis. And further to the observed unadjusted effect, higher inequality decreased the likelihood (OR=0.94; p=0.025) of deeming IPC measures to be adequate.

Analysis of the more homogeneously constituted population of female patient care provider respondents (Table S6) further revealed that this group's more critical assessment of risk that we had documented in Table 4 especially influenced perceptions of risk acceptability in settings where COVID-19 exposure had intensified. In this regard, workplace organizational factors, which included consideration of the workload being encountered, were substantially more likely to be seen as unacceptable (OR=0.44; p=0.034) by female patient care providers in countries with higher COVID presence; a perception reinforced by a further (albeit less pronounced) effect of in-country income inequality (OR=0.95; p=0.093).

As we had observed was the case for all respondents, female care providers in higher income countries were more likely to perceive mitigation measures to be adequate (OHS OR=3.94; p=0.047 and IPC OR=11.25; p=0.004) than those in more poorly resourced settings, and this was further accompanied by an effect of within-country inequality also contributing some explanatory power (OHS OR=0.92; p=0.020).

For peer review only - http://bmjopen.bmj.com/site/about/guidelines.xhtml

DISCUSSION

High levels of concern about emerging threats to HCWs were widely published in the first year of the COVID-19 pandemic, providing extensive evidence about morbidity and mortality associated with healthcare work [33–35] as well as effects on job satisfaction [36]. Although meta-analyses have been conducted to synthesize such findings [37], our article provides one of the first worldwide examinations of contextual factors affecting the wellbeing of HCWs during the COVID-19 pandemic, enabling a comparative cross-country analysis. In doing so, it notably complements studies calling attention to inadequate implementation of OHS and IPC measures, for example in South Africa [38] as well as a need to consider the influence of structural determinants that affect how risks are experienced in specific health worker exposure contexts [39]. The results presented here contribute a theoretical and empirically-based understanding of the importance of inequality among and within countries in this regard. This has implications for preparedness for any future pandemic outbreaks.

Our findings clearly demonstrate that there is a strong need for improvements in OHS for HCWs not only to protect against infectious disease transmission but to also control the threat of psychosocial risks, a consideration that resonates with studies highlighting effects on mental health of HCWs as already stressed workplaces with intensifying pressures when pandemics emerge [40–44]. Widespread concerns about health risks identified in diverse locations such as Ethiopia, Turkey, Italy and Spain in many facets of health work [45–48] signal a strong rationale for international collaboration in seeking effective technical and policy approaches to best protect HCWs.

For peer review only - http://bmjopen.bmj.com/site/about/guidelines.xhtml

Despite a common assessment of unacceptable levels of risk everywhere, our study revealed important differences in the perceived adequacy of protective measures to meet this challenge. Such results point to the need to add explicit attention to OHS measures in the World Health Organization's call for better planning healthcare human resources [10] as well as the updating of the WHO's *Global Plan of Action for Occupational Health*, considering what this means for HCWs in light of the COVID-19 experience.

While the case prevalence in any one single country clearly influences the intensity of possible healthcare workplace exposure as a global pandemic emerges, HCWs in all countries face the same need for proper PPE, appropriate testing and vaccines as they compete in the same markets and the same supply chains [49,50]. While there is now appropriate attention focused on the need to address global inequities in vaccine accessibility [51], our study highlights other inequities that also call for greater attention. Moreover, our analysis stands out by considering how variation in protecting HCWs may be associated with the presence of contextual social and economic inequities, itself an important social determinant of health that has been prominent in global health research literature. What is of particular relevance here is the vulnerability of HCWs as "canaries" in a workplace made vulnerable by the emergence of a novel infectious disease [52], where preparedness to meet a new challenge is critical.

While the presence of unacceptable risk was clearly identified in all countries, it was striking that the strongest concern about inadequate protection of HCWs came not from the HICs hit most intensely by the initial wave of COVID in early 2020, but rather less well-resourced settings that had yet to be as strongly affected. This vividly echoes pre-COVID findings that resource-poor

For peer review only

countries have decreased capacities for protecting HCWs [13,14] even beyond needs for testing and contact tracing, and consistent with studies noting needs for training and PPE for HCWs [53]. This furthermore mirrors experience in previous pandemics such as Ebola in West Africa where meaningful investments in PPE were shown to be important elements in combatting the spread of disease [54], a matter that is now being observed with regard to COVID-19 [55]. Our finding that country income level is strongly associated with greater capacity to provide prevention and mitigation within a health system is thus not surprising.

Previous literature on the effects of income inequality within a society has however been less conclusive, at times contesting the implications of the Wilkinson's "economic inequality hypothesis". In this regard, Blázquez-Fernández and colleagues concluded that income inequality does not significantly reduce health in 'developed' societies [56] and Mellor and Milyo further argued that there is little support for relation between income inequality and individual or population health after fixed division effects were included [57]. However, when attention is paid to methodological concerns [17], strong evidence of the effect of economic inequality has been observed in Sub-Saharan African countries [58]. Looking beyond levels of economic indicators alone, a systematic study of "welfare regimes" (i.e. characterizations of policy orientations dominant in a country at a particular time) has suggested that precarious workers fare better in the context of "Scandinavian state" policies [59]. Indeed countries that recognized COVID-19 as a work-related disease and supported workers with compensation and appropriate absence policies, were reported to have reduced mental health stressors, pointing to opportunities for improving HCW well-being [60]. However, a systematic review of the impact

For peer review only

of political economy on health observed substantial gaps in knowledge, calling for "higher-quality reviews and empirical studies in this area" [61].

Our study suggests that societies with greater national income equality may well be characterized by policies that are more protective of vulnerable populations such as HCWs, a group whose comparatively high occupational health risk is aggravated by the onset of pandemics. To better understand the pathways and iterative relationships that can explain this, case study examinations would certainly be of value. Moreover, with health worker protection so strategically important to health system functioning during such crises that threaten global health equity, countries known to be highly unequal might accordingly be deemed to be in need of even further technical assistance and attention to ensure that adequate protection is provided to HCWs at risk.

Recognizing that appreciation of the contribution of HCWs soared as the COVID pandemic advanced, our observations that economic inequality among and within countries is associated with the degree to which HCWs face unacceptable risk and inadequate protection signals a vital need to promote social justice for those who play such an important role in the care of populations *before* a new pandemic emerges. In light of this, from an analytical perspective, we strongly endorse the call for a new paradigm [62] to better understand how upstream and socio-political factors could be "affecting the nature of work and employment and their impact on the health of workers, the public, and the planet" [63]. This includes consideration of international cooperation not only with respect to vaccine supply, but also to ensure that less wealthy countries receive technical assistance in establishing protection and mitigation programs as well as

For peer review only

attention to pathways sensitive to the offloading of risks to more marginalized worker populations.

Limitations and further research needs

Cross-country comparative studies such as ours rely on a convenience sample, leading to some countries being over-represented while others were under-represented or non-existent. To address possible concerns about the influence of countries with low respondent counts, we examined this concern by conducting sensitivity analyses, summarized in Table S7, to consider possible implications, but concluded that this did not warrant a questioning of our findings. Additionally, the classification of countries purely by national income levels leads to designating some countries as high income in settings where national institutions may be minimally developed despite high levels of income earned through high value exports such as petroleum or in settings of small populations with externally controlled tourism sectors. As such, we developed grouping strategies to allow for a consideration of national contexts where resources could be considered comparatively more or less readily available to protect health workers. Stratification by WHO region was also important because these regions, while large and often heterogenous in nature, do constitute administrative units with an important governance role to play during the emergence of global outbreaks and pandemics.

It should also be acknowledged that differing perceptions of risks and mitigation measures around the world may be influenced by different HCW training and education standards, cultural nuances, and institutional expectations. For example, Senthi and colleagues observed that workers in India found a high prevalence of workers unable to identify even immediate risks in an evidently hazardous environment [64]. Studies in the Middle East also reported gaps between

For peer review only - http://bmjopen.bmj.com/site/about/guidelines.xhtml

actual hazards and HCW recognition [65,66]. Ndejjo and colleagues report similar findings in Uganda and across sub-Saharan Africa [67].

CONCLUSION

This study adds to the literature on how risks become unevenly distributed, focusing here on country income level but also on within-country income inequality. As noted by Gostin et al., 2020 [68], the WHO has an important role in supporting LMICs with technical guidance and operational assistance, while simultaneously meeting the needs of high-income countries for information sharing, research coordination, and convening authorities, despite lacking both the authority and the resources to mount a more effective response to a global emergency such as this. Our study strongly suggests that international agencies with mandates related to fair trading practices and economic aid have to step up to address the disparities that threaten the healthcare workforce, and ensure that there is sufficient resilience to retain health workers needed for broader delivery of health services. It is also a matter of social justice that they do so.

For peer review only

Acknowledgements The authors would like to thank all members of the WHO Occupational Health Ad Hoc Committee as well as all the WHO Occupational Health Collaborating Centres around the world who contributed to the dissemination of the survey.

Contributors SH, AY and JS conceptualised the research. AY and MZ participated in preparation of the original survey and its use. SH prepared the data and conducted the initial analysis. SH and JMS conducted subsequent data analyses and data interpretation. VLT conducted literature reviews. SH wrote the initial manuscript draft. SH, AY, VT, MZ and JMS reviewed and edited the manuscript. AY and JMS accept full responsibility for the finished work and/or the conduct of the study as guarantors, had access to the data, and controlled the decision to publish. All authors read and approved the final manuscript.

Competing interests None declared.

Funding This article has received funding from the International Development Research Centre (M20-00559) and the Canadian Institutes of Health Research (VS1-175519).

Disclaimer The funders did not play a role in the decision to publish the article.

Data availability statement Data are available on reasonable request. Anonymised data that underlie the results reported in this article are available on justified request to the corresponding author.

Patient consent for publication Not required.

Ethics approval This study involves human participants and was approved by the Behavioural Research Ethics Board of the University of British Columbia (Ref. H20-01825)
As the survey was designed to be completed and submitted anonymously, no formal request for signed consent was solicited, with participants' submission itself indicating consent to use the information provided as anonymized aggregated data.

For peer review only

APPENDIX

Study questionnaire. (Taken from Report on preliminary findings – ref 14.)
(Pdf)

ONLINE SUPPLEMENTAL MATERIAL

SI Dataset. Data underlying the results of the study.
(Excel Data file (XLS). *– to be provided through Dryad data depository*

SI Figure. All supplemental figures.
Figure S1 - Study Population diagram
(pdf)

SI Table. All supplemental tables.
(pdf)

Table S1 - Study population demographics

Table S2 - Summary of factors and their constituent question areas

Table S3 – Variation in factor scores by gender and occupation

Table S4 - Responses to health and safety risk questions

Table S5 - Responses to mitigation measure questions

Table S6 – Factors associated with female patient care providers perceived risk acceptability and mitigation adequacy

Table S7 – Sensitivity analysis considering minimum sample size for calculating country mean values

For peer review only

REFERENCES

1 International Council of Nurses. ICN calls for data on healthcare worker infection rates and deaths. ICN - International Council of Nurses. https://www.icn.ch/news/icn-calls-data-healthcare-worker-infection-rates-and-deaths (accessed 12 Sep 2022).

2 World Health Organization. World Day for Safety and Health at Work: WHO key facts & key messages to support the day. https://www.who.int/news-room/detail/28-04-2020-who-calls-for-healthy-safe-and-decent-working-conditions-for-all-health-workers-amidst-covid-19-pandemic (accessed 12 Aug 2020).

3 Talisuna A, Yoti Z, Lee C, *et al.* The urgent need for a global commitment to protect healthcare workers. *BMJ Glob Health* 2020;**5**:e004077. doi:10.1136/bmjgh-2020-004077

4 Lancet T. COVID-19: protecting health-care workers. *The Lancet* 2020;**395**:922. doi:10.1016/S0140-6736(20)30644-9

5 Amnesty International. Amnesty analysis reveals over 7,000 health workers have died from COVID-19. https://www.amnesty.org/en/latest/news/2020/09/amnesty-analysis-7000-health-workers-have-died-from-covid19/ (accessed 24 Sep 2020).

6 The World Health Organization. World Health Organization (WHO) on Twitter. Twitter. https://twitter.com/WHO/status/1284148139797209093 (accessed 13 Oct 2020).

7 Papoutsi E, Giannakoulis VG, Ntella V, *et al.* Global burden of COVID-19 pandemic on healthcare workers. *ERJ Open Research* 2020;**6**. doi:10.1183/23120541.00195-2020

8 World Health Organization. Coronavirus disease 2019 (COVID-19) Situation Report – 82.

9 World Health Organization. Global Spending on Health: A World in Transition. WHO. https://www.who.int/publications/i/item/WHO-HIS-HGF-HFWorkingPaper-19.4 (accessed 12 May 2022).

10 World Health Organization. The World Health Report 2006 - working together for health. https://apps.who.int/iris/handle/10665/43432 (accessed 12 May 2022).

11 The World Bank. Physicians (per 1,000 people). https://data.worldbank.org/indicator/SH.MED.PHYS.ZS?most_recent_value_desc=false (accessed 24 Sep 2020).

12 McDiarmid MA. Hazards of the Health Care Sector: Looking Beyond Infectious Disease. *Annals of Global Health* 2014;**80**:315–9. doi:10.1016/j.aogh.2014.08.001

13 Caeiro JP, Garzón MI. Controlling infectious disease outbreaks in low-income and middle-income countries. *Curr Treat Options Infect Dis* 2018;**10**:55–64. doi:10.1007/s40506-018-0154-z

For peer review only

14 Mossburg S, Agore A, Nkimbeng M, *et al.* Occupational Hazards among Healthcare Workers in Africa: A Systematic Review. *Ann Glob Health* 2019;**85**. doi:10.5334/aogh.2434

15 Gross JV, Mohren J, Erren TC. COVID-19 and healthcare workers: a rapid systematic review into risks and preventive measures. *BMJ Open* 2021;**11**:e042270. doi:10.1136/bmjopen-2020-042270

16 Harrigan SP, Tsang VWL, Spiegel JM, *et al.* Health and safety of health workers in the context of COVID-19: A global survey. WHO Collaborating Centre, University of British Columbia http://med-fom-ghrp-spph.sites.olt.ubc.ca/files/2020/09/WHO_HCW_Survey_COVID-19-report_MASTER.pdf

17 Nowatzki NR. Wealth inequality and health: a political economy perspective. *Int J Health Serv* 2012;**42**:403–24. doi:10.2190/HS.42.3.c

18 Subramanian SV, Kawachi I. Income Inequality and Health: What Have We Learned So Far? *Epidemiol Rev* 2004;**26**:78–91. doi:10.1093/epirev/mxh003

19 Pickett KE, Wilkinson RG. Income inequality and health: a causal review. *Soc Sci Med* 2015;**128**:316–26. doi:10.1016/j.socscimed.2014.12.031

20 Fujishiro K, Ahonen EQ, Gimeno Ruiz de Porras D, *et al.* Sociopolitical values and social institutions: Studying work and health equity through the lens of political economy. *SSM Popul Health* 2021;**14**:100787. doi:10.1016/j.ssmph.2021.100787

21 Adu PA, Spiegel JM, Yassi A. Towards TB elimination: how are macro-level factors perceived and addressed in policy initiatives in a high burden country? *Globalization and Health* 2021;**17**:11. doi:10.1186/s12992-020-00657-1

22 Gelfand MJ, Jackson JC, Pan X, *et al.* The relationship between cultural tightness–looseness and COVID-19 cases and deaths: a global analysis. *The Lancet Planetary Health* 2021;**5**:e135–44. doi:10.1016/S2542-5196(20)30301-6

23 Haug N, Geyrhofer L, Londei A, *et al.* Ranking the effectiveness of worldwide COVID-19 government interventions. *Nat Hum Behav* 2020;**4**:1303–12. doi:10.1038/s41562-020-01009-0

24 Brauner JM, Mindermann S, Sharma M, *et al.* Inferring the effectiveness of government interventions against COVID-19. *Science* 2021;**371**. doi:10.1126/science.abd9338

25 Rinaldi C, Bekker MPM. A Scoping Review of Populist Radical Right Parties' Influence on Welfare Policy and its Implications for Population Health in Europe. *International Journal of Health Policy and Management* 2021;**10**:141–51. doi:10.34172/ijhpm.2020.48

26 World Health Organization. Definition of regional groupings. WHO. http://www.who.int/healthinfo/global_burden_disease/definition_regions/en/ (accessed 12 Aug 2020).

For peer review only

For peer review only - http://bmjopen.bmj.com/site/about/guidelines.xhtml

27 The World Bank. World Bank Country and Lending Groups – World Bank Data Help Desk. https://datahelpdesk.worldbank.org/knowledgebase/articles/906519-world-bank-country-and-lending-groups (accessed 12 Aug 2020).

28 The World Bank. GINI index (World Bank estimate). https://data.worldbank.org/indicator/SI.POV.GINI (accessed 12 Aug 2020).

29 Ritchie H, Ortiz-Ospina E, Beltekian D, *et al.* Coronavirus (COVID-19) Testing - Statistics and Research. Our World in Data. https://ourworldindata.org/coronavirus-testing (accessed 30 Nov 2020).

30 Taber KS. The Use of Cronbach's Alpha When Developing and Reporting Research Instruments in Science Education. *Res Sci Educ* 2018;**48**:1273–96. doi:10.1007/s11165-016-9602-2

31 R Core Team. *R: A language and environment for statistical computing*. R Foundation for Statistical Computing, Vienna, Austria. 2013. http://www.R-project.org/

32 IBM Corp. *SPSS*. 2018.

33 Shreffler J, Petrey J, Huecker M. The Impact of COVID-19 on Healthcare Worker Wellness: A Scoping Review. *West J Emerg Med* 2020;**21**:1059–66. doi:10.5811/westjem.2020.7.48684

34 Mhango M, Dzobo M, Chitungo I, *et al.* COVID-19 Risk Factors Among Health Workers: A Rapid Review. *Safety and Health at Work* 2020;**11**:262–5. doi:10.1016/j.shaw.2020.06.001

35 Bandyopadhyay S, Baticulon RE, Kadhum M, *et al.* Infection and mortality of healthcare workers worldwide from COVID-19: a systematic review. *BMJ Glob Health* 2020;**5**:e003097. doi:10.1136/bmjgh-2020-003097

36 Afulani PA, Nutor JJ, Agbadi P, *et al.* Job satisfaction among healthcare workers in Ghana and Kenya during the COVID-19 pandemic: Role of perceived preparedness, stress, and burnout. *PLOS Global Public Health* 2021;**1**:e0000022. doi:10.1371/journal.pgph.0000022

37 Gómez-Ochoa SA, Franco OH, Rojas LZ, *et al.* COVID-19 in Healthcare Workers: A Living Systematic Review and Meta-analysis of Prevalence, Risk Factors, Clinical Characteristics, and Outcomes. *Am J Epidemiol* Published Online First: 1 September 2020. doi:10.1093/aje/kwaa191

38 Zungu M, Voyi K, Mlangeni N, *et al.* Organizational factors associated with health worker protection during the COVID-19 pandemic in four provinces of South Africa. *BMC Health Services Research* 2021;**21**:1080. doi:10.1186/s12913-021-07077-w

39 Jeleff M, Traugott M, Jirovsky-Platter E, *et al.* Occupational challenges of healthcare workers during the COVID-19 pandemic: a qualitative study. *BMJ Open* 2022;**12**:e054516. doi:10.1136/bmjopen-2021-054516

For peer review only

40 Pappa S, Ntella V, Giannakas T, *et al.* Prevalence of depression, anxiety, and insomnia among healthcare workers during the COVID-19 pandemic: A systematic review and meta-analysis. *Brain Behav Immun* 2020;**88**:901–7. doi:10.1016/j.bbi.2020.05.026

41 Krishnamoorthy Y, Nagarajan R, Saya GK, *et al.* Prevalence of psychological morbidities among general population, healthcare workers and COVID-19 patients amidst the COVID-19 pandemic: A systematic review and meta-analysis. *Psychiatry Res* 2020;**293**:113382. doi:10.1016/j.psychres.2020.113382

42 Salazar de Pablo G, Vaquerizo-Serrano J, Catalan A, *et al.* Impact of coronavirus syndromes on physical and mental health of health care workers: Systematic review and meta-analysis. *J Affect Disord* 2020;**275**:48–57. doi:10.1016/j.jad.2020.06.022

43 Brier ND, Stroobants S, Vandekerckhove P, *et al.* Factors affecting mental health of health care workers during coronavirus disease outbreaks (SARS, MERS & COVID-19): A rapid systematic review. *PLOS ONE* 2020;**15**:e0244052. doi:10.1371/journal.pone.0244052

44 Dye TD, Alcantara L, Siddiqi S, *et al.* Risk of COVID-19-related bullying, harassment and stigma among healthcare workers: an analytical cross-sectional global study. *BMJ Open* 2020;**10**:e046620. doi:10.1136/bmjopen-2020-046620

45 Atnafie SA, Anteneh DA, Yimenu DK, *et al.* Assessment of exposure risks to COVID-19 among frontline health care workers in Amhara Region, Ethiopia: A cross-sectional survey. *PLoS One* 2021;**16**:e0251000. doi:10.1371/journal.pone.0251000

46 Arslanca T, Fidan C, Daggez M, *et al.* Knowledge, preventive behaviors and risk perception of the COVID-19 pandemic: A cross-sectional study in Turkish health care workers. *PLoS One* 2021;**16**:e0250017. doi:10.1371/journal.pone.0250017

47 Conti C, Fontanesi L, Lanzara R, *et al.* Fragile heroes. The psychological impact of the COVID-19 pandemic on health-care workers in Italy. *PLoS One* 2020;**15**:e0242538. doi:10.1371/journal.pone.0242538

48 Garralda Fernandez J, Molero Vilches I, Bermejo Rodríguez A, *et al.* Impact of SARS-CoV-2 pandemic among health care workers in a secondary teaching hospital in Spain. *PLoS One* 2021;**16**:e0245001. doi:10.1371/journal.pone.0245001

49 Shone E. More than 850 health and social care workers have died of Covid in England and Wales since the pandemic began. The Scotsman. https://www.scotsman.com/health/coronavirus/more-than-850-health-and-social-care-workers-have-died-of-covid-in-england-and-wales-since-the-pandemic-began-3114202 (accessed 10 Jun 2021).

50 Our key findings about US healthcare worker deaths in the pandemic's first year. The Guardian. https://www.theguardian.com/us-news/ng-interactive/2020/dec/22/lost-on-the-frontline-our-findings-to-date (accessed 10 Jun 2021).

For peer review only

51 Kavanagh MM, Gostin LO, Sunder M. Sharing Technology and Vaccine Doses to Address Global Vaccine Inequity and End the COVID-19 Pandemic. *JAMA* 2021;**326**:219–20. doi:10.1001/jama.2021.10823

52 Lim DW, Htun HL, Wang Y, *et al.* Healthcare workers as "canaries" for acute respiratory infections and pathogens during the COVID-19 pandemic. *J Hosp Infect* 2021;**112**:119–20. doi:10.1016/j.jhin.2021.03.019

53 McMahon DE, Peters GA, Ivers LC, *et al.* Global resource shortages during COVID-19: Bad news for low-income countries. *PLOS Neglected Tropical Diseases* 2020;**14**:e0008412. doi:10.1371/journal.pntd.0008412

54 Shears P, O'Dempsey TJD. Ebola virus disease in Africa: epidemiology and nosocomial transmission. *Journal of Hospital Infection* 2015;**90**:1–9. doi:10.1016/j.jhin.2015.01.002

55 Kim H, Hegde S, LaFiura C, *et al.* Access to personal protective equipment in exposed healthcare workers and COVID-19 illness, severity, symptoms and duration: a population-based case-control study in six countries. *BMJ Glob Health* 2021;**6**:e004611. doi:10.1136/bmjgh-2020-004611

56 Blázquez-Fernández C, Cantarero-Prieto D, Pascual-Saez M. Does Rising Income Inequality Reduce Life Expectancy? New Evidence for 26 European Countries (1995–2014). *Global Economic Review* 2018;**47**:464–79. doi:10.1080/1226508X.2018.1526098

57 Mellor JM, Milyo J. Is Exposure to Income Inequality a Public Health Concern? Lagged Effects of Income Inequality on Individual and Population Health. *Health Serv Res* 2003;**38**:137–51. doi:10.1111/1475-6773.00109

58 Odusanya IA, Akinlo AE. Income Inequality and Population Health in Sub-Saharan Africa: A Test of Income Inequality-Health Hypothesis. *Journal of Population and Social Studies [JPSS]* 2021;**29**:235–54.

59 Kim I-H, Muntaner C, Vahid Shahidi F, *et al.* Welfare states, flexible employment, and health: A critical review. *Health Policy* 2012;**104**:99–127. doi:10.1016/j.healthpol.2011.11.002

60 Sandal A, Yildiz AN. COVID-19 as a Recognized Work-Related Disease: The Current Situation Worldwide. *Saf Health Work* 2021;**12**:136–8. doi:10.1016/j.shaw.2021.01.001

61 McCartney G, Hearty W, Arnot J, *et al.* Impact of Political Economy on Population Health: A Systematic Review of Reviews. *Am J Public Health* 2019;**109**:e1–12. doi:10.2105/AJPH.2019.305001

62 Flynn MA, Check P, Steege AL, *et al.* Health Equity and a Paradigm Shift in Occupational Safety and Health. *Int J Environ Res Public Health* 2021;**19**:349. doi:10.3390/ijerph19010349

For peer review only - http://bmjopen.bmj.com/site/about/guidelines.xhtml

63 Loewenson R. Rethinking the Paradigm and Practice of Occupational Health in a World Without Decent Work: A Perspective From East and Southern Africa. *New Solut* 2021;**31**:107–12. doi:10.1177/10482911211017106

64 Senthil A, Anandh B, Jayachandran P, *et al.* Perception and prevalence of work-related health hazards among health care workers in public health facilities in southern India. *Int J Occup Environ Health* 2015;**21**:74–81. doi:10.1179/2049396714Y.0000000096

65 Muralidhar S, Singh PK, Jain RK, *et al.* Needle stick injuries among health care workers in a tertiary care hospital of India. *Indian J Med Res* 2010;**131**:405–10.

66 Kotwal A, Taneja D. Health Care Workers and Universal Precautions: Perceptions and Determinants of Non-compliance. *Indian J Community Med* 2010;**35**:526–8. doi:10.4103/0970-0218.74373

67 Ndejjo R, Musinguzi G, Yu X, *et al.* Occupational Health Hazards among Healthcare Workers in Kampala, Uganda. *Journal of Environmental and Public Health*. 2015;**2015**:e913741. doi:https://doi.org/10.1155/2015/913741

68 Gostin LO, Moon S, Meier BM. Reimagining Global Health Governance in the Age of COVID-19. *Am J Public Health* 2020;**110**:1615–9. doi:10.2105/AJPH.2020.305933

For peer review only

For peer review only - http://bmjopen.bmj.com/site/about/guidelines.xhtml

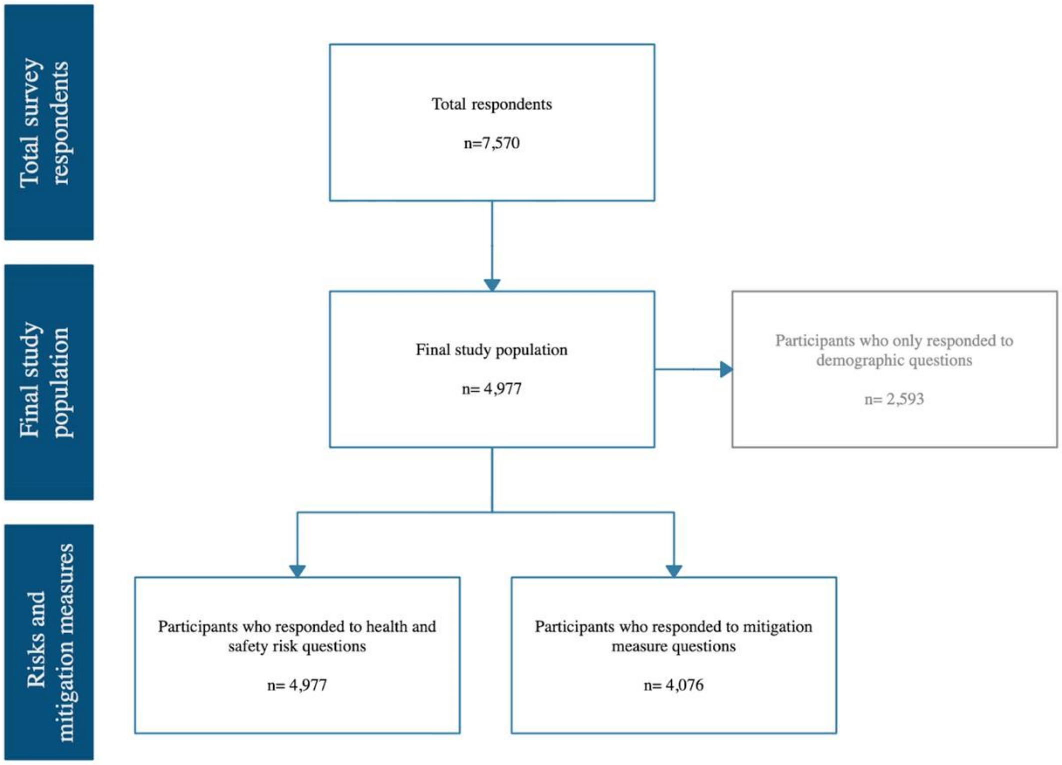
Total survey respondents
Final study population
Risks and mitigation measures
Total respondents
n=7,570
Final study population
n= 4,977
Participants who only responded to demographic questions
n= 2,593
Participants who responded to health and safety risk questions
n= 4,977
Participants who responded to mitigation measure questions
n= 4,076

Appendices

Survey instrument

Health and safety of health workers in COVID-19
Welcome to the survey on health and safety of health workers in COVID-19
Dear colleague,

This survey aims to identify the most common occupational risks for the health and safety of health workers and the measures for their prevention in the context of the ongoing pandemic of Corona Virus Infectious Disease (COVID-19).

In this survey we are interested in the health and safety of all health workers - all people engaged in the promotion, protection or improvement of the health of the population. This includes health workers involved in direct patient care, both formal and informal, in public and private facilities, including traditional medicine, as well as other assisting and supporting staff, including administration, management, ambulance drivers, public health workers, community health workers, and others.

The survey is intended for health workers, managers, and practitioners providing services for protecting the health and safety of workers in health facilities. The results will be used to inform action at all levels for improving the protection of health and safety of health workers in the ongoing response to COVID-19.

The survey has been developed by an international group of experts convened by the World Health Organization and the International Labour Organization and should take approximately 7 minutes to complete.

Your answers are completely confidential, and the data will be processed and analyzed in a way that will not link your answers to your identity.

B. About yourself and your area of work

1. In what country do you usually work?
Standard list of all countries in the world
Other (please specify)

* 2. Your gender?
Male
Female
Other
Prefer not to answer

For peer review only - http://bmjopen.bmj.com/site/about/guidelines.xhtml

3. What is your primary area of work? (responses below were randomized)

Administration and clerical support
Allied health professional
Community health worker
Infection prevention and control
Management and human resources
Mental health and psychosocial support
Occupational and environmental health
Patient care (medicine, nursing, midwifery, dentistry)
Pharmacy
Public health
Support staff – cleaner, driver, food worker
Other

4. You work most of the time for: (responses below were randomized)

Academia, research
Business enterprise or farm·
Employers' association /hospital federation
Healthcare facility - hospital, primary health-care centre, isolation camp
Local community
National government agency
Other
Professional association
Social care facility (e.g. nursing home, home care)·
Sub-national (provincial, district) authority
Trade union

C. Risks for health and safety of health workers
Think about the working conditions of health workers in your country, jurisdiction or health facility - those that you are most familiar with. No workplace is without risk, but some risks are negligible, or acceptable for a short time, and some are not acceptable at all. Below are some common risks for the health and safety of health workers; we are asking you to rate the current level of these risks, now during the COVID-19 pandemic.

For peer review only - http://bmjopen.bmj.com/site/about/guidelines.xhtml

5. How would you rate the level of these risks for health workers, now? (randomized)

Questions	Risk is negligible	Risk is acceptable for a short time	Risk is not acceptable at all	Don't know/Unsure
Skin damage from personal protective equipment and/or frequent hand hygiene				
Needle-sticks and sharps injuries				
Inadequate sanitation facilities				
Insufficient access to facilities for personal hygiene, such as, shower and menstrual hygiene				
Exposure to blood, body fluids, respiratory secretions, and other potentially infectious materials				

6. How would you rate the level of these risks for health workers, now? (randomized)

Questions	Risk is negligible	Risk is acceptable for a short time	Risk is not acceptable at all	Don't know/Unsure
Back injury from manual handling of patients and heavy objects				
Hazardous chemicals, drugs, cleaning and disinfection agents				
Slips, trips, and falls				
Crowded workplace				
Thermal discomfort (cold, heat, humidity)				

7. How would you rate the level of these risks for health workers, now? (randomized)

Questions	Risk is negligible	Risk is acceptable for a short time	Risk is not acceptable at all	Don't know/Unsure
Physical violence and assaults				
Bullying or psychological harassment at the workplace				
Sexual harassment				

For peer review only - http://bmjopen.bmj.com/site/about/guidelines.xhtml

8. How would you rate the level of these risks for health workers, now? (randomized)

Questions	Risk is negligible	Risk is acceptable for a short time	Risk is not acceptable at all	Don't know/Unsure
Regular long working hours (more than 48 hours a week)				
Time pressure, high workload				
Shift work with night shifts				
Insufficient time-off duty to rest (less than 11 hours between shifts)				

9. How would you rate the level of these risks for health workers, now? (randomized)

Questions	Risk is negligible	Risk is acceptable for a short time	Risk is not acceptable at all	Don't know/Unsure
Skin damage from personal protective equipment and/or frequent hand hygiene				
Needle-sticks and sharps injuries				
Inadequate sanitation facilities				
Insufficient access to facilities for personal hygiene, such as, shower and menstrual hygiene				
Exposure to blood, body fluids, respiratory secretions, and other potentially infectious materials				

D. Preventive measures

There are measures for the prevention of most risks for health and safety at work, but these measures may not be fully implemented and not all workers may benefit from these measures. Think again about the working conditions of health workers in your country, jurisdiction or health facility - those that you are most familiar with. The following questions are about the preventive measures for their health and safety in the real situation, now, during the COVID-19 pandemic.

For peer review only - http://bmjopen.bmj.com/site/about/guidelines.xhtml

10. How would you rate the level of application of these measures in the health services according to your knowledge? (randomized)

Questions	Does not exist at all	Exists and offers some protection	Exists and offers full protection	Don't know/Unsure
Policy for infection prevention and control in the health facility				
Processes for triage of patient in place at the emergency room, including early detection and isolation of infectious patients				
Routine assessment of the risk of exposure to body substances or contaminated surfaces before any health care activity and use of appropriate measures for personal protection				
Regular environmental clean-up and disinfection				
Immunization of health workers				

For peer review only

11. How would you rate the level of application of these measures in the health services according to your knowledge? (randomized)

Questions	Does not exist at all	Exists and offers some protection	Exists and offers full protection	Don't know/Unsure
Reporting of incidental exposures to blood, body fluids, or respiratory secretions				
Policies in place for post-exposure prophylaxis, such as, for HIV, Hepatitis B				
Facilities for hand hygiene (hand washing and disinfection) are readily available				
Personal protective equipment, such as masks, gloves, goggles, gowns are readily available				
Training and education of workers about infection prevention and control				

For peer review only - http://bmjopen.bmj.com/site/about/guidelines.xhtml

12. How would you rate the level of application of these measures in health services according to your knowledge? (randomized)

Questions	Does not exist at all	Exists and offers some protection	Exists and offers full protection	Don’t know/Unsure
Prevention of workplace violence and security measures				
Management of working time, rest and recuperation				
Workplace policies against bullying, psychological and sexual harassment				
Human resource management of safe staffing and workload				
Psycho-social support and counselling				

13. How would you rate the level of application of these measures in the health services according to your knowledge? (randomized)

Questions	Does not exist at all	Exists and offers some protection	Exists and offers full protection	Don’t know/Unsure
Occupational safety and health policy and management system in the facility				
Regular assessment of workplace health and safety risks and controls				
Engineering controls, such as ventilation, physical barriers, safer devices				
Ergonomic workplace design and furniture				
Devices for patient handling and lifting of loads				

14. How would you rate the level of application of these measures in the health services according to your knowledge? (randomized)

Questions	Does not exist at all	Exists and offers some protection	Exists and offers full protection	Don’t know/Unsure
Regular medical check-ups of health workers				
Medical first aid kits				
Consultations between management and workers regarding health and safety at work				
Training and education of workers about occupational safety and health				

Other (please specify)

For peer review only - http://bmjopen.bmj.com/site/about/guidelines.xhtml

Table A 1: Survey options for occupation

Group	Survey responses
Patient care/health services	Patient care (medicine, nursing, midwifery, dentistry)
	Allied health professional
	Mental health and psychosocial support
	Pharmacy
	Community health worker
Specialized support	Occupational and environmental health
	Public health
	Infection prevention and control
	Support staff – cleaner, driver, food worker
Clerical support/administration and management	Administration and clerical support
	Management and human resources

Table A 2: Comparison between participants who answered risk and mitigation questions

Variables		Those who responded to risk questions (n=4977)	Those who responded to mitigation measure questions (n=4076)
Countries by region	AFRO	10%	11%
	EMRO	4%	4%
	EURO	35%	36%
	PAHO	31%	31%
	SEARO	3%	3%
	WPRO	15%	16%
Economic Class	High	59%	60%
	Lower-middle	10%	10%
	Upper-middle	27%	26%
	Low	4%	4%
Sex	Male	33%	34%
	Female	65%	65%
	Other / prefer not to answer	1%	1%
Occupation	Patient care/health services	56%	58%
	Specialized support	29%	29%
	Clerical support/administration and management	7%	7%
	Other	8%	7%

For peer review only - http://bmjopen.bmj.com/site/about/guidelines.xhtml

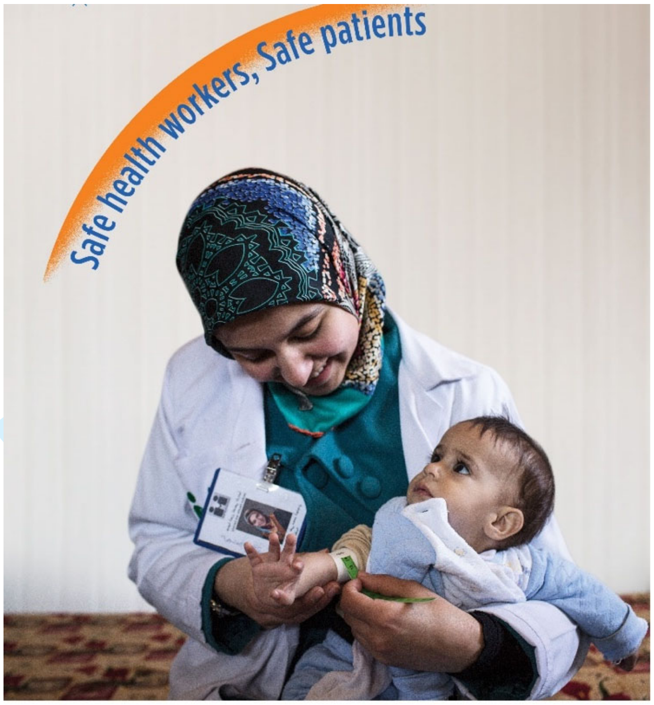

Speak up
for health worker safety!

World
Patient Safety
Day 17 September 2020

Health and safety of health workers in the context of COVID-19: A global survey

Sean P. Harrigan, Vivian W. L. Tsang, Jerry M. Spiegel, Annalee Yassi

September 15, 2020

a place of mind
THE UNIVERSITY OF BRITISH COLUMBIA
Faculty of Medicine
School of Population and Public Health

GLOBAL HEALTH
RESEARCH PROGRAM

WHO Collaborating Centre
for Occupational Health

© Global Health Research Program, School of Population and Public Health,

For peer review only - http://bmjopen.bmj.com/site/about/guidelines.xhtml

ONLINE SUPPLEMENTARY INFORMATION

Table S1. Study population demographics

Variables		n	%
Total number of participants		4977	100%
Total who replied to the health and safety risks for health workers questions		4977	100%
Total who replied to the mitigation measures questions		4076	82%
Total number of countries		161	100%
Countries by region	African region	516	10%
	Americas region	1565	31%
	Eastern Mediterranean region	221	4%
	European region	1757	35%
	South East Asian region	158	3%
	Western Pacific region	760	15%
Economic Class	High	2960	59%
	Lower-middle	480	10%
	Upper-middle	1324	27%
	Low	213	4%
Sex	Male	1654	33%
	Female	3259	65%
	Other / prefer not to answer	64	1%
Occupation	Patient care/health services	2805	56%
	Specialized support	1426	29%
	Clerical support/administration and management	341	7%
	Other	405	10%
Type of employer	Health services	3038	61%
	Government services	758	15%
	Business and farms	473	10%
	Academia	367	7%
	Professional associations	177	4%
	International organization/NGO and non-for-profit	51	1%
	Other	113	2%

For peer review only - http://bmjopen.bmj.com/site/about/guidelines.xhtml

Table S2. Summary of Factors and their constituent question areas

Survey area of focus	Factor	Description of question
Health and Safety Risks	Work Environment (Factor 1)	Blood and bodily fluids exposure
		Skin damage from PPE and hand hygiene
		Needlesticks and sharps injuries
		Sanitation facilities
		Personal hygiene
		Thermal discomfort
		Crowded workplace
		Slips trips & falls
		Back injury form heavy lifting
		Chemicals
		Bullying & harassment
		Sexual harassment
	Work Organization (Factor 2)	Physical violence and assaults
		Time pressure
		Shift work
		Long working hours
		Insufficient rest
Mitigation measures	Infection protection and control (Factor 3)	IPC policy
		Patient triage
		Standard precautions
		Cleanup & disinfection
		Immunization
		Reporting of blood exposure
		Policies for post-exposure prophylaxis
		Hand hygiene
		PPE
		IPC training
	Occupational health and safety (Factor 4)	OSH policy
		Occupational health and safety risk assessment
		Occupational health and safety engineering controls
		Ergonomic workplace design and furniture
		Safe patient handling
		Violence prevention
		Management of working time & rest
		Policy harassment
		Safe staffing & workload
		Psychosocial support
		Medical checkups
		Medical first aid kits
		Labour management consultation on OSH
		Occupational health and safety training

For peer review only

For peer review only - http://bmjopen.bmj.com/site/about/guidelines.xhtml

Table S3: Detailed breakdowns in individual level responses by occupation and gender

a. Detailed breakdowns in responses by occupation showing gender differences

		Risk acceptability				Mitigation adequacy			
		Work environment		Work organization		IPC		OHS [f3]	
Population characteristic	n^c	mean	p	mean	p	mean	p	mean	P
All Occupations	4916	**3.88**	**0.04**	3.87	0.101	**4.79**	**<0.01**	6.28	0.19
m		***4.11***	***<0.01***	*3.92*	*0.397*	*4.88*	*0.089*	*6.19*	*0.07*
f		***3.76***		*3.85*		*4.74*		*6.33*	
- Patient Care	2792	**3.92**	***0.001***	3.88	*0.296*	**4.63**	*0.76*	6.27	*0.092*
m		***4.19***		*3.96*		*4.65*		*6.15*	
f		***3.80***		*3.84*		*4.62*		*6.32*	
- Specialist	1404	**3.84**	*0.129*	3.80	*0.669*	**4.90**	*0.218*	6.30	*0.193*
m		*3.97*		*3.77*		*5.00*		*6.20*	
f		*3.73*		*3.83*		*4.81*		*6.39*	
- Admin-Mgr	327	**4.14**	*0.06*	4.22	*0.632*	**5.50**	*0.512*	6.50	*0.872*
m		*4.65*		*4.33*		*5.65*		*6.54*	
f		*3.92*		*4.17*		*5.43*		*6.48*	
- Other	393	**3.55**	*0.205*	3.82	*0.189*	**5.03**	*0.20*	6.08	*0.89*
m		*3.82*		*4.10*		*5.30*		*6.11*	
f		*3.43*		*3.69*		*4.90*		*6.08*	

Note: Statistical significance at p<.05 level indicated by bold; statistical significance at p<.10 level indicated by underlining.
Italic font is used for breakdown analysis (i.e. by gender) of population attribute (regular font) being analyzed (i.e. by occupation) and values are right-justified.
Regular font is used for analysis of the population attribute (regular font) being analyzed (i.e. by occupation).
Green colour indicates significant value deemed as associated with less risk (i.e. less unacceptable exposure; more adequate mitigation.
Red colour indicates statistically significant value associated with more risk (i.e. more unacceptable exposure; less adequate mitigation.

For peer review only

For peer review only - http://bmjopen.bmj.com/site/about/guidelines.xhtml

b. Detailed breakdowns in responses by occupation showing gender differences

Population characteristic	n[c]	Risk acceptability: Work environment mean	p	Work organization mean	p	Mitigation adequacy: IPC mean	p	OHS [f3] mean	P
Total	4863	**3.88**	**<0.01***	3.87	0.4	4.79	0.09	6.28	0.07
Patient Care		***3.91***		*3.88*		*4.63*		*6.27*	
Specialist		***3.84***		*3.80*		*4.90*		*6.30*	
Admin-Mgr		***4.14***		*4.22*		*5.50*		*6.50*	
Other		***3.55***		*3.82*		*5.03*		*6.08*	
Female	3220	**3.76**	0.189	3.85	0.273	4.74	**<0.01***	6.33	0.288
Patient Care		*3.80*		*3.84*		***4.62***		*6.32*	
Specialist		*3.73*		*3.83*		*4.82*		*6.39*	
Admin-Mgr		*3.92*		*4.17*		***5.43***		*6.48*	
Other		*3.43*		*3.69*		*4.90*		*6.07*	
Male	1643	**4.11**	0.089	3.92	0.166	4.88	**0.001**	6.19	0.527
Patient Care		*4.19*		*3.96*		***4.65***		*6.15*	
Specialist		*3.97*		*3.77*		*5.00*		*6.20*	
Admin-Mgr		*4.65*		*4.33*		***5.65***		*6.54*	
Other		*3.82*		*4.10*		*5.30*		*6.11*	

Note: Statistical significance at p<.05 level indicated by bold; statistical significance at p<.10 level indicated by underlining.
Italic font is used for breakdown analysis (i.e. by occupation) of population attribute (regular font) being analyzed (i.e. by gender) and values are right-justified.
Regular font is used for analysis of the population attribute (regular font) being analyzed (i.e. by gender).
Green colour indicates significant value deemed as associated with less risk (i.e. less unacceptable exposure; more adequate mitigation).
Red colour indicates statistically significant value associated with more risk (i.e. more unacceptable exposure; less adequate mitigation).

For peer review only - http://bmjopen.bmj.com/site/about/guidelines.xhtml

Table S4. Responses to health and safety risk questions

Question	Risk is not acceptable at all	Risk is acceptable for a short time	Risk is negligible	Don't know/unsure
**Infectious risks**				
Exposure to blood, body fluids, respiratory secretions, and other potentially infectious materials	**52%**	29%	15%	4%
Skin damage from personal protective equipment and/or frequent hand hygiene	33%	**46%**	16%	5%
Needle-sticks and sharps injuries	**46%**	21%	26%	7%
Inadequate sanitation facilities	**52%**	21%	23%	4%
Insufficient access to facilities for personal hygiene, such as, shower and menstrual hygiene	**49%**	22%	23%	6%
**Physical work environment**				
Thermal discomfort (cold, heat, humidity)	25%	**46%**	24%	5%
Crowded workplace	**42%**	36%	18%	4%
Slips, trips, and falls	**34%**	26%	33%	7%
Back injury from manual handling of patients and heavy objects	**41%**	34%	19%	6%
Hazardous chemicals, drugs, cleaning and disinfection agents	**36%**	**36%**	22%	6%
**Psychosocial work environment**				
Bullying or psychological harassment at the workplace	**54%**	18%	21%	7%
Sexual harassment	**50%**	10%	31%	9%
Physical violence and assaults	**54%**	16%	24%	6%
**Work organization**				
Time pressure, high workload	38%	**49%**	10%	3%
Shift work with night shifts	23%	**48%**	21%	8%
Regular long working hours (more than 48 hours a week)	**38%**	42%	15%	5%
Insufficient time-off duty to rest (less than 11 hours between shifts)	**40%**	36%	18%	6%

Note: Most cited response highlighted in bold

For peer review only - http://bmjopen.bmj.com/site/about/guidelines.xhtml

Table S5. Responses to mitigation measure questions

Question	Does not exist at all	Exists and offers some protection	Exists and offers full protection	Don't know/ unsure
**Infection prevention and control**				
IPC policy in the health facility	8%	**60%**	28%	4%
Patient triage	9%	**54%**	28%	9%
Standard precautions	15%	**53%**	25%	7%
Regular environmental clean-up and disinfection	6%	**56%**	34%	4%
Immunization of health workers	19%	**46%**	26%	9%
Reporting of incidental exposures to blood, body fluids, or respiratory secretions	10%	**50%**	32%	8%
Policies in place for post-exposure prophylaxis, such as, for HIV, Hepatitis B	8%	41%	**42%**	9%
Facilities for hand hygiene (hand washing and disinfection) are readily available	3%	40%	**54%**	3%
Personal protective equipment, such as masks, gloves, goggles, gowns are readily available	8%	**55%**	34%	3%
Training and education of workers about infection prevention and control	11%	**54%**	32%	3%
**Occupational safety and health**				
Occupational safety and health policy and management system in the facility	14%	**58%**	22%	6%
Regular assessment of workplace health and safety risks and controls	22%	**51%**	21%	6%
Engineering controls, such as ventilation, physical barriers, safer devices	19%	**54%**	19%	8%
Ergonomic workplace design and furniture	33%	**48%**	12%	7%
Devices for patient handling and lifting of loads	27%	**48%**	15%	10%
Prevention of workplace violence and security measures	21%	**52%**	21%	6%
Management of working time, rest and recuperation	20%	**55%**	19%	6%
Workplace policies against bullying, psychological and sexual harassment	27%	**43%**	21%	9%
Human resource management of safe staffing and workload	24%	**52%**	16%	8%
Psycho-social support and counselling	32%	**46%**	15%	7%
Regular medical check-ups of health workers	33%	**43%**	18%	6%
Medical first aid kits	15%	**48%**	30%	7%
Consultations between management and workers regarding health and safety at work	25%	**50%**	19%	6%
Training and education of workers about occupational safety and health	20%	**54%**	21%	5%

Note: Most cited response highlighted in bold

For peer review only - http://bmjopen.bmj.com/site/about/guidelines.xhtml

Table S6: Comparing analyses of acceptability and adequacy in different stratified populations

	Patient Care Female		Patient Care All		Frontline[e] all		All occupations	
**Countries included**	**112**		**133**		**156**		**161**	
**Explanatory Variable** (organized by outcome area)	**OR[d]**	**p**	**OR[d]**	**p**	**OR[d]**	**p**	**OR[d]**	**p**
**Acceptable WP Enviro. Risk**								
Country Income Level [a]	0.81	0.736	0.63	0.415	1.01	0.981	1.24	0.708
**Gini Coefficient** [b]	0.98	0.609	0.96	0.1991	***0.94***	***0.068***	**0.92**	**0.012***
COVID-Log [c]	1.12	0.777	1.33	0.398	1.22	0.549	1.09	0.801
**Acceptable WP Org. Risk**								
Country Income Level [a]	1.30	0.657	1.57	0.446	1.06	0.926	0.71	0.52
**Gini Coefficient** [b]	***0.95***	***0.093***	**0.93**	**0.024***	***0.93***	***0.056***	**0.95**	**0.017***
**COVID-Log** [c]	**0.44**	**0.034***	0.66	0.248	0.98	0.951	0.89	0.710
**Adequate IPC mitigation**								
Country Income Level [a]	**11.25**	**0.004***	**7.48**	**0.006***	**6.92**	**0.001***	**6.61**	**0.001***
**Gini Coefficient** [b]	0.95	0.220	0.95	0.125	**0.95**	**0.047***	**0.94**	**0.025***
COVID-Log [c]	0.69	0.479	0.7	0.181	0.70	0.264	0.76	0.373
**Adequate OHS mitigation**								
Country Income Level [a]	**3.94**	**0.047***	**5.86**	**0.004***	**18.39**	**<0.001***	**10.91**	**<0.001***
**Gini Coefficient** [b]	**0.92**	**0.020***	***0.95***	***0.083***	0.99	0.755	0.99	0.779
COVID-Log [c]	0.79	0.556	0.69	0.281	0.99	0.988	1.08	0.816

[a] Country Income was coded as comparing "High and Upper-Middle Income" countries versus "Low and Lower-Middle Income" countries

[b] Gini coefficient was considered in the logistic regression analysis as a continuous variable;

[c] COVID levels where the log value of the rate of cases per million at the beginning of the survey (taken June 1, 2020); log values to smooth very high levels while taking variation into account

[d] Odds Ratios were calculated by assessing the likelihood (OR) of the presence of a mean scores ≥ 5 corresponding to assessments that mitigation "exists and offers some protection" or better; or level of risk is assessed as "risk is acceptable for a short time" or better.

[e] includes all patient care and workplace specialist support personnel such as OHS and IPC professionals

* Indicates statistical significance (p<0.05) and also bolded; ** p<.10 but >.05; indicated in italics and bold

For peer review only

Table S7: Comparing analyses when minimum country sample size provisions are applied

	Patient Care Female		Patient Care Female		Frontline[e]		Frontline[e]	
**Minimum country sample**	**2**		**all**		**4**		**all**	
**Countries included**	**80**		**112**		**101**		**161**	
**Explanatory Variable** (organized by outcome area)	**OR[d]**	**p**	**OR[d]**	**p**	**OR[d]**	**p**	**OR[d]**	**p**
**Acceptable WP Enviro. Risk**								
Country Income Level [a]	0.62	0.556	0.81	0.736	1.03	0.967	1.01	0.981
**Gini Coefficient** [b]	0.97	0.404	0.98	0.609	0.95	0.228	***0.94***	***0.068*****
COVID-Log	1.11	0.852	1.12	0.777	0.89	0.797	1.22	0.549
**Acceptable WP Org. Risk**								
Country Income Level [a]	1.2	0.830	1.30	0.657	1.33	0.736	1.06	0.926
**Gini Coefficient** [b]	0.97	0.487	***0.95***	***0.093*****	0.94	0.230	***0.93***	***0.056*****
COVID-Log	0.77	0.617	**0.44**	**0.034***	0.42	0.457	0.98	0.951
**Adequate IPC mitigation**								
Country Income Level [a]	**9.62**	**0.041***	**11.25**	**0.004***	**36.89**	**0.001***	**6.92**	**0.001***
**Gini Coefficient** [b]	0.94	0.223	0.95	0.220	0.98	0.647	**0.95**	**0.047***
COVID-Log	0.54	0.433	0.69	0.479	0.48	0.190	0.70	0.264
**Adequate OHS mitigation**								
Country Income Level [a]	3.00	0.153	**3.94**	**0.047***	**5.46**	**0.020***	**18.39**	**<0.001***
**Gini Coefficient** [b]	***0.94***	***0.077*****	**0.92**	**0.020***	***0.94***	***0.061*****	0.99	0.755
COVID-Log	0.67	0.392	0.79	0.556	0.56	0.152	0.99	0.988

[a] Country Income was coded as comparing "High and Upper-Middle Income" countries versus "Low and Lower-Middle Income" countries

[b] Gini coefficient was considered in the logistic regression analysis as a continuous variable;

[c] COVID levels where the log value of the rate of cases per million at the beginning of the survey (taken June 1, 2020); log values to smooth very high levels while taking variation into account

[d] Odds Ratios were calculated by assessing the likelihood (OR) of the presence of a mean scores ≥ 5 corresponding to assessments that mitigation "exists and offers some protection" or better; or level of risk is assessed as "risk is acceptable for a short time" or better.

[e] includes all patient care and workplace specialist support personnel such as OHS and IPC professionals

* Indicates statistical significance (p<0.05) and also bolded; ** p<.10 but >.05; indicated in italics and bold

For peer review only

For peer review only - http://bmjopen.bmj.com/site/about/guidelines.xhtml

STROBE Statement—Checklist of items that should be included in reports of ***cross-sectional studies***

	Item No	Recommendation	Page No
**Title and abstract**	1	(*a*) Indicate the study's design with a commonly used term in the title or the abstract	1 & 2
		(*b*) Provide in the abstract an informative and balanced summary of what was done and what was found	2-3
**Introduction**			
Background/rationale	2	Explain the scientific background and rationale for the investigation being reported	4-5
Objectives	3	State specific objectives, including any prespecified hypotheses	5
**Methods**			
Study design	4	Present key elements of study design early in the paper	5
Setting	5	Describe the setting, locations, and relevant dates, including periods of recruitment, exposure, follow-up, and data collection	6-7
Participants	6	(*a*) Give the eligibility criteria, and the sources and methods of selection of participants	6-7
Variables	7	Clearly define all outcomes, exposures, predictors, potential confounders, and effect modifiers. Give diagnostic criteria, if applicable	8-10
Data sources/ measurement	8*	For each variable of interest, give sources of data and details of methods of assessment (measurement). Describe comparability of assessment methods if there is more than one group	7-9
Bias	9	Describe any efforts to address potential sources of bias	9,10,17,25
Study size	10	Explain how the study size was arrived at	6, 11-12
Quantitative variables	11	Explain how quantitative variables were handled in the analyses. If applicable, describe which groupings were chosen and why	8-10
Statistical methods	12	(*a*) Describe all statistical methods, including those used to control for confounding	9-11
		(*b*) Describe any methods used to examine subgroups and interactions	9-11
		(*c*) Explain how missing data were addressed	9
		(*d*) If applicable, describe analytical methods taking account of sampling strategy	n/a
		(*e*) Describe any sensitivity analyses	25
**Results**			
Participants	13*	(a) Report numbers of individuals at each stage of study—eg numbers potentially eligible, examined for eligibility, confirmed eligible, included in the study, completing follow-up, and analysed	11
		(b) Give reasons for non-participation at each stage	6-7
		(c) Consider use of a flow diagram	Fig S1
Descriptive data	14*	(a) Give characteristics of study participants (eg demographic, clinical, social) and information on exposures and potential confounders	11-12
		(b) Indicate number of participants with missing data for each variable of interest	n/a

For peer review only - http://bmjopen.bmj.com/site/about/guidelines.xhtml

Outcome data	15*	Report numbers of outcome events or summary measures	
Main results	16	(*a*) Give unadjusted estimates and, if applicable, confounder-adjusted estimates and their precision (eg, 95% confidence interval). Make clear which confounders were adjusted for and why they were included	11-18
		(*b*) Report category boundaries when continuous variables were categorized	18-20
		(*c*) If relevant, consider translating estimates of relative risk into absolute risk for a meaningful time period	n/a
Other analyses	17	Report other analyses done—eg analyses of subgroups and interactions, and sensitivity analyses	18-20
**Discussion**			
Key results	18	Summarise key results with reference to study objectives	21-22
Limitations	19	Discuss limitations of the study, taking into account sources of potential bias or imprecision. Discuss both direction and magnitude of any potential bias	25-26
Interpretation	20	Give a cautious overall interpretation of results considering objectives, limitations, multiplicity of analyses, results from similar studies, and other relevant evidence	21-25
Generalisability	21	Discuss the generalisability (external validity) of the study results	26
**Other information**			
Funding	22	Give the source of funding and the role of the funders for the present study and, if applicable, for the original study on which the present article is based	7

*Give information separately for exposed and unexposed groups.

Note: An Explanation and Elaboration article discusses each checklist item and gives methodological background and published examples of transparent reporting. The STROBE checklist is best used in conjunction with this article (freely available on the Web sites of PLoS Medicine at http://www.plosmedicine.org/, Annals of Internal Medicine at http://www.annals.org/, and Epidemiology at http://www.epidem.com/). Information on the STROBE Initiative is available at www.strobe-statement.org.

For peer review only

For peer review only - http://bmjopen.bmj.com/site/about/guidelines.xhtml
