# Supplementary material for: Impacts of economic inequality on healthcare worker safety at the onset of the COVID-19 pandemic: cross-sectional analysis of a global survey
Source: BMJ Open. 2022 Oct 5;12(10):e064804. doi: 10.1136/bmjopen-2022-064804 (PMC9534779; doi:10.1136/bmjopen-2022-064804)
Supplement: Reviewer comments [file bmjopen-2022-064804.reviewer_comments.pdf]

PEER REVIEW HISTORY

BMJ Open publishes all reviews undertaken for accepted manuscripts. Reviewers are asked to complete a checklist review form (**http://bmjopen.bmj.com/site/about/resources/checklist.pdf**) and are provided with free text boxes to elaborate on their assessment. These free text comments are reproduced below.

ARTICLE DETAILS

**TITLE (PROVISIONAL)**	Impacts of economic inequality on healthcare worker safety at the onset of the COVID-19 pandemic: Cross-sectional analysis of a global survey
**AUTHORS**	Harrigan, Sean; Tsang, Vivian; Yassi, Annalee; Zungu, Muzimkhulu; Spiegel, Jerry

VERSION 1 – REVIEW

**REVIEWER**	Tsikrika, Stamatoula<br>National and Kapodistrian University of Athens - Faculty of Medicine
**REVIEW RETURNED**	03-Jun-2022

**GENERAL COMMENTS**	This article estimates the ımpacts of economic inequality on healthcare worker safety at the onset of the COVID-19 pandemic, a very interesting topic for frontline health care workers and first responders. The introduction is clear and concise and gives a good rationale of association with risk exposure acceptability and mitigation measure adequacy with- in country income disparity. All the figures and tables are easily readable, correct, and informative. This paper presents novelty and originality of statistic research, importance, and impact of COVID-19 on health inequalities not just in terms of virus-related infection and mortality, but also in terms of the health consequences of the policy responses undertaken in most countries. These state-imposed restrictions—usually requiring the government to take on emergency powers—have been implemented to varying levels of severity, but all have in common a significant increase in social isolation. It seems likely that there will be a post-COVID-19 global economic slump—which could make the health equity situation even worse, particularly if health-damaging policies of austerity are implemented again. It is vital that this time, right global health policy responses should be undertaken so that the COVID-19 pandemic does not increase health inequalities for future generations. No plagiarism detected, the references are up-to-date.<br>In my view, after carefully reading the article, I think the article can be approved for publication.

**REVIEWER**	Rubeshkumar, Polani<br>National Institute of Epidemiology
**REVIEW RETURNED**	12-Jun-2022

**GENERAL COMMENTS**	This article described the impact of economic inequality on healthcare worker safety at the onset of the COVID-19 pandemic. During the early days of the pandemic, the health system across

	the globe was overburdened. The study was conducted online and as expected the majority of the participants were from High-income countries. Skewed representations are unavoidable in these situations. I am glad about the author's efforts to minimise bias from planning to analysis. I do not see any major methodological or statistical queries. I wish this study could bring some policy change in favour of HCWs.

**REVIEWER**	Ng, Shu<br>University of Edinburgh
**REVIEW RETURNED**	06-Aug-2022

**GENERAL COMMENTS**	Thank you for the great effort in shedding crucial light on the impacts of economic inequality on healthcare worker safety and providing empirical evidence to support how economic inequalities impact HCW's overall well-being, especially during the early stages of the pandemic. The research will add to the literature supporting the global need to reduce income inequalities.<br>Just a minor clarification in the 'Discussion' section, who are the 'marginalised worker populations'? Are they health care workers from lower-middle and low-income countries, and why are they marginalised?<br>A minor grammatical error in the 'Limitation' section - 'Cross-country comparative studies such ours'.<br><br>Thank you.

VERSION 1 – AUTHOR RESPONSE

Response to reviewer 1:
We would like to thank this Dr. Tsikrika for sharing their insight and comments regarding the implications of our study. We appreciate you taking the time to review our manuscript.

REVIEWER #2: ***Dr. Polani Rubeshkumar, National Institute of Epidemiology***
This article described the impact of economic inequality on healthcare worker safety at the onset of the COVID-19 pandemic. During the early days of the pandemic, the health system across the globe was overburdened. The study was conducted online and as expected the majority of the participants were from High-income countries. Skewed representations are unavoidable in these situations. I am glad about the author's efforts to minimise bias from planning to analysis. I do not see any major methodological or statistical queries. I wish this study could bring some policy change in favour of HCWs.

Response to reviewer 2:
We would like to thank Dr. Rubeshkumar for their – we too wish this can have an impact on policy change in favour of HCWs.
REVIEWER #3: ***Dr. Shu Ng, University of Edinburgh***
Thank you for the great effort in shedding crucial light on the impacts of economic inequality on healthcare worker safety and providing empirical evidence to support how economic inequalities impact HCW's overall well-being, especially during the early stages of the pandemic. The research will add to the literature supporting the global need to reduce income inequalities. Just a minor

clarification in the 'Discussion' section, who are the 'marginalised worker populations'? Are they health care workers from lower-middle and low-income countries, and why are they marginalised? A minor grammatical error in the 'Limitation' section - 'Cross-country comparative studies such ours'.

Response to reviewer 3:
We appreciate the comments from Dr. Ng, we too hope this paper can add to the literature concerning inequities in healthcare.

We are also greatly appreciative of Dr. Ng's comment on providing more clarification regarding our reference to 'marginalised work populations' in the discussion section. We are referring to the tendency of the health and safety of HCWs to be systematically marginalized, even prior to COVID, and have re-worded this paragraph (lines 490 to 508) to better explain this and link the explanation of a possible pathway to the previous paragraph that spoke about the influence of policy orientations to "equity" concern.

Finally, in regards to the grammatical error, thank you for pointing this out, we have made the correction on line 500-501.

VERSION 2 – REVIEW

**REVIEWER**	Ng, Shu<br>University of Edinburgh
**REVIEW RETURNED**	06-Sep-2022

**GENERAL COMMENTS**	Thank you for your revision.
